# Large language models as uncertainty-calibrated optimizers for experimental discovery

**DOI:** 10.1038/s42256-026-01283-z

**Published:** 2026-08-28

**Authors:** Bojana Ranković, Ryan-Rhys Griffiths, Philippe Schwaller

**Affiliations:** 1 Institute of Chemical Sciences and Engineering, École Polytechnique Fédérale de Lausanne, Lausanne, Switzerland; 2 NCCR Catalysis, École Polytechnique Fédérale de Lausanne, Lausanne, Switzerland; 3 QuantumGreen, San Francisco, CA USA

**Keywords:** Cheminformatics, Computational methods

## Abstract

From reaction optimization to molecular design, experimental discovery poses the same expensive question: which candidate to test next under time and resource constraints. Bayesian optimization provides principled answers but depends on domain expertise that rarely transfers. Large language models (LLMs) contain rich scientific knowledge but lack the calibrated uncertainty estimates crucial for high-stakes decisions. Here we show how training language models through Bayesian objectives enables their use as reliable optimizers guided by natural language. Our approach, GOLLuM (Gaussian process Optimized LLMs), teaches LLMs from experimental outcomes under uncertainty, transforming their overconfidence from a fundamental flaw into a precise learning signal. This signal reshapes the LLM embeddings so that experiments with similar outcomes cluster together, revealing structure in the design space. Starting from only ten low-performing experiments, GOLLuM generalizes across 23 tasks in organic synthesis, materials science, process chemistry and molecular design, ranking first on average among all competing methods. It matches traditional Bayesian optimization with over 40% fewer experiments and nearly doubles the discovery of high-performing Buchwald–Hartwig reactions over expert quantum-chemical descriptors and state-of-the-art LLMs (43% versus 24–25%). More broadly, GOLLuM points to a different paradigm for specializing foundation models: not through more data but through richer, uncertainty-guided information.

## Abstract (web summary)

Although language models can be helpful in molecular design, they are not typically calibrated for uncertainty. Rankovic and colleagues present a method to train language models while taking into account the uncertainty of the data.

## Main

Artificial intelligence (AI) can now generate millions of novel drug candidates, material structures or chemical reactions in a matter of hours, expanding an already vast combinatorial landscape that has long challenged experimental science^1–3^. However, the fundamental bottleneck remains: deciding which candidates to evaluate next. This explosion of possibilities demands systematic, sample-efficient approaches to navigate vast design spaces under time and resource constraints^4–7^.

Established experimental optimization methods, such as Bayesian optimization (BO)^8–14^, address this problem through a probabilistic lens. BO relies on surrogate models, typically Gaussian processes (GPs)^15^, to provide experimental predictions together with uncertainties, balancing exploration and exploitation^16–18^. In practice, however, BO typically depends on domain-specific feature representations^19–22^. Optimizing a chemical reaction, for example, requires first translating molecular structures, catalytic mechanisms and reaction pathways into descriptors that encode chemical intuition^23,24^. These representations rarely transfer: reaction fingerprints cannot characterize crystal structures, catalyst optimization bears little resemblance to drug design, and even within catalysis, descriptors optimized for a metal-ligand system may not be effectively transferred to others^25–27^. Although BO provides a general approach, each new domain effectively starts from scratch with little performance guarantee, creating barriers to adoption and limiting the transferability of expertise^28^.

By contrast, large language models (LLMs) have emerged as generalist models that transcend domain boundaries through their rich prior knowledge^29–32^. The potential use of LLMs for experimental optimization has attracted considerable interest, yet adoption remains constrained by a fundamental flaw: unreliability. LLMs hallucinate, producing outputs that sound confident but are factually incorrect^33–35^. Although this limitation frustrates users in everyday applications, it can have severe consequences in experimental science. Hallucinated experimental suggestions waste resources, delay research timelines and compromise safety protocols in automated laboratory settings^36,37^. Unsurprisingly, their integration into high-stakes experimental workflows remains impractical without rigorous control^38,39^.

The merging of the two paradigms, BO and LLMs, has therefore gained substantial traction^40–45^. Yet existing approaches treat language models and probabilistic optimization separately. Here, we introduce GOLLuM (Gaussian Process Optimized LLMs) as the first framework to train language models through the same probabilistic objective that makes BO reliable. Rather than positioning LLMs as direct experimental optimizers, where they struggle with hallucination and heuristic search, we demonstrate how their probabilistic integration within established optimization mechanisms enables principled decision-making from natural language.

**Fig. 1:**
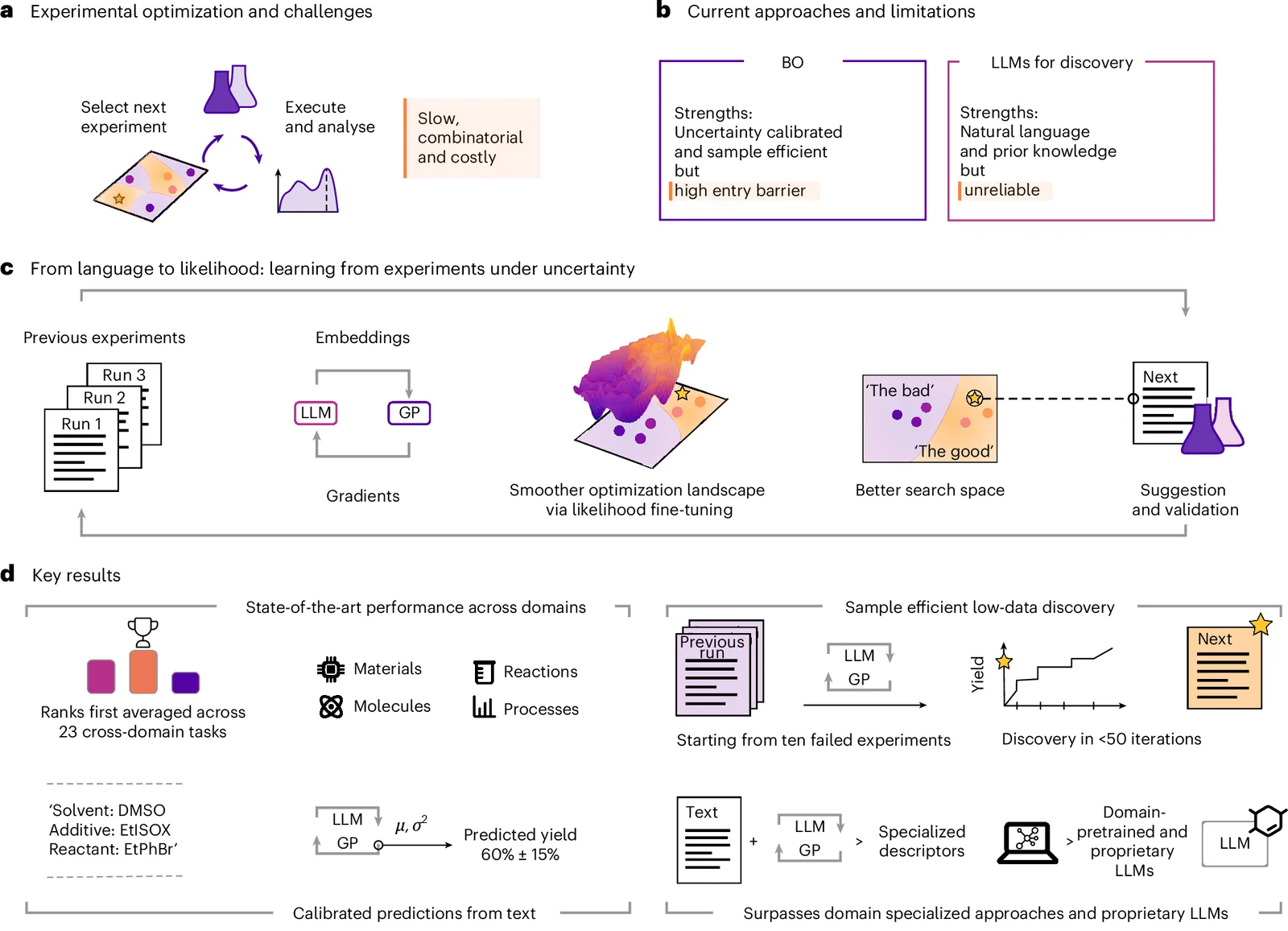
Language models as uncertainty-calibrated optimizers. **a**, Experimental optimization challenges. Scientists face the universal problem of efficiently exploring vast design spaces under time and cost constraints. **b**, Current approaches impose a trade-off between accessibility and reliability. BO provides principled uncertainty calibration and sample efficiency but requires domain expertise. LLMs offer natural language interfaces and prior knowledge but lack reliability. **c**, GOLLuM optimization loop. Previous experimental results, described in natural language, feed into joint optimization of both GP and LLM components. Likelihood gradients from the GP fine-tune the LLM, progressively smoothing the optimization landscape and organizing the search space into two distinct regions of high and low performance: the good and the bad, respectively. This structured representation enables easier selection for the next experiment, which becomes a new training point that continues the loop. **d**, Key results. GOLLuM ranks first averaged across 23 cross-domain tasks spanning materials, reactions, molecules and processes, enabling sample-efficient discovery from natural language inputs while surpassing specialized descriptors, domain-pretrained models and state-of-the-art LLMs.

GOLLuM trains the LLM jointly with the GP through its marginal likelihood (Fig. 1). Gradients from this objective teach the LLM to encode experimental outcomes rather than textual similarity, organizing its latent space into distinct regions of high and low performance. This learned organization reveals structure in the experimental landscape while providing interpretability across scientific domains. In Buchwald–Hartwig cross-coupling reactions, the transformed representations expose genuine chemical patterns, automatically clustering iodide-based reagents in high-yield regions while separating low-performing chlorides. Crucially, this interpretability extends beyond organic synthesis: in analytic and process chemistry, the model identifies optimal ether-alcohol solvent systems; in materials science, it isolates the high-performing Mn–Na–W region of the catalyst space; and in molecular design, it recovers structure-property trends for photoswitches, all without explicit domain instruction.

By exploiting these discovered patterns, GOLLuM achieves state-of-the-art performance. In the Buchwald–Hartwig benchmark, it nearly doubles the discovery rate of high-performing conditions compared with static embeddings, physics-based descriptors and state-of-the-art LLMs. These improvements extend to other domains, from materials synthesis to molecular design, with the largest margins in process chemistry, a 90% relative improvement over traditional BO. Across 23 diverse benchmarks covering combinatorial, mixed-variable and continuous optimization, our approach ranks first on average while using fixed hyperparameters and starting from ten failed experiments (below the median), outperforming both domain-specialized descriptors and state-of-the-art LLMs.

These results suggest that uncertainty, rather than being LLMs’ fatal flaw for experimental science, can drive their transformation into reliable optimizers. By training language models through GP objectives, we combine the accessibility of natural language with the reliability required for real experimental campaigns. This principle of uncertainty-driven adaptation suggests a broader paradigm for deploying AI in high-stakes domains where calibrated confidence matters as much as capability.

## Robust and efficient optimization across scientific domains

The central premise of language model-based optimization is the potential for a single framework to leverage prior knowledge across diverse scientific problems without domain-specific feature engineering. Traditional optimization approaches often rely on representations tailored to each domain (for example, molecular and reaction fingerprints or quantum mechanical descriptors). However, no single descriptor family is universally appropriate across the heterogeneous domains in experimental science, and practical performance often depends on task-specific choices (for example, which fingerprint/graph/quantum mechanical features, how to encode conditions, what granularity of reaction representation is available). Instead, we analyse whether the expressive power of language models under probabilistic alignment can capture the underlying objective-relevant patterns from textual descriptions, thereby reducing the need for specialized representations by navigating the optimization from text.

**Fig. 2:**
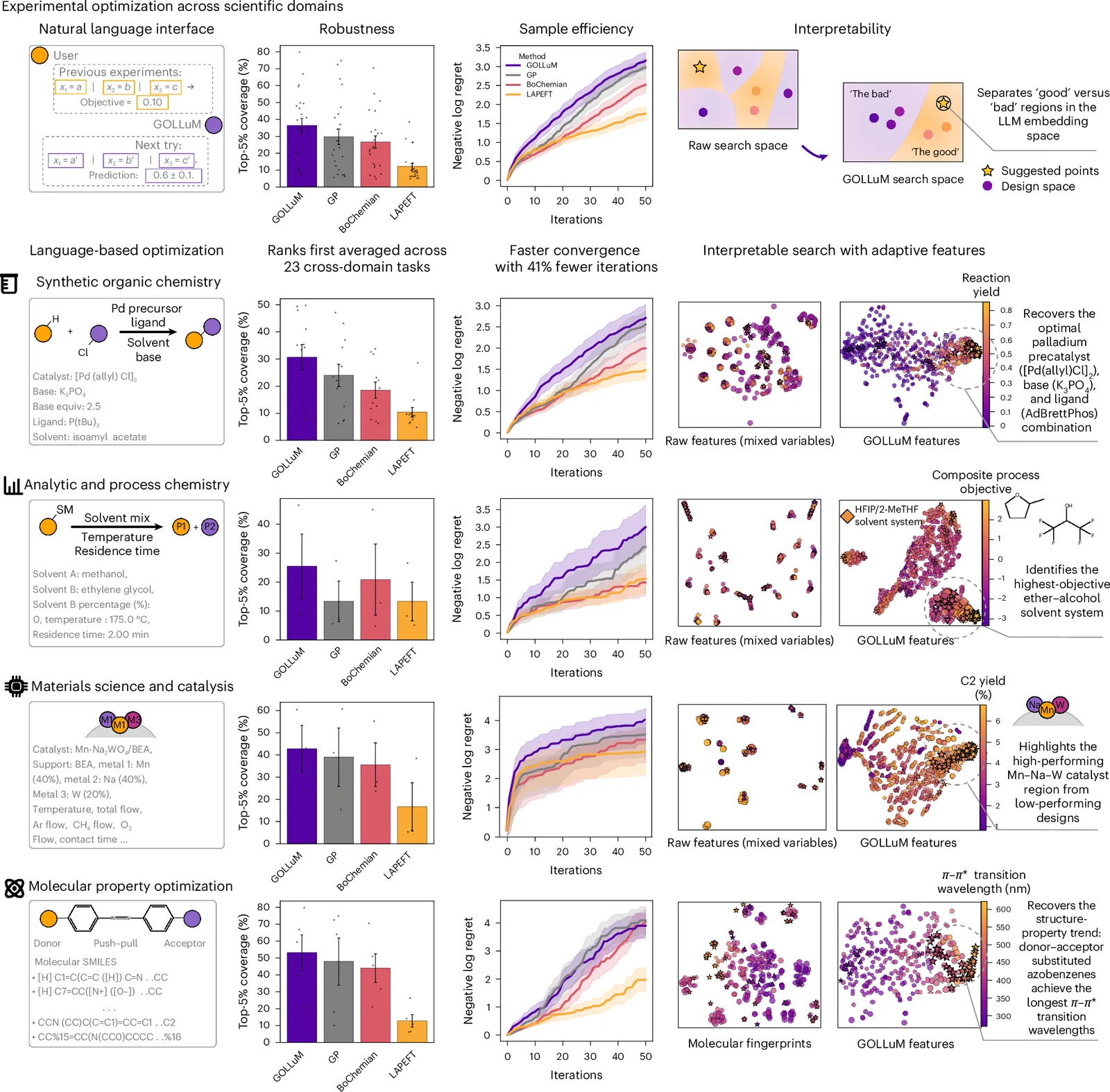
Experimental optimization across scientific domains. GOLLuM unifies a GP surrogate with a language encoder trained through the GP marginal likelihood, enabling (1) robust optimization across 23 chemistry/materials tasks (top-5% coverage), (2) faster discovery under equal experimental budget (50 experiments; PLLM*ϕ* as main GOLLuM variant) and (3) interpretable organization of the design space where high-performing regions become separable. Rows show representative domains (synthetic organic chemistry, analytic/process chemistry, materials/catalysis and molecular property optimization). Sample-efficiency curves show the mean standardized negative log regret with 95% confidence intervals across 20 independent seeds per benchmark, starting from 10 initial observations. Bar charts display the mean top-5% coverage across the benchmarks in each domain; the overlaid dots show the per-benchmark means (one point per benchmark) and error bars the standard error of the mean across benchmarks. Interpretability panels show a two-dimensional t-distributed stochastic neighbour embedding projection of the LLM latent space, with points coloured by their objective value, ranging from low (dark/purple) to high (light/yellow) performance. All LLM-based methods use T5^57^ as the encoder. Source data

To evaluate this capability, we benchmark GOLLuM across 23 optimization tasks spanning organic synthesis, materials science, process chemistry and molecular design (Fig. 2). These tasks include combinatorial search over discrete molecular spaces, continuous optimization of process conditions and mixed-variable problems combining discrete and continuous parameters. Importantly, we use a single set of hyperparameters and do not introduce task-specific tuning. The full suite comprises four chemistry domains: organic chemistry (12 tasks): five Buchwald–Hartwig reactions, four Ni-catalysed additive screens, Suzuki–Miyaura coupling, Pd-catalysed sulfonamide coupling and reductive amination; analytic and process chemistry (3 tasks): an allyl-catechol rearrangement, HPLC method optimization and Buckminsterfullerene C_60_ adduct synthesis; materials and catalysis (3 tasks): C2-yield catalysis for methane oxidative coupling, oxygen-evolution reaction catalysts and vapour-diffusion crystallization; and molecular property optimization (5 tasks): redox potential of redox-flow-battery molecules, aqueous solvation energy, docking score against a kinase target, the *π*–π^*^ transition wavelength of organic photoswitches and power-conversion efficiency of organic photovoltaic candidates. Per task objectives, dataset sizes and sources are described in detail in Supplementary Information.

We report negative log regret curves, alongside the top-5% coverage, which measures how many evaluated experiments fall within the top-5% of outcomes. We motivate this metric with the practical need to identify multiple near-optimal conditions that can satisfy secondary constraints (for example, cost^46^, sustainability^47^, safety^48^ or reagent availability^49^).

**Fig. 3:**
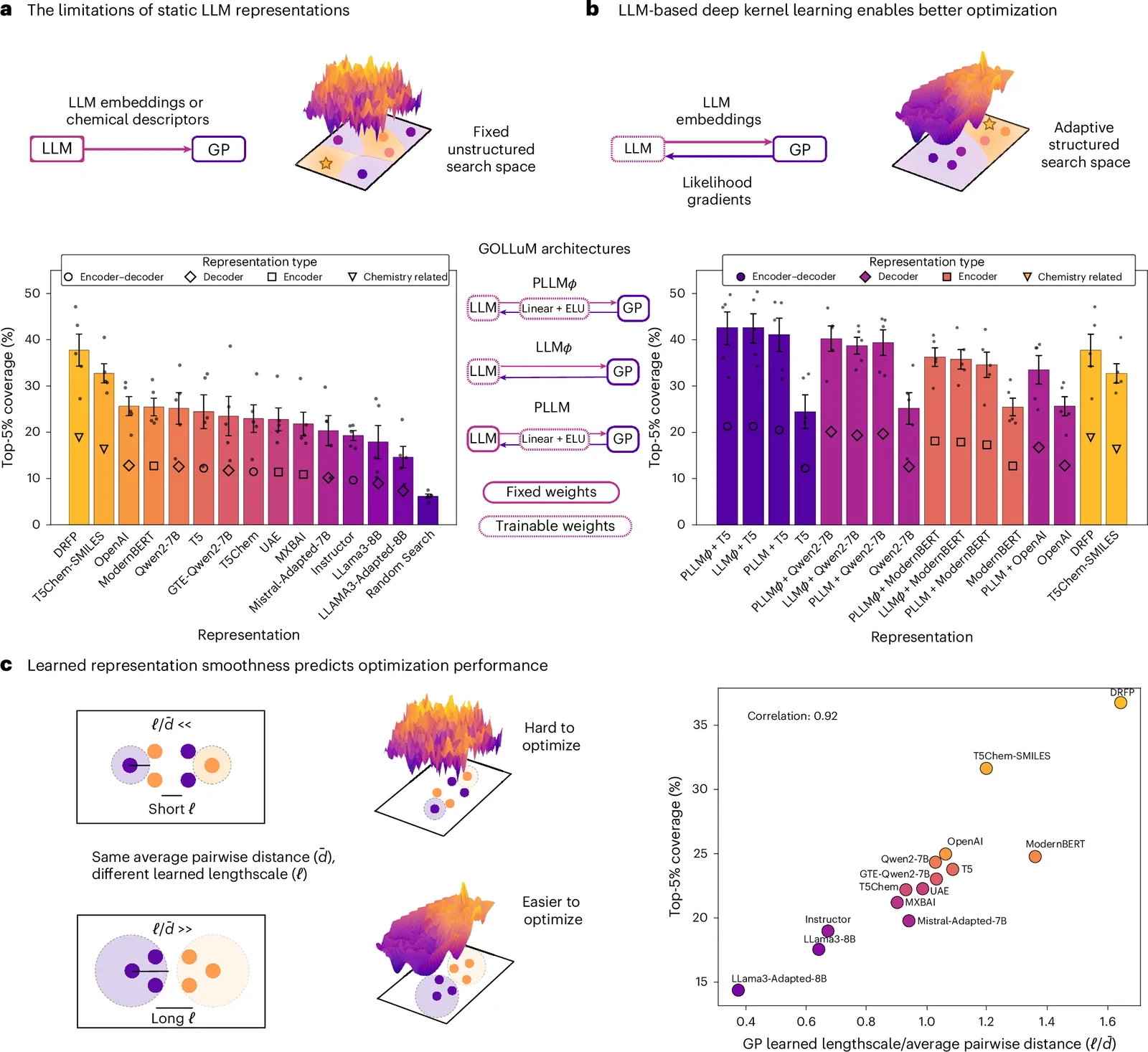
Representation geometry enables efficient BO. **a**, BO performance with fixed LLM features as input to GP on Buchwald–Hartwig tasks. We show average discovery of high-impact regions of the design space as the percentage of the top-5% reactions found during the optimization, aggregated across all five Buchwald–Hartwig reactions. Chemistry-related representations include T5Chem-SMILES^53^, a pretrained chemistry-related LLM with SMILES input, and DRFP^51^, a reaction fingerprint. **b**, Comparative analysis of GP-based LLM fine-tuning. The fine-tuned models are arranged by the overall performance and relative improvements to their base (fixed embeddings) LLM-GP variants. Chemistry-related baselines (previous best) included for comparison. All results show mean and standard error across 20 independent seeds per model for all five Buchwald–Hartwig reactions, with each optimization run starting from 10 initial points (random below median) and selecting subsequent points via acquisition function maximization over the enumerated design space. Overlaid dots in **a** and **b** represent the per-Buchwald–Hartwig-reaction mean coverage. **c**, Data representations and their success rates in BO. BO performance correlates with GP smoothness, measured as the ratio of learned lengthscale to average pairwise embedding distance. Higher ratios indicate smoother GP fits, implying the representation supports meaningful generalization across the design space. Source data

For clarity, we evaluate three GOLLuM variants: PLLM, LLM*ϕ* and PLLM*ϕ*. PLLM learns a linear projection on top of fixed LLM embeddings while *ϕ* variants update the LLM parameters. We use T5 as a general-purpose pretrained LLM encoder. For all GOLLuM variants, we optimize the trainable parameters via GP marginal likelihood. An architectural summary is provided in Fig. 3.

Across the 23 problems, GOLLuM (PLLM*ϕ* + T5) ranks first on average, achieving 36.3% top-5% coverage at the 50-experiment budget from a cold-start scenario (ten initial low-performing experiments) while outperforming other LLM-BO baselines (BoChemian, 26.5%; LAPEFT, 12.1%) (Fig. 2). The gains over BoChemian, which uses fixed LLM embeddings as inputs to a GP surrogate, support the benefit of optimization-aware adaptation over static representations.

The comparison with LAPEFT further highlights the importance of integrating uncertainty into training rather than treating it as a post hoc add-on. LAPEFT adapts the encoder using a supervised regression loss (for example, MSE) and applies post hoc uncertainty estimation via a Laplace approximation, whereas GOLLuM trains the LLM through the GP marginal likelihood itself. This coupling jointly improves predictive fit and calibrated uncertainty, which is essential for reliable sequential and batch decision-making. Despite the advanced LLM-BO baselines, the closest overall competitor remains a standard BO using a GP surrogate with domain-tailored representations (29.7%).

Performance also exhibits clear domain structure. The largest gains occur in process chemistry, where GOLLuM improves coverage from 13.4% (GP) to 25.4% top-5% with a 90% relative improvement. In organic chemistry, coverage increases from 23.9% to 30.6% (+28%) while in materials/catalysis, improvements are more modest but consistent (for example, 39.0–42.7%). In molecular property optimization tasks, where established molecular fingerprints already provide strong signal for many objectives, GOLLuM remains competitive (47.9–53.2%). This pattern becomes clearer when we group benchmarks by feature availability. Tasks relying on specialized molecular or reaction fingerprints show modest gains (32.4–37.3%, +15%), because these features may already provide structured latent spaces (Fig. 2 and Supplementary Fig. 15). By contrast, mixed-variable tasks where no such specialized features exist show substantially larger improvements (24.9–35.8%, +44%), as do numerical-only tasks (24.6–33.3%, +35%). There, GOLLuM learns structure that fixed representations do not capture.

Beyond final coverage, GOLLuM is the only LLM-based method in our comparison to improve sample efficiency over traditional BO (Fig. 2, ‘sample efficiency’). It reaches high-performing regions earlier, matching the final performance of traditional BO (GP) with a median of 41% fewer iterations before surpassing it.

Finally, outside of performance and speed, GOLLuM yields interpretable search behaviour by organizing the design space into outcome-aligned regions (Fig. 2, ‘interpretability’). In synthetic organic chemistry, it recovers the optimal reaction conditions and groups them into a coherent chemical family; in analytic and process chemistry, it identifies a high-performing solvent system; in materials and catalysis, it highlights the optimal Mn–Na–W region in the catalyst space; and in molecular design, it recovers meaningful structure-property trends for photoswitches. Together, these results indicate that probabilistic alignment supports not only sample-efficient optimization but also human-interpretable organization of complex experimental design spaces.

## Static LLM embeddings underperform as chemical descriptors

Having established GOLLuM’s cross-domain performance, we now turn to a controlled diagnostic on the fully enumerated Buchwald–Hartwig C–N cross-coupling benchmark^50^ to analyse the mechanism governing this success.

To understand why GOLLuM works, we first analyse where its closely related LLM-BO baselines struggle. A representative example is BoChemian, which performs BO with a GP surrogate over fixed LLM embeddings. Although convenient, fixed embeddings do not necessarily encode the right level of information about the underlying objective. A critical requirement for scientific optimization, however, is a representation that successfully captures the relationship between experimental parameters and their outcomes^22^.

We further investigate standard GP baselines with specialized features (reaction fingerprints DRFP^51^) and fixed LLM embeddings across a variety of LLM architectures. We observe that static LLM representations consistently underperform specialized chemical descriptors (Fig. 3a). In 50 optimization trials, LLM embeddings helped discover only 15–26% of the highest-yielding reactions (fifth percentile threshold), trailing behind the 38% achieved by specialized chemical fingerprints (DRFP^51^). OpenAI’s embeddings^52^, while reaching the highest score among the LLM embeddings, still failed to close this performance gap.

The simple lack of chemical knowledge does not explain these differences. Chemistry-specialized language models, such as T5Chem^53^, underperform general-purpose variants, achieving a 23% discovery rate despite extensive pretraining on chemical tasks. The model’s dependence on input format reveals underlying patterns that explain the disparity in results. Changing T5Chem’s input from an experimental procedure to the Simplified Molecular Input Line Entry System (SMILES) format^54–56^ improved its discovery rate by 44%. This improvement is not surprising given the model’s inherent familiarity with the SMILES notation from its pretraining. Such format dependency indicates the core issue is not the knowledge the model contains but rather its ability to translate that knowledge into the embedding space.

These results highlight the limitations of static LLM embeddings for experimental guidance. Without direct adaptation to the optimization task, they force a trade-off between the convenience of natural language and the optimization performance of structured descriptors. We next show that this failure mode is reflected in the induced GP geometry: representations that yield smoother GP fits support substantially better optimization.

## Representation geometry determines optimization success

The representation geometry determines optimization success better than the predictive accuracy of a surrogate model (Fig. 3c). Although regression metrics such as *R*^2^ explain how well the surrogate fits the objective function, they do not offer a clear answer as to what dictates the optimization performance.

The ratio of the GP’s learned lengthscale (*ℓ*) to the average pairwise distance between points in the search space ($\overline{d}$) robustly predicts optimization success. The lengthscale defines the distance in the input space over which the model expects output values to be correlated. A higher $\ell /\overline{d}$ indicates a smoother, more GP-friendly embedding space where the model can generalize over a larger area because the embeddings position similar outcome experiments together. Across all 14 representations, this geometric measure correlates strongly with BO performance (*r* = 0.92 versus *R*^2^:*r* = 0.78, weighted *R*^2^:*r* = 0.82; Supplementary Fig. 5). By contrast, representations with a low smoothness ratio create rough, difficult-to-model objective landscapes with potentially many local minima.

These findings underscore a well-structured representation as a more important factor for successful optimization than the raw predictive accuracy itself. If a GP surrogate with a stationary kernel (where the relationship between two points depends only on their distance) assumes a smooth objective function, the underlying representation should support it. Specialized chemical descriptors (DRFP^51^) instill some organization in the latent space, resulting in a higher ratio ($\ell /\overline{d}\approx 1.6$) and better performance, while the static LLM embeddings exhibit low ratios, resulting in rough, difficult-to-model objective landscapes. The failure of static LLM embeddings is therefore a failure of alignment between the representation’s geometry and the surrogate model’s requirements, motivating a method that can actively learn to resolve this incompatibility.

## Uncertainty-guided LLM fine-tuning enables efficient optimization

To resolve the misalignment between the representation and the surrogate, we train the LLM jointly with the GP through its marginal likelihood. This approach transforms the LLM from a static feature extractor into a dynamic one, adapting its pretrained knowledge to the optimization task.

In the same Buchwald–Hartwig reactions benchmark as above, this joint GP-LLM training substantially improved optimization efficiency (Fig. 3b). An encoder-decoder Google T5^57^ model increased the discovery rate of high-yielding conditions from 24% to 43%, with a 79% relative improvement compared with its static representation. This increase surpassed specialized fingerprints, achieving the new state-of-the-art.

Similar improvements held across other architecture types. The same approach elevated decoder-only models (61% relative improvement for Qwen-7B^58^), encoder-only models (42% for ModernBERT^59^) and raised the score of closed-source OpenAI^52^ embeddings (by 31%) without access to the base model’s weights by incorporating a learned projection on fixed embeddings (PLLM). Moreover, this variant offers a strong low-compute alternative when model access is constrained while the *ϕ*-fine-tuning provides an additional advantage by changing the representation itself, under a low-rank/pretrained-anchored constraint.

These systematic improvements across major LLM architecture types suggest a general principle rather than an isolated finding: that task-specific adaptation is more important than domain-specific pretraining. Indeed, general-purpose LLMs with no chemistry-specific pretraining (T5^57^ and Qwen-7B^58^) surpassed both chemistry-specialized features (DRFP^51^) and specialized chemistry models (T5Chem^53^).

The mechanism behind the sample-efficiency lies in the training objective. By fine-tuning the LLM with the GP’s marginal likelihood, we merge both components under a shared probabilistic objective that inherently balances data fit with uncertainty estimates. This training paradigm repurposes the uncertainty into an LLM learning signal that guides the reorganization of its latent space. Traditional LLM fine-tuning has often required thousands of training examples. Here, we instead fine-tune large pretrained language models with ten initial points (adding one example per iteration in 50 sequential trials) adapting their latent spaces into smooth and calibrated representations for the optimization task.

These results indicate that uncertainty-driven fine-tuning can unlock task-specific effectiveness from generic LLMs, bypassing the need for specialized model training in each new domain.

## Emergent chemical organization supports interpretable optimization

Beyond improved performance, the fine-tuned LLM’s latent space exhibits emergent organization of chemical knowledge. Although Fig. 2 showed these structures emerging across domains, this section explains their mechanism.

**Fig. 4:**
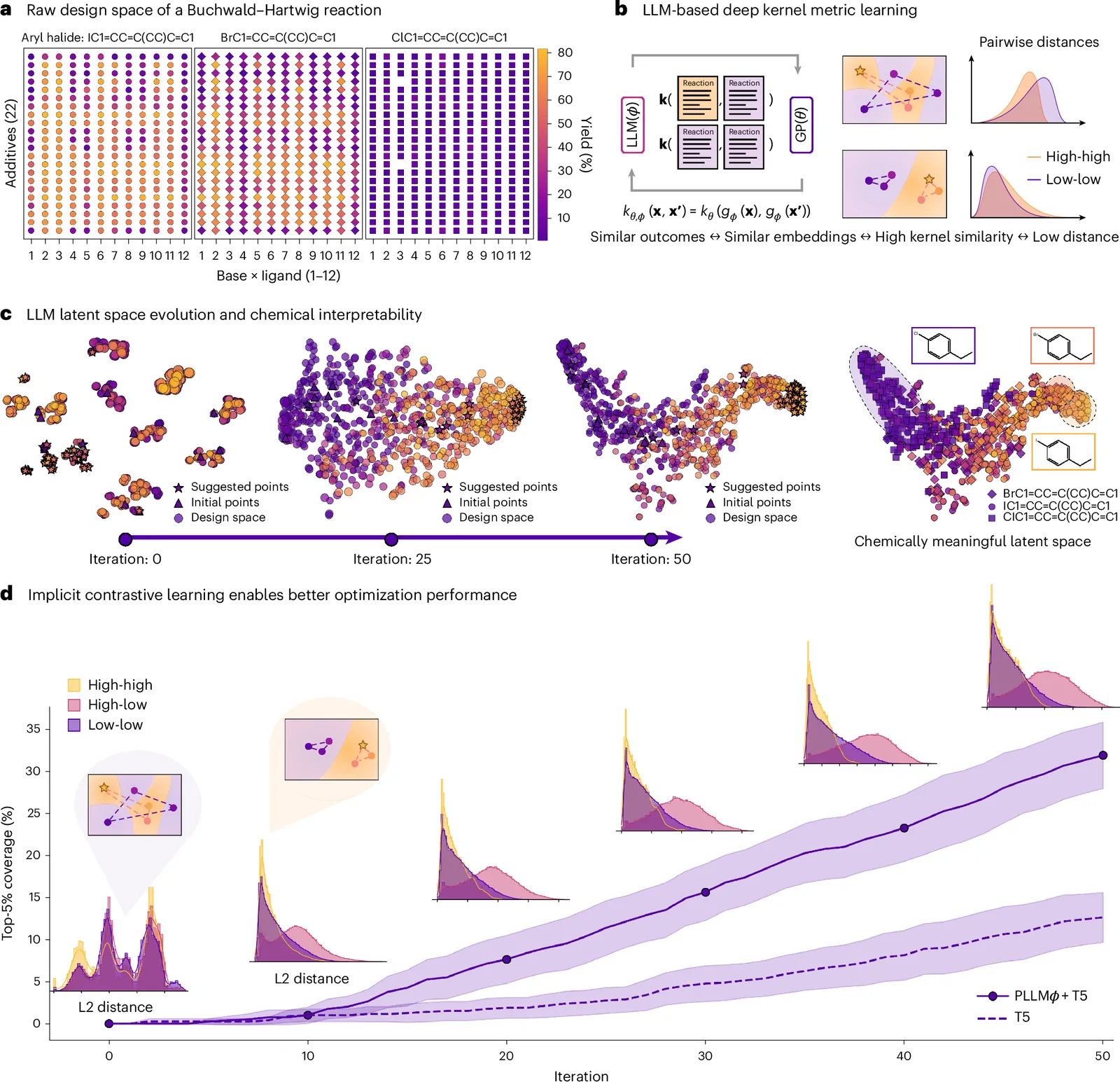
Implicit contrastive learning and emergent chemical interpretability with LLM-based deep kernel GPs. **a**, The raw design space of one of the Buchwald–Hartwig reactions, showing reaction yields across different combinations of additives, bases and ligands for three different aryl halides (I, Br and Cl). **b**, A schematic of LLM-based deep kernel metric learning. The joint optimization of the LLM and GP encourages the model to learn embeddings where experiments with similar outcomes position close together in the latent space, resulting in high kernel similarity and low pairwise distance. **c**, The evolution of the LLM’s latent space during the optimization process, shown as a two-dimensional t-distributed stochastic neighbour embedding projection. Starting from an unstructured state (iteration 0), the space becomes progressively more organized (iteration 25), eventually forming a chemically meaningful map where reactions cluster based on key reactivity patterns and performance (iteration 50). **d**, The link between implicit contrastive learning and optimization performance. The main plot shows that the adaptive model (PLLM*ϕ* + T5) significantly outperforms the static model (T5); lines show the mean best-so-far across 20 independent seeds and shaded bands the standard error of the mean over seeds. The inset histograms show the distribution of pairwise L2 distances between high-yielding and low-yielding experiments at different iterations. As the optimization progresses, the model learns to separate high- and low-performing points, creating a more structured space that enables more efficient discovery. Source data

A persistent challenge limiting the adoption of AI in experimental science is the ‘black-box’ problem: even if the model’s suggestions are effective, they often lack a clear scientific rationale. Our framework addresses this constraint by making the learning process transparent. In the Buchwald–Hartwig task, we observe that the organization is not immediate but emerges progressively (Fig. 4c). By the tenth optimization iteration, the model begins to cluster experiments according to key chemical features and their outcomes. For instance, experiments using high-reactivity iodide-based aryl halides cluster in a region associated with high yields, while those using less reactive chlorides occupy a different, lower-performance region.

This evolution confirms that the GP marginal likelihood objective acts as an implicit contrastive loss, actively separating successful and unsuccessful experiments over time (Fig. 4d). The resulting data-driven grouping mirrors well-known reactivity trends in cross-coupling chemistry, despite the model never being explicitly taught these relationships.

This emergent structure is also functionally useful. By grouping successful experiments and separating failures, the model gives the GP a smoother landscape that enables more sample-efficient exploration. Beyond efficiency, the organized space makes the model’s reasoning legible. A proposal’s position relative to known high- and low-performing regions provides a direct rationale for why it is likely to succeed. Because this rationale is available before an experiment is run, the optimizer’s suggestions can be inspected and questioned rather than merely followed.

## LLMs can be unreliable as direct optimizers

Finally, to verify the necessity of our probabilistic framework, we examine the reliability of using out-of-the-box LLMs as direct optimizers. Using LLMs as direct, prompt-based optimizers is an appealing strategy for leveraging their vast prior knowledge in scientific discovery^41,60–62^. In such scenarios, the LLM directly proposes experimental conditions based on the prompt containing details of the design space, previous experiments and the optimization goal. However, this application relies on the model’s black-box patterns and ignores known limitations, including overconfidence and a lack of principled uncertainty calibration.

**Fig. 5:**
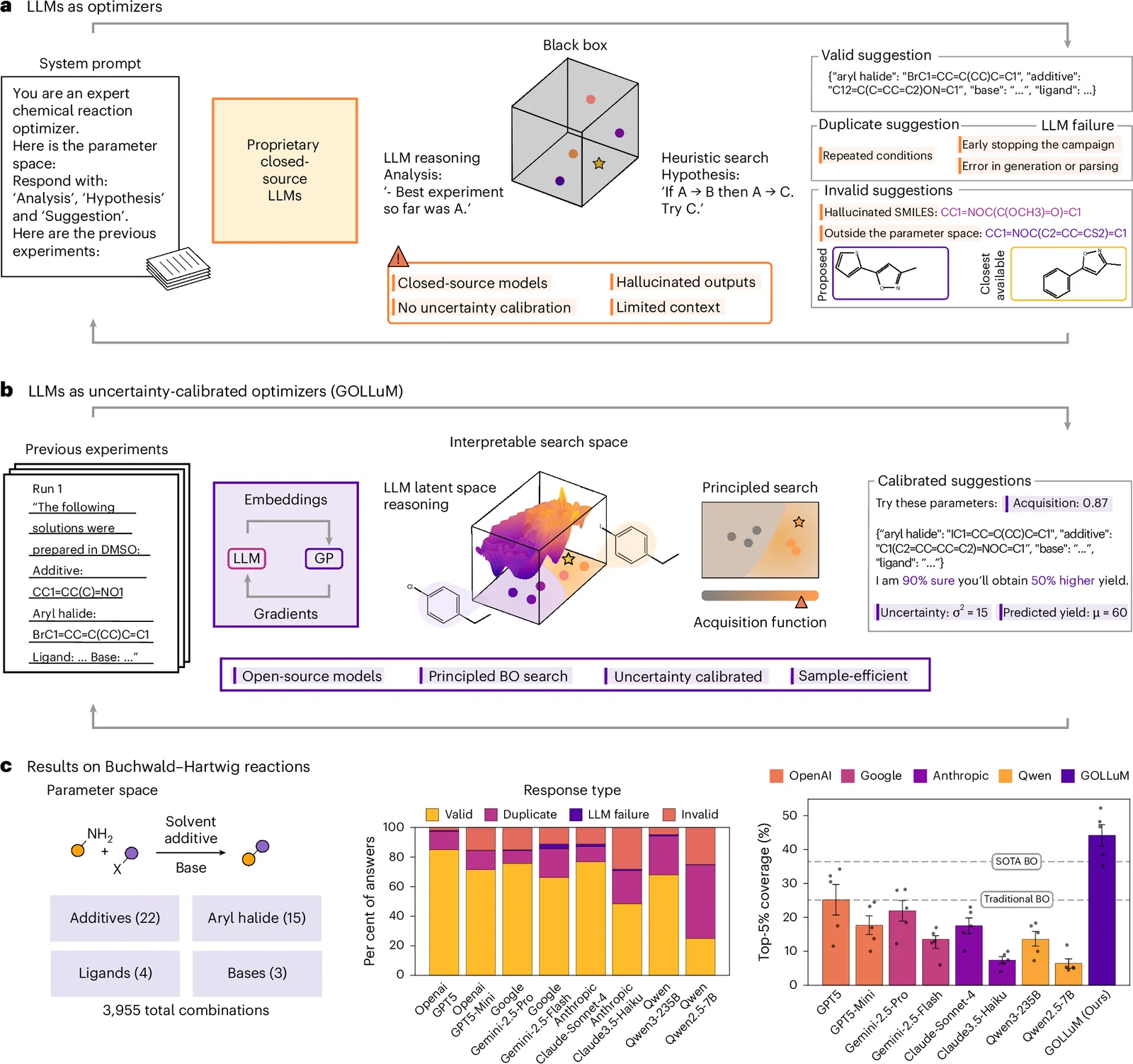
From LLM prompting to LLMs as principled scientific optimizers. **a**, Querying LLMs for optimization. Given the parameter space and previous experiments, the LLM proposes next suggestions. This method relies on heuristic search and suffers from a lack of uncertainty calibration, which leads to invalid suggestions, hallucinations and frequent failures. **b**, GOLLuM framework integrates an LLM and a GP in a joint optimization loop. Gradients from the GP’s probabilistic objective fine-tune the LLM, creating an interpretable search space that enables principled, uncertainty-aware and sample-efficient optimization. **c**, Quantitative results on the Buchwald–Hartwig reactions benchmark. Left: the combinatorial design space of 3,955 reactions across five distinct chemical products, corresponding to ~800 unique conditions per product. Middle: response-type analysis across several state-of-the-art LLMs reveals high failure rates, with a large fraction of invalid or duplicated suggestions. Responses are categorized as valid, duplicate (repeated within a run), invalid (outside the parameter space, for example, hallucinated reactants) or LLM failure (unparsable output or timeout); the full querying protocol is given in the Supplementary Information section ‘LLMs as direct optimizers’. Right: a comparison of optimization performance (‘top-5% coverage’) shows that direct LLM approaches substantially underperform both traditional BO (with density functional theory descriptors^20^) and state-of-the-art BO (with DRFP reaction fingerprints^51^), as well as our proposed GOLLuM method using GP-fine-tuned general-purpose open-source LLMs (T5^57^). Bars show the mean across the five Buchwald–Hartwig products; overlaid dots are the per-product mean coverages (*n* = 5 products) and error bars the standard error of the mean over products. Source data

We evaluate this approach by prompting several state-of-the-art LLMs (proprietary and open-weight) to act as expert chemical optimizers on Buchwald–Hartwig^50^ reactions, provided with the parameter space and a history of previous experiments. A quantitative analysis revealed high failure rates across all tested models (10% to ~80% in Fig. 5c, ‘response type’). A significant fraction of suggestions were invalid due to hallucinated chemical structures or proposals outside the defined parameter space. Furthermore, models frequently suggested duplicate conditions, failed to generate parsable outputs or tried to prematurely end the optimization campaign.

Although LLMs offer integrated reasoning and contextual analysis of the optimization problem, there are currently no clear mechanisms to evaluate or mitigate their overconfidence or hallucinations in a quantifiable manner. Moreover, when benchmarked against specialized BO approaches, direct prompting underperformed, trailing both standard GP methods and GOLLuM (Fig. 5c, top-5% coverage). This result highlights the value of integrating LLM knowledge within a probabilistic framework rather than relying on prompting alone.

Together, these results show that, although LLMs offer valuable reasoning and domain knowledge, they lack the calibration needed for autonomous experimental decisions. Rather than treating this gap as a fundamental limitation, we show how to resolve it through probabilistic integration.

## Discussion

In this work, we resolve the critical trade-off between the domain knowledge of LLMs and the principled reliability of BO. By repurposing the uncertainty from a GP as a direct training signal, we transform a generalist language model into a sample-efficient, domain-aware scientific optimizer. This transformation enables what neither paradigm could achieve alone: the generality of a text-based interface with the reliability required for experimental campaigns.

Our empirical evaluations demonstrate that fine-tuning language models with GP uncertainty consistently matches or outperforms specialized, expert-engineered features in guiding scientific optimization. This robust mechanism allows the framework to generalize effectively across 23 disparate benchmarks without task-specific modifications. Such cross-domain success stems from an emergent understanding of the underlying optimization landscape, a principle quantified by the strong correlation between our geometric smoothness metric ($\ell /\overline{d}$) and search efficiency. These properties make the framework a powerful tool for accelerating discovery in self-driving laboratories and human–AI collaborative experiments.

Although our approach demonstrates broad applicability across the chemical sciences, we acknowledge several limitations that open avenues for future research. The cubic scaling of standard GPs may pose a challenge for optimization campaigns with thousands of experiments, motivating the integration of scalable GP approximations^63,64^. Furthermore, although text provides a powerful universal interface, its effectiveness may be reduced in domains dominated by complex, high-dimensional data lacking a clear linguistic structure, such as protein conformations or crystal structures. Future work could explore hybrid models that combine our framework with specialized encoders for such data types.

This work suggests that a path to more capable and reliable LLMs for science lies in grounding them in principled probabilistic reasoning. Our findings reframe uncertainty from a flaw to be mitigated into a rich training signal, one that shifts the models’ learning goal from making the best predictions to providing well-calibrated ones. The ability to optimize a chemical reaction, a material synthesis protocol or a molecular design through natural language lowers the barrier to entry for researchers across scientific disciplines. This reframing points to a different paradigm for achieving specialization: not through more data but through richer, uncertainty-guided information.

## Methods

### BO overview

BO is a sample-efficient method for optimizing black-box functions, potentially expensive to evaluate. Such a setting is common in chemistry, where each experiment incurs substantial costs. BO works by training a probabilistic surrogate model (typically a GP) on previously observed data and using it to select new, informative queries via an acquisition function. We provide a detailed technical overview in Supplementary Section 2. The effectiveness of BO depends on the choice of representations and the quality of the surrogate model—both of which we improve in this work.

### Data representation

BO performance in chemistry is highly sensitive to the choice of data representation, especially given the heterogeneous data types (for example, categorical reagents, numeric conditions and molecular structures), combinatorial design spaces and variable numbers of parameters involved—making representation a critical challenge^22^. Natural language offers a flexible medium for expressing such optimization problems as textual descriptions, whereas LLMs can transform these inputs—regardless of type—into unified continuous embeddings. We construct these embeddings through a two-step process:

1. Template construction: we define each optimization task *t* as a standardized template: *t* = template({parameters, values}), where values define the actual conditions of the problem (for example, reagents used in a chemical synthesis). For single-variable tasks (for example, molecular optimization), the template reduces to a single textual identifier such as a molecular SMILES string^55,56^. This approach provides a consistent format applicable across a wide range of optimization problems.
2. LLM embedding: we process the templated description through LLMs to obtain a fixed-dimensional embedding: ${\bf{x}}=\,\mathrm{LLM}(t)\in {{{{\mathbb{R}}}}}^{d}$. This embedding unifies heterogeneous parameter types and enables compatibility with standard continuous kernels (for example, Matérn), while preserving interparameter relationships and scaling to variable-length inputs.

The resulting embedding vector **x** captures both the individual parameter values and their interactions, providing a unified representation for subsequent GP modelling. This approach circumvents the need for designing specialized kernels, as the LLM embedding space naturally encodes meaningful distances—enabling optimization over mixed categorical and numerical inputs within a continuous space. Moreover, it generalizes to tasks with arbitrary combinations of categorical, numerical or structural parameters—making it broadly applicable beyond chemistry to domains where design spaces can be expressed through text.

### Gaussian process with fixed LLM embeddings

LLM embeddings can be directly used as input vectors to GPs, which model the output based on observed data. In this setup, the embeddings remain fixed throughout the optimization process—following the approach outlined in BoChemian^42^—and the model’s adaptability comes solely from learning the GP hyperparameters *θ*. We use a GP prior with a Matérn-5/2 kernel with trainable hyperparameters $\theta =\{\ell ,{\sigma }^{2},{\sigma }_{n}^{2},c\}$ representing the lengthscale, signal variance, observation noise variance and constant mean. This approach relies entirely on the pretrained LLM’s embedding space to define input structure—specifically, the relative positioning of points based on their underlying features. The GPs with stationary kernels (such as Matérn-5/2) assume this structure reflects meaningful relationships: points close together in the embedding space are expected to have similar outcomes. However, general pretrained LLMs may not reflect chemical similarities and their representations may not encode the right inductive biases for the task. As a result, the GP can struggle to model the objective effectively in the fixed-feature setting, unless the embedding space already captures relevant patterns. This limitation can be addressed through deep kernel methods, which we describe next.

### Deep kernel GP

Deep kernel GP^65^ combine the flexibility of deep neural networks with the principled uncertainty quantification of GP. In this approach, the kernel function is composed with a learned feature transformation

$${k}_{\theta ,\phi }({\bf{x}},{{\bf{x}}}^{{\prime} })={k}_{\theta }({g}_{\phi }({\bf{x}}),{g}_{\phi }({{\bf{x}}}^{{\prime} })),$$

where *g*_*ϕ*_ is a parameterized feature extractor with parameters *ϕ*. This composition allows the model to learn task-specific feature representations while maintaining the probabilistic properties of the GP framework. The learned transformation and the GP parameters are jointly optimized through the marginal likelihood where **K**_*θ*,*ϕ*_ is the kernel matrix computed using the transformed features.

### LLM-based deep kernel

In our framework, we explore different approaches to constructing the feature transformation *g*_*ϕ*_(⋅).

#### Projection layer

A learned transformation consisting of a linear projection ${\bf{P}}\in {{\mathbb{R}}}^{m\times d}$ followed by a nonlinear activation function (ELU), applied to fixed LLM embeddings: *g*_*ϕ*_(**x**) = **P**LLM(*t*) where *m* is the projection dimension. This setup closely follows standard deep kernel learning with a trainable transformation applied on top of fixed features before kernel evaluation. It is particularly useful in settings where LLM weights cannot be accessed, as in the case of closed-source models from OpenAI. The projection layer learns to emphasize or suppress different aspects of fixed LLM embeddings, effectively creating a task-specific representation.

#### PEFT-adapted LLM

Low-rank adaptation (LoRA) of LLM parameters includes *g*_*ϕ*_(**x**) = LLM*ϕ*(*t*), where *ϕ* represents the trainable adaptor parameters. Parameter efficient fine-tuning (PEFT)^66–68^ addresses the challenge of adapting LLMs by updating a smaller (often several orders of magnitude fewer) number of parameters, typically inserted into or alongside the LLM architecture. We use LoRA^67^ to preserve potential chemical knowledge captured during pretraining and learn task-specific adaptations, while avoiding catastrophic forgetting or compromising general capabilities.

#### Combined approach

Sequential application of LoRA and projection includes *g*_*ϕ*_(**x**) = **P**LLM*ϕ*(*t*), thus combining the benefits of both worlds. The LoRA adaptors allow the LLM to adapt its internal representations to the optimization task, while the projection layer provides an additional degree of freedom to reshape the embedding space.

With any of these methods, we optimize the parameters *ϕ* (projection matrix and/or LoRA parameters) jointly with the GP hyperparameters through the marginal likelihood. In other words, we are fine-tuning the LLM through the GP loss which allows the model to learn transformations that preserve relevant chemical information, organize the latent space to better reflect the structure of the optimization objective and provide well-calibrated uncertainty measures.

### LLM fine-tuning as GP marginal likelihood optimization

Let ${\mathcal{L}}(\theta ,\phi )$ denote the GP marginal likelihood of observing targets **y** given inputs **X**, LLM parameters *ϕ* and GP hyperparameters *θ*

1 $${\mathscr{ \mathcal L }}(\theta ,\phi )=\mathrm{log}p({\bf{y}}| {\bf{X}},\theta ,\phi )=-\frac{1}{2}\left({{\bf{y}}}^{\top }{{\bf{K}}}_{\theta ,\phi }^{-1}{\bf{y}}+\mathrm{log}| {{\bf{K}}}_{\theta ,\phi }| +n\mathrm{log}2{\rm\pi }\right).$$

To optimize the embedding parameters *ϕ* jointly with the GP hyperparameters *θ*, we maximize the marginal likelihood using gradient-based optimization

2 $${\theta }^{* },{\phi }^{* }=\arg {\mathop{\max }\limits_{\theta ,\phi }}{\mathcal{L}}(\theta ,\phi ).$$

We compute the gradients of the marginal likelihood with respect to the parameters via standard backpropagation

3 $${\nabla }_{\theta ,\phi }{\mathscr{ \mathcal L }}(\theta ,\phi )=\frac{1}{2}{{\bf{y}}}^{\top }{{\bf{K}}}_{\theta ,\phi }^{-1}\left({\nabla }_{\theta ,\phi }{{\bf{K}}}_{\theta ,\phi }\right){{\bf{K}}}_{\theta ,\phi }^{-1}{\bf{y}}-\frac{1}{2}\,\mathrm{Tr}\,\left({{\bf{K}}}_{\theta ,\phi }^{-1}{\nabla }_{\theta ,\phi }{{\bf{K}}}_{\theta ,\phi }\right).$$

We perform the joint optimization with separate learning rates for embedding parameters (*ϕ*) and GP hyperparameters (*θ*) to encourage stable convergence and avoid overfitting of either component.

### Implicit metric learning

The explicit feature of GPs to evaluate the similarities in the output based on the distances in the input space creates a contrastive learning effect for LLM embeddings. This beneficial consequence arises from the two-fold utilization of the GP marginal likelihood. The kernel function ${k}_{\theta ,\phi }({\bf{x}},{{\bf{x}}}^{^{\prime} })={k}_{\theta }({g}_{\phi }({\bf{x}}),{g}_{\phi }({{\bf{x}}}^{^{\prime} }))$ measures similarity between points, and optimizing the marginal likelihood (equation (1)) encourages embedding distances to decrease between points with similar outputs and increase between points with dissimilar outputs. The contrastive learning effect comes directly from the GP marginal likelihood optimization. For a kernel based on distances (such as Matérn) we can rewrite the term ${{\bf{y}}}^{\top }{{\bf{K}}}_{\theta ,\phi }^{-1}{\bf{y}}$ as a weighted sum of pairwise interactions (with weights *w*_*ij*_ defined by the inverse kernel matrix) inducing implicit contrastive learning objective ${{\mathcal{L}}}_{{\rm{implicit}}}$

4 $$\begin{array}{l}{{{ \mathcal L }}}_{\mathrm{implicit}}(\theta ,\phi )\propto \sum _{i,j}{w}_{ij}\parallel {g}_{\phi }({{\bf{x}}}_{i})-{g}_{\phi }({{\bf{x}}}_{j}){\parallel }_{2},\\\left\{\begin{array}{c}\parallel {g}_{\phi }({{\bf{x}}}_{i})-{g}_{\phi }({{\bf{x}}}_{j}){\parallel }_{2}{\downarrow }\,{\mathrm{if}}\,\,\parallel {y}_{i}-{y}_{j}\parallel \,\,\mathrm{is}\,\mathrm{small}\,\\ \parallel {g}_{\phi }({{\bf{x}}}_{i})-{g}_{\phi }({{\bf{x}}}_{j}){\parallel }_{2}{\uparrow }\,\mathrm{if}\,\,\parallel {y}_{i}-{y}_{j}\parallel \,\,\mathrm{is}\,\mathrm{large}\,\end{array}\right.\end{array}$$

In other words, the joint GP optimization induces high kernel values (small distances) between points with similar outputs and low kernel values (large distances) between points with different outputs, therefore separating the embedding space into distinct categories. This reorganization in the latent space happens automatically through the optimization of the deep kernel parameters, adapting the feature space to better align with outcomes without requiring explicit contrastive loss terms.

### Reporting summary

Further information on research design is available in the Nature Portfolio Reporting Summary linked to this article.

## Supplementary information

Supplementary Information Supplementary Figs. 1–18, Tables 1–11 and Text.

Reporting Summary

Peer Review File

## Source data

Source Data Figs. 2–5 Source data for Figs. 2–5.

## Data Availability

All the data to reproduce the results in this study are available via GitHub at https://github.com/schwallergroup/gollum. Source data are provided with this paper.

## Code availability

All the code to reproduce the results in this study is available via GitHub at https://github.com/schwallergroup/gollum and via Zenodo at https://doi.org/10.5281/zenodo.20625386 (ref. ^69^).
