# Supplementary material for: Large language models as uncertainty-calibrated optimizers for experimental discovery
Source: Nat Mach Intell. 2026 Aug 28;8(9):1466–77. doi: 10.1038/s42256-026-01283-z (PMC13585549; doi:10.1038/s42256-026-01283-z)
Supplement: Supplementary file 1 [file 42256_2026_1283_MOESM1_ESM.pdf]

nature machine intelligence

Supplementary information https://doi.org/10.1038/s42256-026-01283-z

Large language models as uncertainty-calibrated optimizers for experimental discovery

In the format provided by the authors and unedited

Contents

S1 Related work 2

S2 Technical background 4
- S2.1 Bayesian optimization . . . 4
- S2.2 Sequential decision process . . . 4
- S2.3 Gaussian processes and marginal likelihood optimization . . . 5
- S2.4 Deep kernel Gaussian processes . . . 5
- S2.5 Large language models . . . 6
- S2.6 Parameter-efficient LLM finetuning . . . 6
- S2.7 Pseudocodes . . . 7

S3 LLM and domain-specific representations 7
- S3.1 Molecular representations . . . 7
- S3.2 Reaction representations . . . 7
- S3.3 LLM representations . . . 8
  - S3.3.1 **Template construction** . . . 8
  - S3.3.2 Encoder-based models . . . 9
  - S3.3.3 Encoder-decoder models . . . 9
  - S3.3.4 Decoder-only models . . . 10
- S3.4 Representations overview . . . 10

S4 Benchmarking datasets 11
- S4.1 Synthetic organic chemistry . . . 11
- S4.2 Molecular optimization . . . 13
- S4.3 Material science and catalysis . . . 14
- S4.4 Analytic and process chemistry . . . 15

S5 Extended results 16
- S5.1 Tokenization per LLM type . . . 16
- S5.2 Which LLM layers carry the most information? . . . 16
- S5.3 Aggregated performance per optimization paradigm . . . 18
- S5.4 LLM pretraining and input format influence . . . 23
- S5.5 Chemical optimization as chocolate making . . . 23
- S5.6 Structure vs fit and BO performance . . . 24
- S5.7 Predictive accuracy and uncertainty calibration . . . 27
- S5.8 Which GOLLuM variant to use? . . . 28
- S5.9 BO results per representation type . . . 29

S6 Reproducibility 32
- S6.1 BO Initialization . . . 32
- S6.2 BO setup . . . 32
- S6.3 Implementation details . . . 32

S7 Supporting tables 34

S1 Related work

Adapting LLMs to specialized tasks through finetuning typically optimizes for predictive accuracy [1, 2]. Such domain adaptation neglects the dimension of epistemic uncertainty to indicate when model outputs should not be trusted. In this context, the challenge of extracting reliable uncertainties from LLMs for efficient BO has introduced diverse approaches that fall into four categories.

(1) Prompt-based methods like BOLIFT [3], estimate uncertainty by aggregating multiple LLM responses, while LLAMBO, LLM-GO and Sequential LLM-HPO [4–6] query LLMs as optimizers. (2) Embedding-based methods, such as BoChemian [7], model GPs on static LLM representations. (3) Surrogate conversion models either predict uncertainty through pretrained in-context regressor [4] or transform LLMs into Bayesian neural networks [8] through PEFT and Laplace approximation (LAPEFT [9] following Bayesian LoRA approach [10]). (4) Hybrid approaches like BOPRO [11] combine fixed LLM embeddings for GP-based acquisition optimization with prompt-conditioned LLM generation, using in-context examples to explore the solution space. BORA [12] uses a heuristic policy to regulate LLM's high-level reasoning and its suggestions for new regions of the search space to explore. While promising, these methods may face limitations from heavy prompt engineering to post-hoc uncertainty fixes. Our approach offers a solution by integrating uncertainty modeling *during* training within both the LLM and the surrogate model (GP) through LLM-based deep kernel strategy.

Previous works on integrating LLMs and GPs [3, 7, 8, 13] for chemical optimization primarily use LLMs as fixed feature extractors. Despite LLM's flexibility in converting diverse parameters into fixed-dimensional representations, these approaches essentially reduce their power to sophisticated encoding tools. The static embeddings struggle to match, let alone surpass, domain-specific features, creating an artificial barrier between LLM expressiveness and GP rigor.

The closest related approach to ours is the one by Kristiadi et al. [8]. It incorporates supervised LLM finetuning during optimization (for better prediction), followed by post-hoc Laplace approximation of learned weights (for uncertainty estimates). The sequential approach however, decouples uncertainty quantification from the learning process and optimizes for prediction accuracy (lower MSE loss) rather than optimization itself. Our method fundamentally differs by integrating LLMs directly into the GP framework as a deep kernel, making uncertainty quantification an integral part of the optimization objective through the GP marginal likelihood.

Existing deep kernel methods [14, 15] in chemistry constrain to using graph neural network kernels and domain specific representations. Advancing this concept, we demonstrate that LLM-based kernels provide richer embedding space and easier adaptation to various domains. In the latent space optimization, our approach relates to BO with variational autoencoders (VAEs). For example, [16] use VAE-BO for constrained optimization of molecules while [17] introduce explicit deep metric learning to structure the latent space. We elevate this approach by replacing pretrained VAEs with general purpose LLMs. Moreover, our joint optimization induces implicit metric learning, directly structuring the latent space without the need for explicit contrastive objectives.

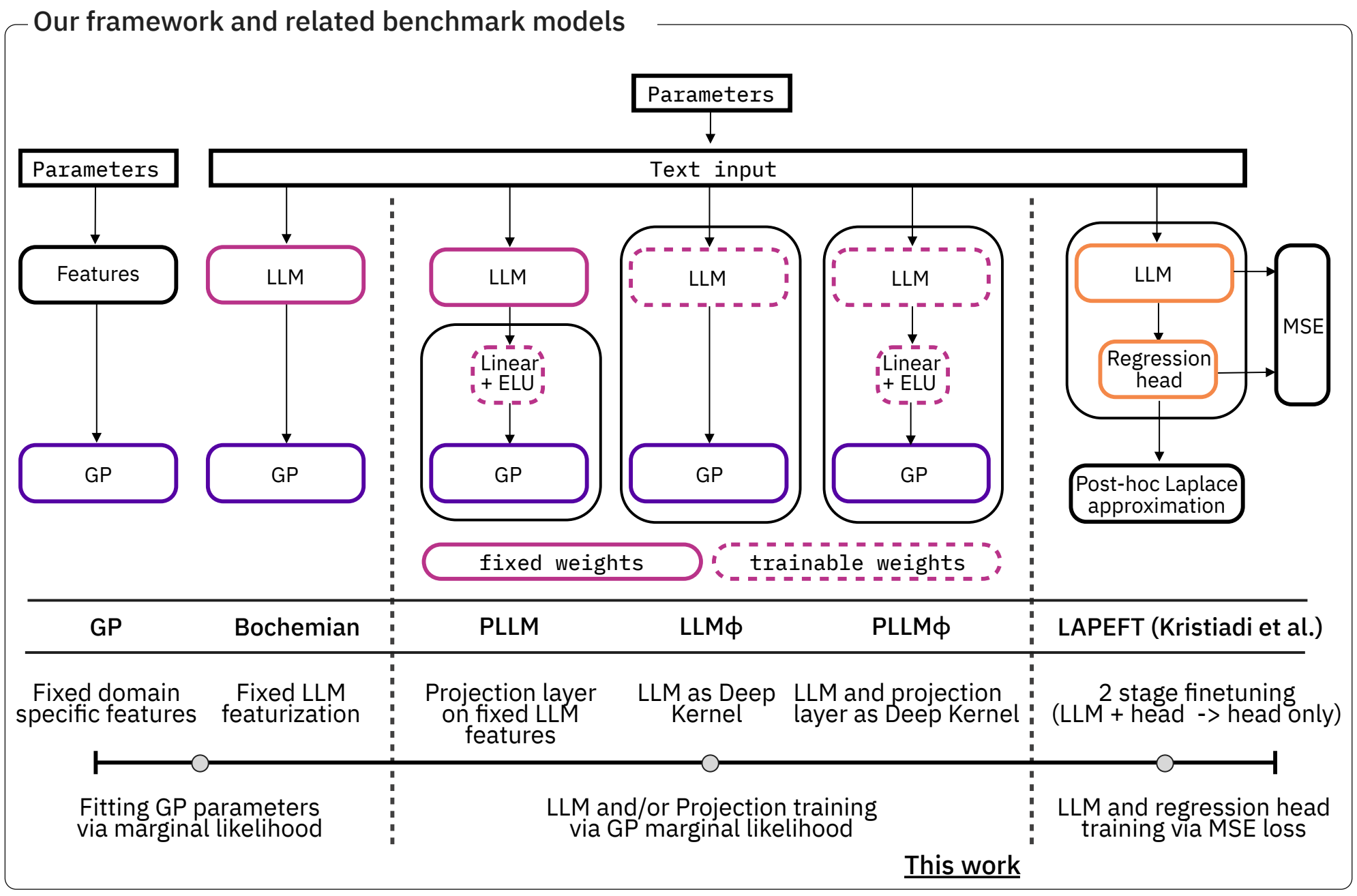

Fig. S1 Architectural comparison of our proposed framework and related benchmark models. The diagram illustrates the data and training flow for each approach. From left to right: A standard GP baseline using fixed domain-specific features. Bochemian [7], which uses a fixed LLM as a feature extractor. Our three proposed variants (PLLM, LLMϕ, and PLLMϕ), which are distinguished by their trainable components (a projection layer, the LLM itself, or both). Critically, our framework uses the GP's marginal likelihood to jointly train these components. This is contrasted with LAPEFT [8], which trains an LLM and regression head via an MSE loss and estimates uncertainty as a separate, post-hoc step.

Importantly, deep kernel learning in low-data regime has long been considered challenging due to issues like overfitting and training instability [18], with some approaches opting for coordinate ascent or bi-level optimization strategies to mitigate these issues [15, 19]. In contrast, we build a robust and stable training pipeline using standard back-propagation with separate learning rates for the LLM and GP components, avoiding the need for complex optimization schedules or custom regularization.

Operating in high-dimensional BO (HDBO) spaces (768 features with BERT-based models, 4096 with larger decoder types), our work demonstrates successful optimization that challenges conventional assumptions about HDBO limitations [20]. Through our empirical analysis, we identify key principles for effective HDBO: embeddings should structure following the GP's inductive bias. This organization of the embedding space plays a crucial role in overcoming dimensionality challenges that have traditionally constrained BO approaches. Recent work on HDBO has identified vanishing gradients in GP training and acquisition optimization as a key challenge, and proposed solutions based on informed lengthscale priors and local search heuristics [21]. Our approach complements this line of work by focusing on the structure of the representation space itself, showing that smoothness and generalization can emerge naturally when the

embedding is well-aligned with the GP kernel. Our observations contribute to emerging approaches in understanding and improving HDBO performance [21, 22].

Within chemistry, high-dimensional features are the default (one-hot encodings [23], fingerprints [24–28] quantum mechanical descriptors [29, 30]) but their selection remains challenging [13]. We overcome this problem through unified representation learning as an implicit objective of the optimization strategy itself. As a result, we achieve adaptive features for any optimization at hand, without requiring traditionally used expert-based descriptors.

S2 Technical background

S2.1 Bayesian optimization

Bayesian optimization (BO) is a suitable method for optimizing expensive-to-evaluate functions with unknown analytic form or gradients. Such problems are common in chemistry where experimental evaluations are often costly, time-consuming, and only available through real-world lab experiments. The primary objective of BO is to find:

$$\mathbf{x}^* = \arg\max_{\mathbf{x}\in\mathcal{X}} f(\mathbf{x}) \tag{1}$$

where $f : \mathcal{X} \to \mathbb{R}$ is the objective function over domain $\mathcal{X}$. In practical chemistry applications, we often work in a constrained domain $\mathcal{X}_{\text{pool}}$ with limited set of possible experimental conditions or molecular structures (e.g., feasible reaction conditions, available reagents or compound libraries). The optimization objective may involve maximizing reaction yield or selectivity, or minimizing properties such as reaction time, cost or toxicity.

S2.2 Sequential decision process

Bayesian optimization operates as a sequential decision-making process that balances exploration and exploitation. Key components include (1) a probabilistic surrogate model of the underlying objective function f and (2) an acquisition function α. The acquisition function guides the optimization process by proposing subsequent evaluation points. Common choices include expected improvement (EI [31]), probability of improvement (PI [32]), upper confidence bound (UCB [33], [34]), and Thompson sampling (TS [35]). Acquisition function selection over the points in the design space relies on the surrogate model and its predictive and uncertainty estimates. For example EI selects points that, in expectation, improve upon the current best observed value $f(\mathbf{x}_{\text{best}})$:

$$\alpha_{\text{EI}}(\mathbf{x}|\mathcal{D}_t) = \mathbb{E}_{p(f|\mathcal{D}_t)}[\max(f(\mathbf{x}) - f(\mathbf{x}_{\text{best}}), 0)] \tag{2}$$

In that sense, the choice of a surrogate model is critical to the success of BO. Gaussian Processes (GPs) are the most common choice due to their flexibility and ability to quantify uncertainty, making them particularly suitable for guiding the exploration of vast chemical spaces while operating in low-data regimes.

S2.3 Gaussian processes and marginal likelihood optimization

GPs provide a flexible non-parametric method for modeling unknown functions. A GP places a prior distribution

$$f(\mathbf{x}) \sim \mathcal{GP}(c, k(\mathbf{x}, \mathbf{x}')), \tag{3}$$

defined by a mean function c (typically 0 or constant) and a kernel function k encoding pairwise similarity between inputs and prior assumptions about function smoothness and variability. A common choice is the Matérn-5/2 kernel

$$k_{\text{Matérn-5/2}}(\mathbf{x}, \mathbf{x}') = \sigma^2 \left(1 + \frac{\sqrt{5}d}{\ell} + \frac{5d^2}{3\ell^2}\right) \exp\left(-\frac{\sqrt{5}d}{\ell}\right), \tag{4}$$

where $d = \|\mathbf{x} - \mathbf{x}'\|_2$, ℓ is the lengthscale, and σ^2 is the signal variance.

Given training data $\mathcal{D} = \{(\mathbf{x}_i, y_i)\}_{i=1}^n$, the GP posterior allows closed-form prediction of the function at new points, along with uncertainty estimates. Crucially, the GP hyperparameters $\theta = \{c, \ell, \sigma^2, \sigma_n^2\}$ are learned by maximizing the marginal likelihood of the data:

$$\mathcal{L}(\theta) = \log p(\mathbf{y}|\mathbf{X}, \theta) = -\frac{1}{2}(\mathbf{y}^\top \mathbf{K}_\theta^{-1} \mathbf{y} + \log|\mathbf{K}_\theta| + n \log 2\pi) \tag{5}$$

where $\mathbf{K}_\theta$ is the kernel matrix incorporating observation noise σ_n^2, evaluated on the training inputs using kernel parameters (ℓ, σ^2). If a constant mean function c is used, the targets $\mathbf{y}$ are centered as $\mathbf{y} - c\mathbf{1}$ during marginal likelihood computation. In the standard fixed-feature setting, input $\mathbf{x}$ is mapped to a feature vector via a static transformation (e.g., molecular fingerprints or frozen LLM embeddings), and the GP operates solely on these representations. The optimization updates θ, adapting the GP's inductive bias to the fixed feature space.

S2.4 Deep kernel Gaussian processes

Deep kernel Gaussian processes (DKGPs) introduce an additional parameter set ϕ to the optimization objective by integrating neural network-based feature transformations into the GP kernel. Formally, the kernel function becomes:

$$k_{\theta,\phi}(\mathbf{x}, \mathbf{x}') = k_\theta(g_\phi(\mathbf{x}), g_\phi(\mathbf{x}')),$$

where $g_\phi(\cdot)$ is a learned data representation parameterized by ϕ. This formulation enables the model to adapt the input space to the task at hand while preserving the uncertainty modeling properties of the GP.

The transformation g_ϕ can take the form of any neural architecture suitable for the data modality. The original paper applied the DKGP architecture on regression tasks with images using convolutional neural networks [36], while subsequent works have extended it to structured chemical domains using graph neural networks [14]. As detailed in the main section, we apply this framework to textual chemical representations by using large language models (LLMs) as the deep kernel feature extractor. This allows us to incorporate both pretrained domain knowledge and task-specific adaptation within the BO loop.

S2.5 Large language models

LLMs process textual inputs by converting them into dense vector representations through a sequence of tokenization, embedding and attention-based transformations. Tokenization involves the process of splitting the input text into subword units (tokens) using a model-specific vocabulary (e.g., SentencePiece [37], Byte-Pair Encoding [38]). The tokens are mapped to continuous vectors via learned embedding layers and passed through multiple self-attention layers that capture contextual relationships between tokens.

LLMs can follow different architectural designs: encoder-only (e.g., BERT [39]), decoder-only (e.g., Qwen [40]), and encoder-decoder (e.g., T5 [41]). Encoder-based models process the full input bidirectionally and are suited for classification and regression. Decoder-only models generate text autoregressively with causal masking. Encoder-decoder models combine both components and are often used for sequence-to-sequence tasks. The architecture choices impact the structure and pooling strategies used to extract unified representations from the variable-length token sequences.

Pooling refers to the process of aggregating a sequence of token-level representations produced by a language model into a single fixed-dimensional embedding. Encoder-based models often use the hidden state corresponding to the special [CLS] token or apply mean-pooling across token embeddings. Decoder-only models typically use the final hidden state of the last non-padding token. For encoder-decoder models, pooling is applied over the encoder-side hidden states.

S2.6 Parameter-efficient LLM finetuning

Although pretrained LLM embeddings encode rich semantic information, they are not tailored to specific downstream tasks. In that sense, adapting LLMs through finetuning allows for better task-specific capabilities. However, updating LLM weights can be computationally prohibitive due to their large size (often billions of parameters in modern LLMs). Parameter-efficient finetuning (PEFT), however, provides a recipe for LLM task alignment by adapting a smaller subset of parameters while leaving the majority of the model unchanged.

One such approach is Low-Rank Adaptation (LoRA) [42], which injects trainable low-rank matrices into existing weight layers. Instead of updating a weight matrix $W \in \mathbb{R}^{d \times k}$, LoRA learns a low-rank update of the form:

$$\Delta W = AB, \quad \text{where } A \in \mathbb{R}^{d \times r}, \quad B \in \mathbb{R}^{r \times k}, \quad r \ll \min(d, k)$$

The adapted weight becomes $W' = W + \Delta W$, allowing task-specific learning with a parameter count that scales with r, the rank of the decomposition. This method allows efficient finetuning and mitigates the risk of catastrophic forgetting by preserving the pretrained weights.

Algorithm 1 Bayesian Optimization

Require: Initial dataset $\mathcal{D}_0 = \{(\mathbf{x}_i, y_i)\}_{i=1}^{n_0}$, candidate pool $\mathcal{X}_{\text{pool}}$, budget T, objective function f

Initialize surrogate model (e.g., GP) using $\mathcal{D}_0$
for $t = 1$ to T **do**
    Fit surrogate model to current data $\mathcal{D}_{t-1}$
    for all $\mathbf{x} \in \mathcal{X}_{\text{pool}}$ **do**
        Compute acquisition value $\alpha(\mathbf{x} \mid \mathcal{D}_{t-1})$
    end for
    Select next point: $\mathbf{x}_t = \arg\max_{\mathbf{x} \in \mathcal{X}_{\text{pool}}} \alpha(\mathbf{x} \mid \mathcal{D}_{t-1})$
    Evaluate objective function: $y_t = f(\mathbf{x}_t)$
    Update dataset: $\mathcal{D}_t = \mathcal{D}_{t-1} \cup \{(\mathbf{x}_t, y_t)\}$
    Remove $\mathbf{x}_t$ from $\mathcal{X}_{\text{pool}}$
end for
return Best observed point: $\mathbf{x}^* = \arg\max_{(\mathbf{x},y) \in \mathcal{D}_T} y$

S2.7 Pseudocodes

S3 LLM and domain-specific representations

S3.1 Molecular representations

Molecular representations have been extensively studied in chemistry, leading to (1) fingerprints [24, 26, 43], (2) Simplified Molecular Input Line Entry System (SMILES) strings [44, 45], (3) molecular graph-based features [46] or (4) more physics-informed descriptors derived from electronic structure calculations [47]. Each of these representations encodes different aspects of molecular structure and properties making their utility task-dependent.

For example, molecular fingerprints can be effective for tasks involving structural similarity or substructure-driven properties, while quantum-derived features may be better suited for tasks involving electronic properties. For applications in BO, these representations typically require specialized kernel functions to capture relevant similarity [48].

To compare domain-specific representation to general LLM-based ones, we set molecular fingerprints as input to a GP in all molecular property optimization benchmarks.

S3.2 Reaction representations

Chemical reactions, on the other hand, attach an additional layer of complexity beyond molecular representation including reaction conditions and procedural descriptions. In that sense, they present a unique challenge for machine learning due to an increased complexity and heterogeneous nature. Reaction conditions typically comprise multiple parameter types: numerical values (temperature, concentration, time), categorical variables (catalyst type, solvent choice), and detailed procedural descriptions, making

Algorithm 2 Bayesian Optimization with LLM-based Deep Kernel GP

Require: Initial dataset $\mathcal{D}_0 = \{(\mathbf{x}_i, y_i)\}_{i=1}^{n_0}$, candidate pool $\mathcal{X}_{\text{pool}}$, budget T

for $t = 1$ to T **do**
  Initialize parameters ϕ (LLM) and θ (GP)
  Train LLM-GP model:
  repeat
    Compute embeddings: $\mathbf{z}_i = g_\phi(\mathbf{x}_i)$ for all $(\mathbf{x}_i, y_i) \in \mathcal{D}_{t-1}$
    Evaluate GP marginal log-likelihood $\log p(\mathbf{y} \mid \mathbf{z}, \theta)$
    Update ϕ, θ
  until convergence
  Compute acquisition on candidate pool:
  for all $\mathbf{x}_j \in \mathcal{X}_{\text{pool}}$ **do**
    $\mathbf{z}_j = g_\phi(\mathbf{x}_j)$
    Compute $\alpha(\mathbf{z}_j; \theta)$
  end for
  Select next input: $\mathbf{x}_t = \arg\max_{\mathbf{x}_j} \alpha(\mathbf{z}_j)$
  Observe outcome: $y_t = f(\mathbf{x}_t)$
  Update dataset: $\mathcal{D}_t = \mathcal{D}_{t-1} \cup \{(\mathbf{x}_t, y_t)\}$
  Remove $\mathbf{x}_t$ from $\mathcal{X}_{\text{pool}}$
end for
return Best input: $\arg\max_{(\mathbf{x},y)\in\mathcal{D}_T} y$

their featurization challenging. Reaction representations used in ML range from simple one-hot encodings [23] to more elaborate reaction fingerprints [24–27], quantum mechanical descriptors [29, 30] and learned representations [28].

We make extensive use of Differential Reaction Fingerprints (DRFPs) [27], previously shown to achieve state-of-the-art results on reaction optimization tasks compared to a variety of molecular and reaction descriptors [13]. Generated by first computing circular fingerprints for each reactant and product and then taking their symmetric difference, DRFPs highlight the structural changes during the reaction while remaining computationally cheap. We input DRFPs to GP in reaction optimization tasks with available reaction SMILES. For other chemical optimization tasks we generate features through one-hot encoding of categorical variables, concatenated with numerical parameter values.

S3.3 LLM representations

S3.3.1 Template construction

We define each task t through a standardized template: $t = \text{template}(\{\text{parameters}, \text{values}\})$ where the template converts various parameter types into a structured text format:

```
The reaction was prepared with:
temperature: {numerical_value}°C
solvent: {solvent_smile}
```

`ligand: {ligand_smile}`

S3.3.2 Encoder-based models

Encoder-based

Encoder-based language models, originally developed for natural language understanding tasks, have long been used to generate fixed-dimensional vector representations of text. These models, typically based on the transformer encoder architecture, process input sequences bidirectionally. In that sense, they have been widely adopted in various downstream tasks such as classification, clustering, and semantic similarity.

With the recent evolution toward larger-scale pretraining, encoder-only models have also followed the trajectory of large language models, yielding high-capacity embedding models suitable for diverse domains beyond natural language. These modern embedding models are trained on massive corpora with contrastive or retrieval-oriented objectives, making them particularly effective for extracting general-purpose sentence and document embeddings.

In this work, we evaluate three encoder-based embedding models on tasks of representing chemical procedures and reaction descriptions for BO.

ModernBERT [49]: A compact and efficient embedding model trained with a retrieval objective, designed for high-speed and high-quality sentence representations.

UAE-Large [50]: The Universal Alignment Embedding model, trained for multilingual and multimodal generalization with a strong emphasis on alignment across domains.

MXBAI-Embed [51]: A large-scale embedding model from Mixedbread-AI, trained to preserve semantic similarity across a broad range of tasks, including code, math, and text.

S3.3.3 Encoder-decoder models

Encoder-decoder architectures, such as the T5 family [41], consist of two transformer modules: an encoder that processes the input sequence and a decoder that generates output sequences, typically in an autoregressive fashion. For embedding tasks, representations are typically extracted from the encoder side, which embeds the input text into a fixed-length latent representation. Compared to encoder-only models, encoder-decoder architectures are often pretrained with sequence-to-sequence objectives such as masked span prediction or denoising, making them well-suited for tasks involving paraphrasing, summarization, or input–output alignment.

We evaluate the encoder outputs from three encoder-decoder models:

T5 (base variant) [41]: A widely-used general-purpose model pretrained on a multi-task mixture of unsupervised and supervised NLP tasks. We use the encoder outputs as text embeddings.

T5Chem [52] : A domain-adapted variant of T5, finetuned on chemical tasks using the GT4SD framework. It is trained on a multitask mixture involving molecular property prediction, retrosynthesis, and chemical text modeling, making it more specialized for chemistry-related input sequences.

Instructor [53]: An instruction-tuned encoder-decoder model trained on natural language–task pairs. It learns to produce embeddings guided by a task description (e.g.,

"Represent the reaction for similarity search"), making it suitable for alignment-sensitive downstream applications.

S3.3.4 Decoder-only models

Decoder-only architectures, exemplified by models in the GPT [54] family, generate outputs autoregressively by predicting each token conditioned on all previous ones. While traditionally used for generation tasks, these models can also produce dense representations of input text by extracting hidden states from specific tokens (e.g., the final token or special marker tokens). Decoder-only models are typically pretrained with causal language modeling objectives and operate unidirectionally, which distinguishes their contextual encoding behavior from encoder-based models.

In this work, we evaluate several decoder-style models for embedding chemical procedures and reaction descriptions:

OpenAI embeddings [55] A widely-used commercial API that provides text embeddings via proprietary transformer models. While the architectural details are not public, we assign them to decoder-style GPT family.

Qwen2-7B-Instruct [40] A large-scale instruction-tuned language model from Alibaba, based on a decoder-only architecture. We use this model in embedding mode by extracting the hidden state of the last non-padding token.

GTE-Qwen2-7B-Instruct [56] A retrieval-optimized variant of Qwen2, finetuned to produce sentence-level embeddings with improved performance on similarity and ranking tasks.

LLaMA 3–8B [57] Meta's open LLaMA 3 model in its original instruction-tuned form, without additional adaptation for embeddings.

LLM2Vec models [58] We also evaluate decoder-only LLMs adapted for embedding tasks using the LLM2Vec framework [58]. These models, such as LLM2Vec–Meta-Llama-3 and LLM2Vec–Mistral-7B, are trained with masked next token prediction (MNTP) to enable bidirectional context modeling and use supervised mean pooling over selected internal layers. This adaptation allows decoder-only transformers to behave similarly to encoder models in embedding quality, while preserving their original architecture.

S3.4 Representations overview

We build LLM representations with models from HuggingFace (HF) selected through their base architecture and the results on MTEB in summarization task. In Table S1 we give an overview of all representations used in this paper alongside specifics on the dimensionality, pooling, architecture, pretraining, and connections to chemistry. We also include HF sources for LLM-based representations and links to chemistry-related molecular fingerprints and DRFP featurization methods.

Model	Arch.	Pretrain. obj.	Pool	Chem.	Dim.	Source
Molecular						
Fingerprints	/	/	/	Yes	2048	Morgan
Reaction						
DRFP	/	/	/	Yes	2048	DRFP
Encoder						
ModernBERT	Encoder	Retr. contrastive	CLS	No	768	HF
MXBAI-Embed	Encoder	General-purpose	CLS	No	1024	HF
UAE-Large	Encoder	Align./Multimod.	CLS	No	1024	HF
Enc-Dec						
T5-Base	Enc-Dec	Mask span pred.	Mean	No	768	HF
T5Chem	Enc-Dec	Chem multitask	Mean	Yes	768	HF
Instructor	Enc-Dec	Instr. align.	WeighPart.† Mean		768	HF
Decoder						
OpenAI Embedding	Decoder*	Proprietary	Unk.	Unk.	3072	OpenAI
Qwen2-7B-Instruct	Decoder	Instr. tuning	Last	No	3584	HF
GTE-Qwen2	Decoder	Contrastive retr.	Last	No	3584	HF
LLM2Vec–LLaMA3	Decoder	Supervised pool.	Last	No	4096	HF
LLM2Vec–Mistral	Decoder	Supervised pool.	Last	No	4096	HF
LLaMA 3–8B	Decoder	Instr. tuning	Last	No	4096	HF

Table S1 Overview of data representations used in our experiments, including architecture, pretraining objective, pooling strategy, chemistry adaptation, and embedding dimensionality and direct link to the source.

S4 Benchmarking datasets

S4.1 Synthetic organic chemistry

Buchwald-Hartwig reactions

We performed all initial investigations on a set of Buchwald-Hartwig (BH) reactions with the task of optimizing yield (0-100%). This dataset consists of 3955 reactions spanning across five distinct products (BH1-BH5). The data originates from a high-throughput experimentation (HTE) study published by Ahneman et al. [29]. For each product, reactions were evaluated based on their percentage yield as determined by HPLC analysis. The design space is combinatorial across 15 reactants (aryl halides), 22 additives, 4 ligands, and 3 bases in DMSO solvent.

In all initial BO experiments (unless explicitly stated as in T5Chem-SMILES), we represented reactions through procedural text template describing the reaction conditions in natural language. Moving forward to benchmarking against other models, we featurized reactions based on reaction SMILES, to ensure fair comparison to models that report best performance when using this representation (LAPEFT). Moreover,

[1]* While OpenAI Embedding model architecture is not publicly disclosed, we assign a decoder-style structure based on the GPT family.
[2]† Partially chemistry-aligned through prompting instructions like "Represent the chemical reaction."

Table S2 Benchmark overview. The 23 optimization tasks grouped by chemistry domain, with dataset size (n) and optimization objective.

Domain	Benchmark	n	Objective
Organic chemistry	Buchwald–Hartwig (BH1–BH5) [29]	≈791	maximize yield (per aryl-halide product; BH1–BH5)
	Additive screens (Additives1–4) [59]	720	maximize product response (Ni-catalysed)
	Suzuki–Miyaura [60]	5760	maximize cross-coupling yield
	Pd-sulfonamide coupling [61]	585	maximize AP yield
	Reductive amination [62]	768	maximize conversion
Analytic & process chemistry	Catechol rearrangement [63]	1227	maximize scalarized yield/selectivity
	HPLC [64]	1386	maximize chromatographic peak response
	Fullerenes (C_{60} adducts) [65]	246	maximize product mole fraction
Materials & catalysis	C2 yield [3]	1180	maximize C2 yield (methane oxidative coupling)
	OER [64]	2121	minimize catalyst overpotential
	Vapdiff [64]	918	maximize crystal score
Molecular property optimization	Redox-mer [66]	1407	minimize redox potential
	Solvation [66]	1407	minimize solvation energy
	Kinase [67]	10449	minimize docking score (kinase inhibitors)
	Photoswitch [68]	392	maximize π–π^* transition wavelength
	PCE [69]	10000	maximize power conversion efficiency

this dual representation allowed us to evaluate and establish robustness to the impact of different input formats on model performance.

Additive screening

This dataset originates from a study on organic additives' influence on the reactivity of complex Ni-catalysed reactions in a high-throughput experimentation (HTE) setup [59]. It covers a wide range of screened additives (720) across four different reactions (Additives 1-4) and measures their effect on UV210 product area absorption. The challenge with traditional featurization methods in this dataset lies in the sole variability of the additive in the design space, while the other reaction parameters remain fixed. In such a setup, traditional one-hot encoding techniques would yield results similar to random search. Previous approaches report success with DRFP representation, comparing its performance to a set of molecular descriptors [13]. Compared to this method, we achieve better results while representing the reaction SMILES through LLM embeddings and optimizing with our GP-guided finetuning approach.

Suzuki-Miyaura reactions

Suzuki-Miyaura dataset is a reaction optimization benchmark that includes high-throughput evaluated Suzuki-Miyaura cross-coupling reactions, originally introduced by Perera et al. [60]. The dataset was generated using an automated nanomole-scale synthesis platform designed to explore large combinatorial reaction spaces efficiently. It contains 5760 reactions across varied combinations of 7 unique electrophile, 4 nucleophile, 11 ligands (plus one blank), 7 bases (plus one blank), 4 solvents and Pd(OAc)2 as precatalyst, while the optimization objective is maximizing yield.

Reductive amination reactions

Reductive amination dataset [62] is a reaction optimization benchmark introduced as part of a nanoscale ultrahigh-throughput experimentation (ultraHTE) study on reaction miniaturization. The benchmark evaluates the reductive amination diversification of staurosporine across a full-factorial screen of 768 experiments, obtained by combining 16 carbonyl building blocks (8 aldehydes and 8 ketones) with 48 reaction conditions. The reaction conditions vary (i) acetic acid loading (1, 3, or 5 equiv.), (ii) titanium isopropoxide (TTIP, $Ti(O\mathit{i}Pr)_4$) loading (1, 2, 3, or 5 equiv.), (iii) solvent (DMA or NMP), and (iv) reaction concentration (50 or 100 mM). The optimization objective is to maximize product formation, reported as conversion (%) for each substrate–condition pair.

Sulfonamide coupling reactions

The Pd-catalysed sulfonamide coupling dataset [61] is a reaction optimization benchmark derived from a prospective high-throughput experimentation campaign. The authors define a full factorial reaction condition space comprising 82 ligands, 11 solvents, 8 bases, and 3 palladium precursors (21,648 total combinations), targeting *multi-objective* optimization of area-percent (AP) yield and AP selectivity. From this design space, an experimentally measured dataset of 585 reaction conditions was collected using HTE and released as a virtual benchmark for comparing optimization algorithms. In our work, we use this experimentally evaluated subset for Bayesian optimization over the discrete condition variables, with objectives given by AP yield.

S4.2 Molecular optimization

We selected the benchmark datasets from Kristiadi et al. [8] to both test our model on molecular property optimization and compare directly to their approach (LAPEFT). Moreover, we use molecular SMILES as the textual representation of the data following their best practice and offering a fair comparison between the methods. The five datasets we present in this work span diverse scientific applications and optimization objectives:

- Redox (1,407 samples): minimize redox potential for flow battery materials [66],
- Solvation (1,407): minimize solvation energy [66],
- Kinase (10,449): minimize docking score in kinase inhibitors [67],
- Photoswitch (392): maximize the $\pi - \pi^*$ transition wavelength* in organic photoswitches [68],
- PCE (10,000): maximize power conversion efficiency of photovoltaic materials [69].

All objectives are continuous and we use molecular fingerprints as a chemistry-related featurization for the baseline GP comparison.

S4.3 Material science and catalysis

To move beyond reaction and molecular optimization we include two datasets from `Olympus` [64], involving catalysis and crystallization, together with C2 yield optimization from [3], all of which involve continuous or mixed-variable optimization objectives. These datasets are commonly used in autonomous discovery and closed-loop optimization studies.

Catalyst optimization – C2 yield

We evaluate our method on optimizing methane oxidative coupling (OCM) using a subset of 1180 reactions from [3]. Each reaction involves synthesizing a supported catalyst (e.g., $Mn{-}Na_2WO_{4/}BN$) by impregnating a solid support (typically BN) with a solution of up to three metal precursors in defined molar ratios. Additional parameters include reaction temperatures (typically ≈900 °C), with controlled gas flows (CH_4, O_2, Ar) and contact times. The objective is to maximize C2 yield, a measure of desirable product formation. The original study already provides a textual template of reactions in this dataset, which we used for testing our method on diverse input formatting. For the standard GP baseline we featurize the data by concatenating numerical parameters and one-hot encoded categorical values.

OER (Oxygen evolution reaction catalysts).

This dataset comprises 2,121 samples describing compositions of high-throughput screened catalysts for the oxygen evolution reaction. Each data point represents a combination of elemental loadings (Ni, Fe, Co, Mn, Ce, La) constrained to sum to 1. The optimization goal is to minimize the overpotential, a key descriptor of catalytic efficiency.

- **Target:** Overpotential (continuous)
- **Features:** 6 discrete fractional loadings

Vapdiff crystallization (crystal score).

This dataset reports the outcomes of vapor diffusion crystallization experiments across 918 combinations of organic, solvent, and inorganic conditions. The target is an *ordinal* score representing crystallization quality, with categorical and continuous inputs describing the experiment setup.

- **Target:** Crystal score (ordinal)
- **Features:** 10 variables (categorical, continuous, discrete)

Similarly to C2 yield optimization, we featurize the categorical variables through one-hot encoding and concatenate these vectors to the remaining numerical parameters for the input to the standard GP baseline.

S4.4 Analytic and process chemistry

HPLC (High-Performance Liquid Chromatography).

This dataset includes 1,386 data points measuring peak response from an automated HPLC system as a function of six continuous process parameters such as flow rate, sample volume, and wait time. The objective is to maximize the peak signal (measured by photo degradation response).

- **Target:** Photo degradation (continuous)
- **Features:** 6 continuous parameters

Catechol rearrangement reactions

The Catechol Benchmark dataset [63] is an analytic and process chemistry reaction optimization benchmark collected in a transient continuous-flow platform with online quantification of reaction outcomes. It reports 1227 experimentally measured process conditions for an allyl-substituted catechol rearrangement, where the controllable inputs are (i) a pair of solvents and their mixture ratio (parameterized by the fraction of solvent B, b), (ii) reactor temperature T, and (iii) residence time τ. The outputs are the measured yields of the starting material (SM) and two observed products (P_2 and P_3).

For Bayesian optimization, we follow the scalarized objective defined in the original study, which rewards total product yield and selectivity toward Product 2 while penalizing harsher operating conditions:

$$f(S_A, S_B, b, \tau, T) = \lambda_1(P_2 + P_3) + \lambda_2 \frac{P_2}{P_2 + P_3} - \lambda_3 \frac{T - 175}{50} - \lambda_4 \tau,$$

with weights $\lambda_1 = 5$, $\lambda_2 = 1$, $\lambda_3 = 3$, and $\lambda_4 = \frac{1}{20}$, and we maximize f over the experimentally observed design space.

Buckminsterfullerene adducts (Fullerenes)

The Fullerenes dataset ("Buckminsterfullerene adducts") is a process-chemistry benchmark distributed through the `Olympus` framework, originally reported by Walker et al. [65]. It captures the production of *o*-xylenyl adducts of C_{60} in a flow reactor, where three continuous process variables are tuned to maximize the mole fraction of the desired product: reaction temperature (100–150 °C), reaction time (3–31 min), and the relative concentration (ratio) of sultine to C_{60} (1.5–6.0). The benchmark contains 246 experimentally measured samples on a three-factor fully factorial grid with six levels per factor and is commonly used as a low-dimensional, noisy optimization surface for benchmarking Bayesian optimization and experiment-planning methods [64].

S5 Extended results

S5.1 Tokenization per LLM type

We investigate the impact of different pooling strategies on the quality of LLM embeddings for Bayesian optimization. Since LLMs produce variable-length token sequences, pooling plays a critical role in converting these sequences into fixed-size representations used by the GP surrogate. Our ablation reveals a clear interaction between model architecture and pooling choice. For encoder-only models, CLS token pooling outperforms alternatives – last token pooling dilutes the informative signal captured in the CLS token – specifically trained to represent global context. In contrast, decoder-only models tend to collapse all inputs to similar representations when the starting token is pooled, leading to duplicates and unsuccessful optimization. Here, last-token pooling aligns with the autoregressive structure and yields substantially better results. For encoder-decoder models, we observe lower differences in performance across pooling strategies, though mean pooling shows a slight edge in consistency.

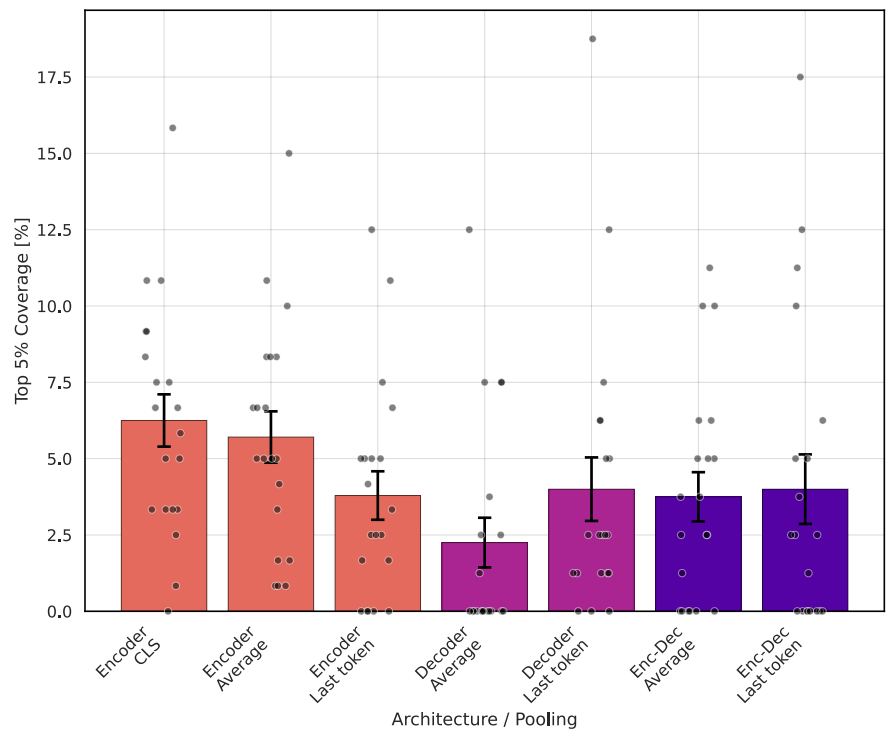

Fig. S2 Tokenization pool strategy per LLM type. We compare the pooling strategies across LLM types. Encoder-based models benefit from utilizing the CLS token, unlike decoder architectures where this token collapses all inputs to duplicated representation (hence not appearing in the results). For decoder-based architectures, last token pooling improves results over token averaging. For encoder-decoder models, the difference between average and last token pool is less pronounced, however with a lower variability for mean pooling. Bar heights show the mean Top-5% coverage with error bars giving the standard error of the mean (s.e.m.); overlaid dots are the individual seed runs. Results are on the BH1 reaction with 25 BO iterations, repeated over 10 seeds ($n = 10$).

Based on these findings, we adopt CLS pooling for encoder models, last-token pooling for decoder models, and mean pooling for encoder-decoder models throughout the main experiments. Figure S2 summarizes the performance differences across model types and pooling methods. The pooling choices ensure meaningful input representations across LLM types, and deviations from optimal setup can lead to noticeable performance drops within the fixed-feature setting (e.g., up to 40% in top-5 discovery rate between CLS and last-token pool for encoder models).

S5.2 Which LLM layers carry the most information?

We further examine which subset of LLM layers is most effective to target during PEFT. Since full model finetuning within the GP kernel is infeasible for large LLMs, we use LoRA to adapt only a fraction of the model weights. The choice of which layers to modify has a strong effect on optimization performance. Inspired by the intuition that higher (deeper) transformer layers encode more task-relevant semantics, we evaluate

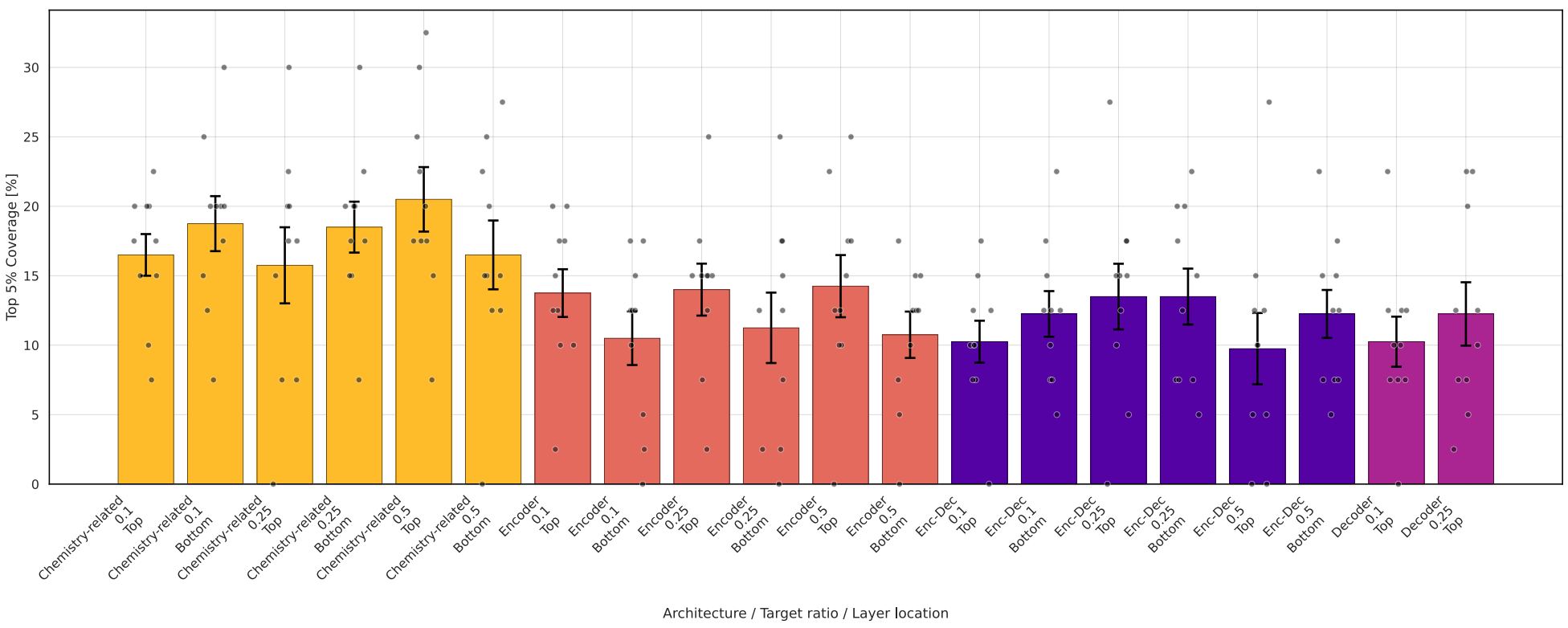

Fig. S3 Performance comparison of PEFT strategies across LLM architectures. We vary the proportion (10%, 25%, 50%) and location (top vs. bottom) of targeted linear layers using LoRA. Results show that top-layer finetuning consistently outperforms bottom-layer updates for encoder-only and decoder-only∗ models. For encoder-decoder models, performance is more consistent across layer locations. Bar heights show the mean Top-5% coverage, error bars the standard error of the mean (s.e.m.), and overlaid dots the individual runs contributing to each bar. Based on these findings, we fix the default to targeting the top 25% of linear layers.

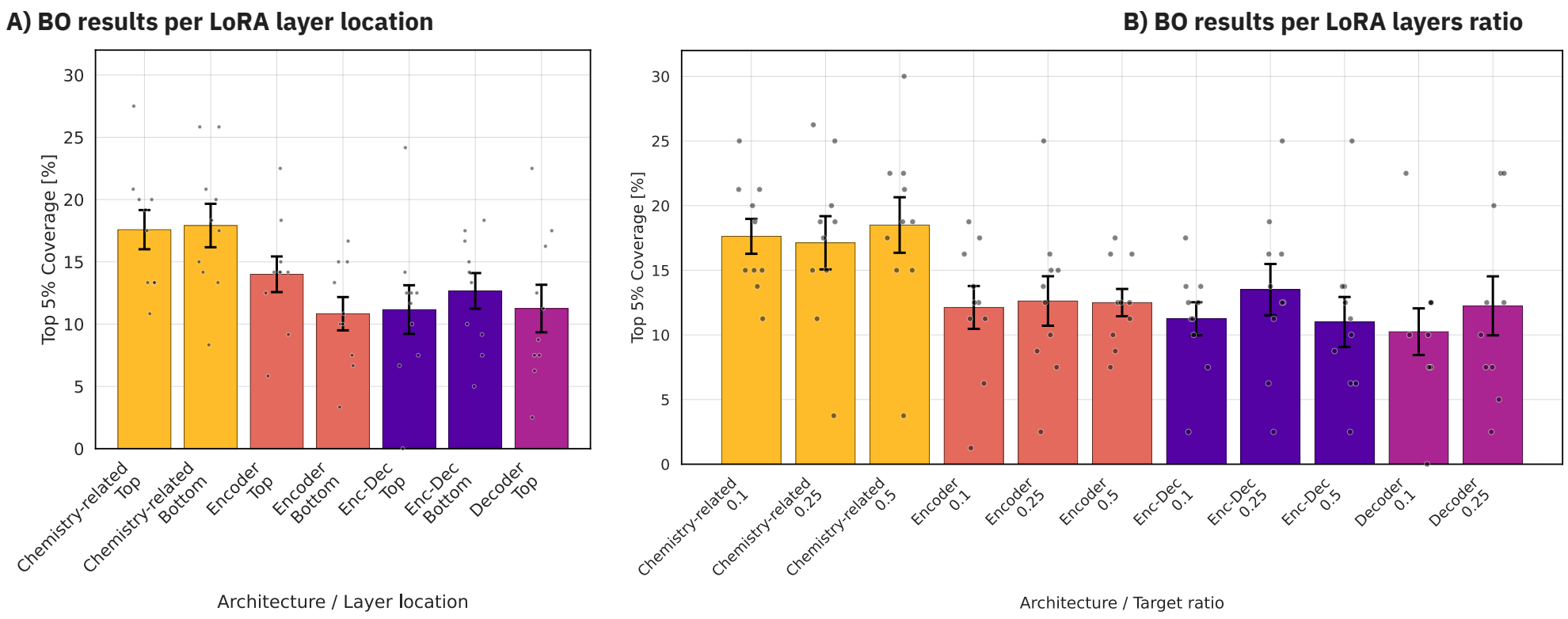

Fig. S4 Breakdown of results from Figure S3, highlighting the effect of LoRA layer location (top vs. bottom) and proportion (10%, 25%, 50%) on BO performance. Targeting bottom layers in decoder-only models (e.g., Qwen2-7B) resulted in numerical instabilities during optimization while higher proportion (50%) resulted in out-of-memory issues.

LoRA targeting strategies by varying both the location (top vs. bottom layers) and proportion (10%, 25%, 50%) of modified linear layers.

Figures S3 and S4 show the results across four (we now separate the encoder-decoder models into chemistry-related–T5Chem and base T5) LLM architectures. For encoder-only, targeting the top layers consistently outperforms bottom-layer finetuning. This aligns with the well-established view that deeper layers in such models encode more abstract and domain-specific representations. Importantly, we omit bottom-layer results for decoder-only models (e.g., Qwen2-7B) due to instability and numerical issues encountered during training, likely caused by incompatible LoRA insertions in

early layers, and only show results of the different ratio of LoRA adapted top layers (10% and 25%).

Interestingly, encoder-decoder models (both T5 and chemistry-specialized T5Chem) show competitive performance even when targeting bottom layers, though the top 25% for T5 still yields slightly better or equally stable results. This suggests that meaningful information may be distributed across layers in encoder-decoder setups, potentially due to the dual role of encoding and decoding steps. For T5Chem, targeting the bottom 50% of linear layers with LoRA yield the best results in the ablation (Figure S3). This observation could potentially justify targeting all layers with LoRA in chemistry-related architectures which could contribute to even better results. Nevertheless, to ensure a consistent and generalizable comparison across LLM sizes and types, we adopt a default strategy of targeting the top 25% of linear layers for all models. This decision achieves a balance between performance and computational efficiency, while also avoiding the overhead of architecture-specific tuning.

S5.3 Aggregated performance per optimization paradigm

To better understand when GOLLuM provides the strongest gains, we aggregate the Top-5% coverage metric across *optimization domains*, grouping benchmarks by the type of optimization landscape induced by their input representation: mixed-variables (categorical/continuous mixtures), numerical (continuous/discrete), and fingerprints (molecular or reaction fingerprint descriptors).

Figure S5 summarizes the aggregated Top-5% coverage. Across mixed-variable and numerical domains, GOLLuM variants consistently achieve higher coverage than GP-only and alternative LLM baselines, indicating that coupling the probabilistic optimizer with learned LLM representations yields the clearest benefits when the input space is heterogeneous or continuous.

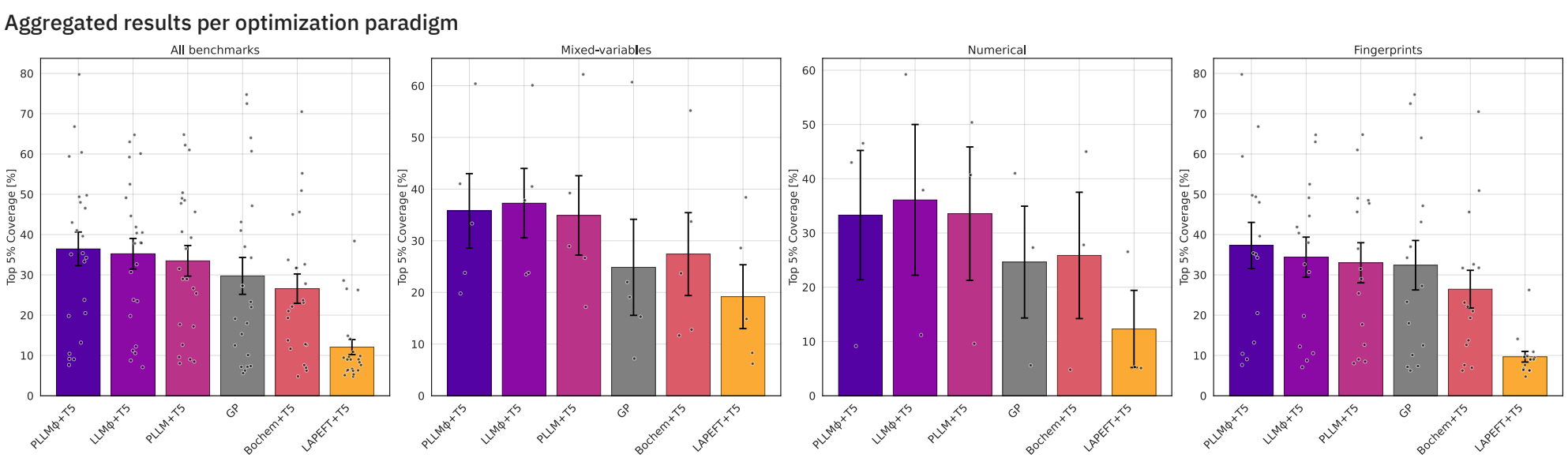

Fig. S5 Aggregated Top-5% coverage by optimization domain. Aggregation by optimization domain (All benchmarks, Mixed-variables, Numerical, Fingerprints). Bars report the mean Top-5% coverage (%) across benchmarks in each group, comparing GOLLuM variants (PLLM+T5 and PLLMϕ+T5, LLMϕ+T5) against GP, Bochemian+T5, and LAPEFT+T5. Overlaid dots are the per-benchmark mean coverages within each group and error bars the standard error of the mean (s.e.m.) across those benchmarks (n = 23, 5, 3, and 15 benchmarks for All benchmarks, Mixed-variables, Numerical, and Fingerprints, respectively). Overall, GOLLuM provides the clearest gains in mixed-variable and numerical domains.

[1]* Targeting bottom layers in decoder-only models (Qwen2-7B) led to numerical instabilities in optimization.

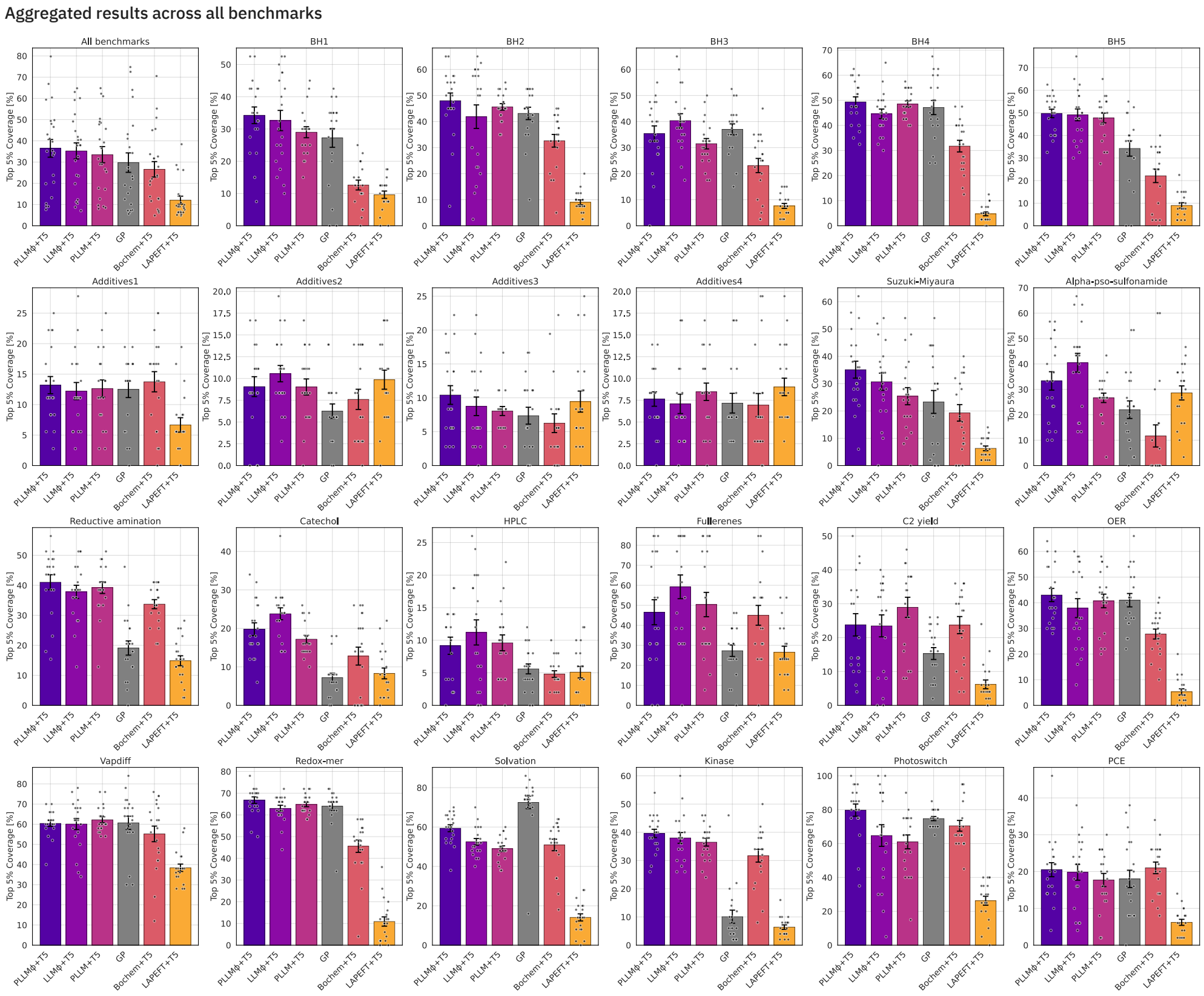

Fig. S6 Top-5% coverage per benchmark. Per-benchmark breakdown of Fig. S5, reporting Top-5% coverage (%, higher is better) for each benchmark individually across methods. Bar heights are means; in each per-benchmark panel the overlaid dots are the individual seed runs ($n = 20$) with error bars showing the s.e.m. over seeds, whereas in the aggregate "All benchmarks" panel the dots are per-benchmark means ($n = 23$) with s.e.m. across benchmarks. Benchmarks are ordered by optimization domain for readability; we provide the complete benchmark-to-domain mapping provided in Table S5.3.

Domain	Benchmarks
Organic chemistry	BH1–BH5; Additives1–Additives4; Suzuki-Miyaura; Pd-sulfonamide coupling; Reductive amination
Analytic & process chemistry	Catechol; HPLC; Fullerenes
Materials & catalysis	C2 yield; OER; Vapdiff
Molecules	Redox-mer, Solvation, Kinase, Photoswitch, PCE

Table S3 Benchmark-to-domain mapping used for aggregated analyses in the main text.

Paradigm	Benchmarks
Mixed-variables	Pd-sulfonamide coupling; Reductive amination; Catechol; C2 yield; Vapdiff
Numerical	HPLC; Fullerenes; OER
Fingerprints	BH1–BH5; Additives1–Additives4; Suzuki-Miyaura; Redox-mer, Solvation, Kinase, Photoswitch, PCE

Table S4 Benchmark-to-paradigm mapping used for aggregated analyses. We group tasks by their input representation: *Fingerprints* tasks have available molecular or reaction fingerprints (e.g., DRFP [27]), *Numerical* use continuous or discrete numerical parameters, and *Mixed-variables* combine categorical and numerical inputs as standard domain-specific descriptors are often unavailable.

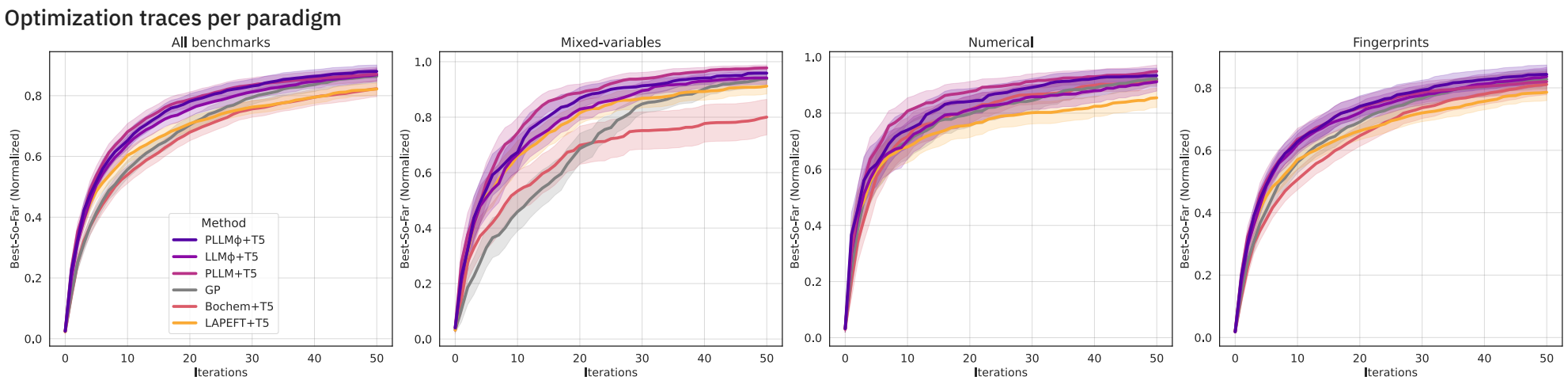

Fig. S7 Best-so-far traces by optimization paradigm. We report normalized best-so-far objective as a function of BO iteration, averaged across benchmarks within each group (shaded regions indicate 95% confidence intervals across random seeds), aggregated by optimization paradigm (All, Mixed-variables, Numerical, Fingerprints). Across paradigms, GOLLuM variants consistently improve optimization speed and final performance.

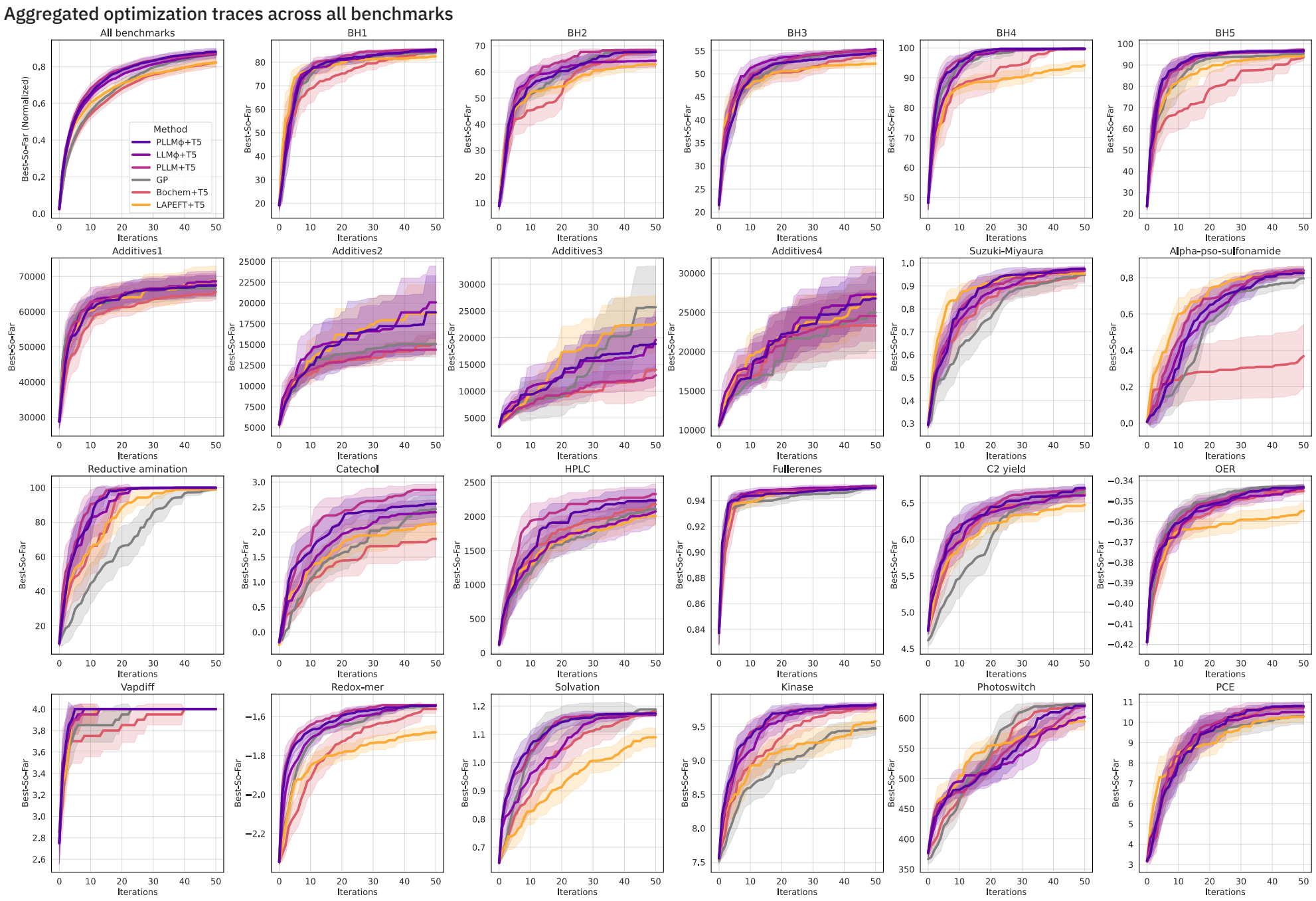

Fig. S8 Per-benchmark optimization traces (best-so-far). For each benchmark, we plot the best-so-far objective value achieved by each method as a function of BO iteration (higher is better). Curves are averaged over random seeds while benchmarks are ordered by chemistry domain following Table S5.3.

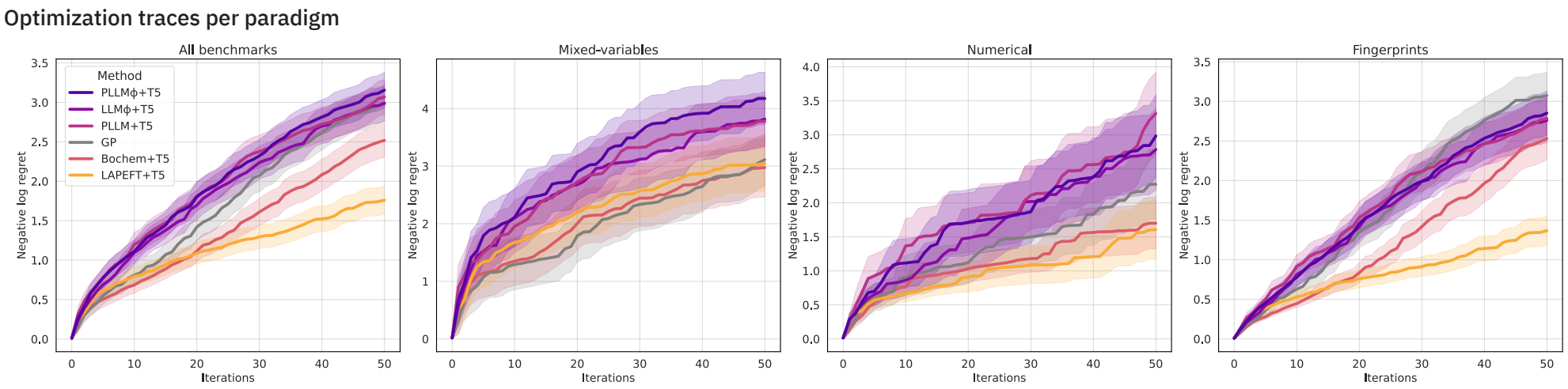

Fig. S9 Optimization traces via negative log regret, aggregated by optimization paradigm (higher is better). We report optimization progress as negative log regret, $-\log_{10}(r_t)$ with $r_t = \max\left(\epsilon, \frac{f_{\max}-f_t^{\text{best}}}{f_{\max}-f_0^{\text{best}}}\right)$, where f_t^{best} is the best-so-far objective at iteration t, f_0^{best} is the best value after initialization (baseline-aligned), and $f_{\max}$ is the best observed value in the full benchmark dataset. We set $\epsilon = 10^{-6}$ for numerical stability; higher values indicate lower regret (closer to the optimum). Curves are averaged across benchmarks within each group and across random seeds; shaded regions denote 95% confidence intervals. Aggregation by optimization paradigm (All benchmarks, Mixed-variables, Numerical, Fingerprints). Overall, GOLLuM variants reduce regret faster and achieve better final performance, with larger gains on mixed-variable and numerical domains.

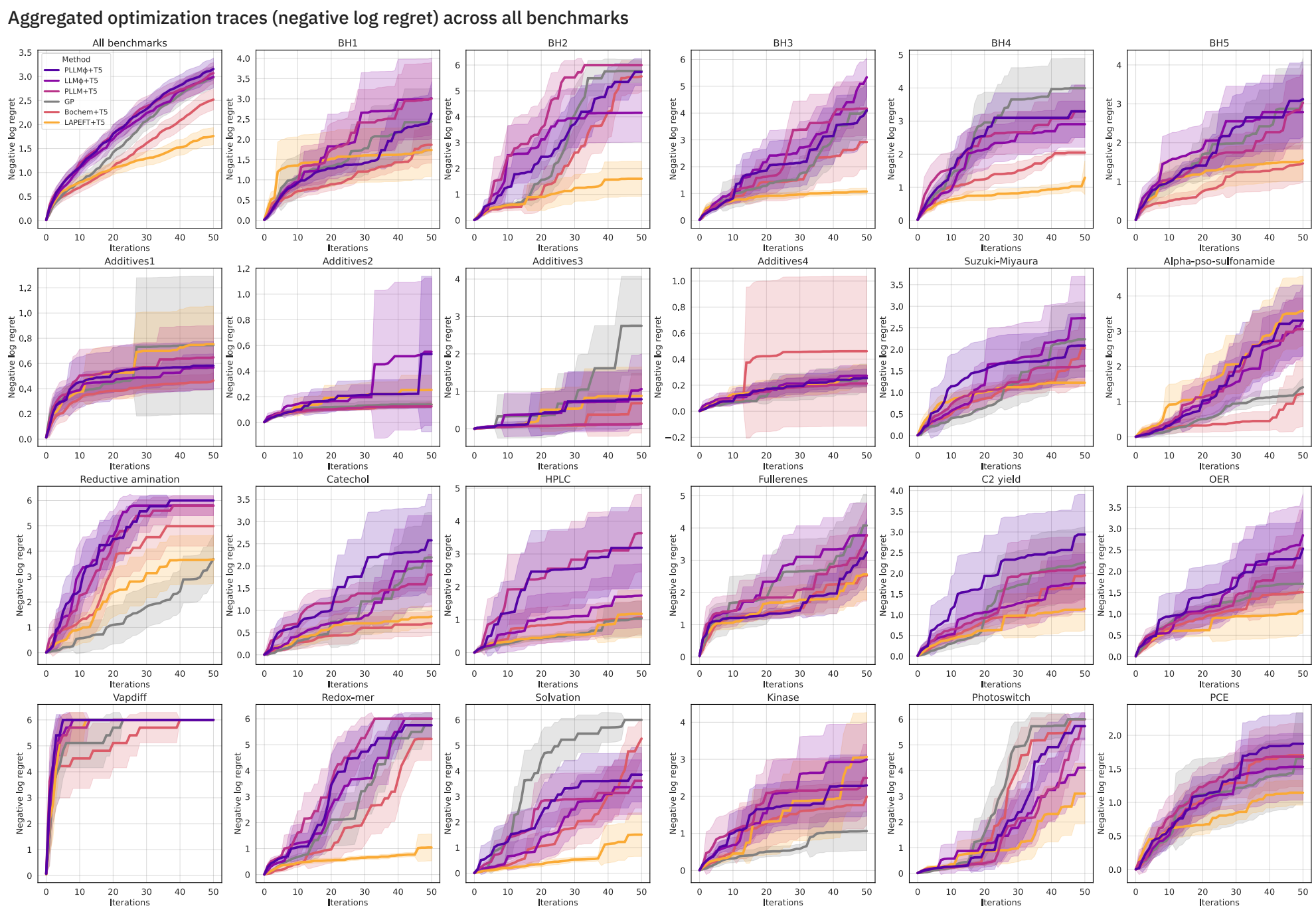

Fig. S10 Per-benchmark optimization traces (negative log regret). For each benchmark, we report the negative log regret as a function of BO iteration, where regret at iteration t is defined as $r_t = f^\star - f_t^{\text{best}}$ for maximization (with $f^\star$ taken as the best observed value in the full dataset for that benchmark), and we plot $-\log(r_t + \epsilon)$ with a small ϵ for numerical stability. Higher values correspond to lower regret (better performance). Curves are averaged over random seeds and benchmarks are ordered by chemistry domain following Table S5.3.

S5.4 LLM pretraining and input format influence

A natural question is whether domain-specific pretraining provides an advantage for chemical optimization. Chemistry-specialized language models such as T5Chem [52] have been pretrained on large corpora of chemical data including SMILES strings and reaction templates. To evaluate whether this specialized pretraining translates to better optimization performance, we compare general-purpose T5 [41] against T5Chem [52] across two input formats: simplified experimental procedures (natural language) and reaction SMILES (the format T5Chem was explicitly pretrained on).

Figure S11 shows optimization performance across five Buchwald-Hartwig reactions using fixed embeddings and GOLLuM updates. With GOLLuM, General-purpose T5 consistently matches or exceeds T5Chem performance, even when T5Chem is provided with SMILES input—the exact format it was pretrained on. T5Chem shows strong format dependency, with a 44% improvement when switching from procedures to SMILES (23% → 33% Top-5% coverage), indicating the embeddings reflect pretraining biases rather than task-relevant structure. In contrast, T5 maintains robust performance across both formats (24-26% coverage), suggesting more transferable representations.

This result has two important implications. First, domain-specific pretraining does not guarantee better optimization performance as the ability to encode task-relevant patterns matters more than chemical knowledge per se. Second, format robustness is a desirable property: practitioners should be able to describe experiments naturally without requiring format-specific engineering. Together with our main results showing that finetuned general-purpose T5 outperforms specialized descriptors, these findings suggest that task-specific adaptation through GP marginal likelihood is more important than domain-specific pretraining.

S5.5 Chemical optimization as chocolate making

While we demonstrate that pretrained chemical knowledge plays a lesser role in our approach, here we further evaluate whether GOLLuM works when the data is completely relabeled. To test the model's ability to recover meaningful patterns from structure alone, we convert the Buchwald-Hartwig dataset into a chocolate design task by renaming chemical components with ingredients from a fictional chocolate factory. Aryl halides become cocoa origins (iodide → Madagascar Criollo, bromide → Ecuadorian Arriba, chloride → Venezuelan Porcelana), while ligands, bases, and additives become processing methods (Stone Ground, Swiss Conched, Dutch Processed), milk types (whole, almond, oat), and spices (Cayenne Chili, Manuka Honey, Himalayan Sea Salt

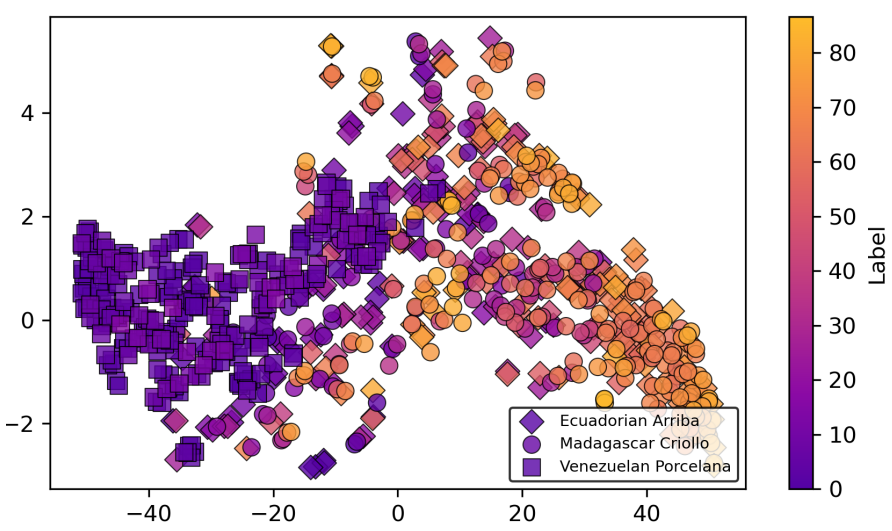

Fig. S12 Pattern discovery without domain knowledge. Buchwald-Hartwig reactions relabeled as chocolate design (iodide → Madagascar Criollo, bromide → Ecuadorian Arriba, chloride → Venezuelan Porcelana). Despite training only on optimization feedback, high-yielding ingredient combinations cluster together, demonstrating that GOLLuM recovers genuine structure-property patterns rather than memorizing chemical priors.

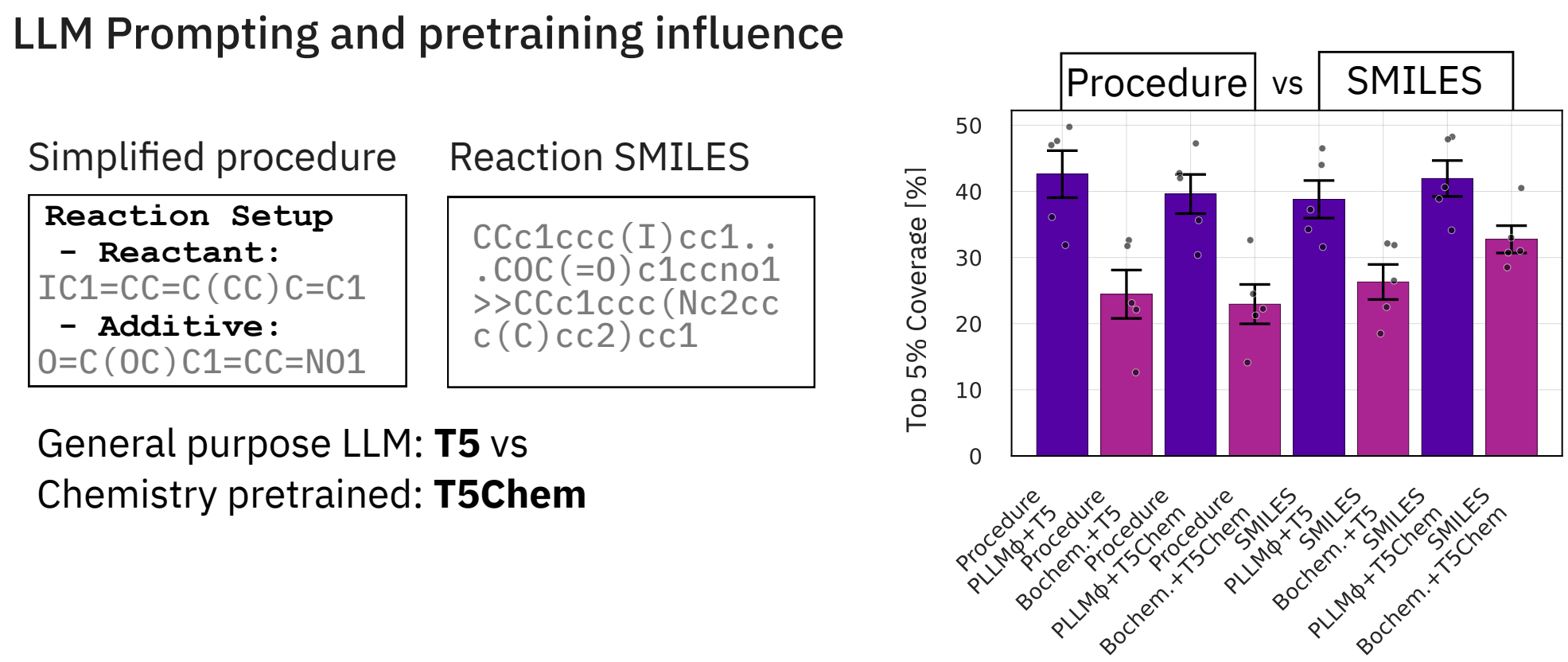

Fig. S11 Influence of prompt formatting and LLM pretraining on optimization performance in Buchwald-Hartwig reactions. We compare two input formats (simplified experimental procedures and reaction SMILES) across general-purpose (T5 [41]) and chemistry-pretrained (T5Chem [52]) language models. Performance remains robust across formats, with general-purpose T5 matching or exceeding chemistry-pretrained T5Chem even on SMILES inputs, the format T5Chem was pretrained on. This result suggests that task-specific adaptation through GP likelihood matters more than domain-specific pretraining. Bar heights show the mean across the five Buchwald-Hartwig benchmarks (BH1–BH5); overlaid dots are the per-benchmark means ($n = 5$) and error bars the standard error of the mean across benchmarks (20 seeds per benchmark).

among others), respectively. Although these substitutions remove all chemical information, the same clustering behavior emerges: chocolates with "high-yielding" cocoa (Madagascar Criollo) group together, while those with "low-performing" cocoa (Venezuelan Porcelana) separate. This demonstrates that GOLLuM discovers genuine patterns from the optimization signal, rather than memorizing associations from pretraining.

S5.6 Structure vs fit and BO performance

Selecting suitable priors is one of the core challenges in BO, especially when limited information is available about the underlying objective function. The first modeling choice in BO is how to represent the input design space. In chemistry, this space can be expressed in various ways, such as SMILES strings, reaction templates, or molecular fingerprints. Often, this representation is predetermined by the constraints of the problem setting, such as one-hot encodings in combinatorial screens. However, the choice of representation imposes downstream consequences on other components of the BO pipeline.

Surrogate models, which map the input $\mathbf{x}$ to the output y in a probabilistic manner, come with their own inductive biases. GPs rely on a kernel function to define similarity between points. The choice of kernel encodes assumptions about smoothness, differentiability, and the geometry of the function to be modeled. For example, the Tanimoto kernel [48] might be better suited for binary fingerprint inputs, while Matérn kernels are broadly applicable to continuous Euclidean representations. In our study, we fix the surrogate kernel to Matérn 5/2 due to its balance between smoothness and flexibility, and its prevalence in chemical BO applications. All related analysis in

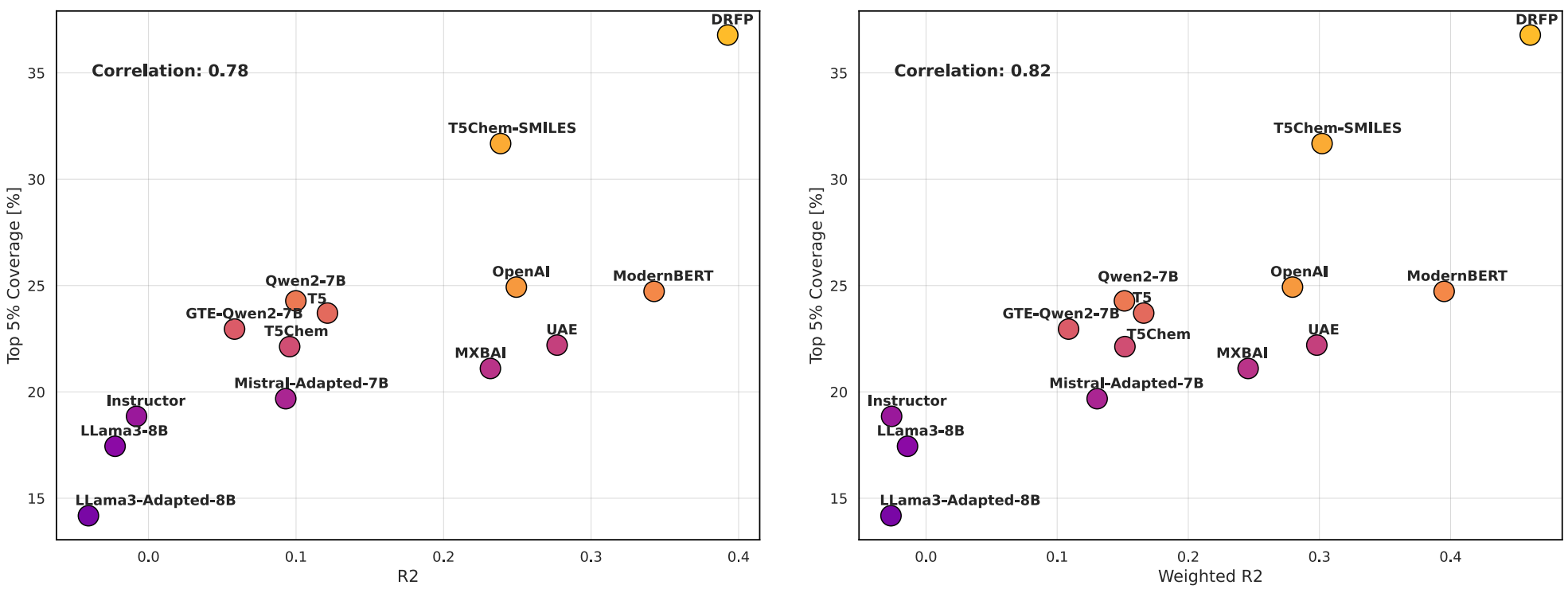

Fig. S13 We compare (left) R^2 over the entire design space, (right) weighted R^2 that upweights points in the top 5% (using a 3:1 weighting scheme). While fit alone is informative ($r = 0.78$ unweighted, $r = 0.82$ weighted), the smoothness metric based on normalized lengthscale achieves a stronger correlation with BO success ($r = 0.92$, main text Figure 3), highlighting the importance of representation-structure alignment.

this section is, therefore, built on the basis of this kernel. Future work may explore kernel-specific behaviors in structure/fit alignment. With the selected design space representation and the surrogate kernel, we are left with a set of assumptions about the structure of the objective function itself. If these assumptions are misspecified – e.g., if the representation induces a geometry not aligned with the kernel – then BO performance can degrade.

We now analyze how the choice of data representation impacts BO performance under a fixed surrogate model and acquisition function. To do so, we introduce a single normalized smoothness metric: the ratio between the GP's learned lengthscale and the average pairwise distance in the embedding space. This metric reflects how far the GP generalizes relative to the data distribution, serving as a proxy for the compatibility between the representation space and the kernel's inductive bias.

We observe a strong correlation ($r = 0.92$) between this normalized lengthscale and BO performance (Main paper, Fig. 3c), suggesting that representations that allow the GP to maintain broader, smoother fits tend to support more successful optimization. A higher ratio indicates that the GP can generalize over broader regions while still resolving performance differences, ultimately leading to better acquisition decisions. This trend is consistent with the intuition that smoother fits – enabled by coherent, well-structured representation spaces – support more principled exploration and reduce overfitting to local noise.

We further show that while standard and weighted R^2 measures correlate with BO performance, their predictive power is consistently lower than the normalized smoothness metric. This supports the view that while accurate fit helps, smooth fits – enabled by representations that align well with the GP kernel – are even more important for effective acquisition. This finding motivates our proposed deep kernel learning approach. By jointly training the LLM and GP via marginal likelihood, we allow the representation to adapt to the GP's inductive assumptions, resulting in

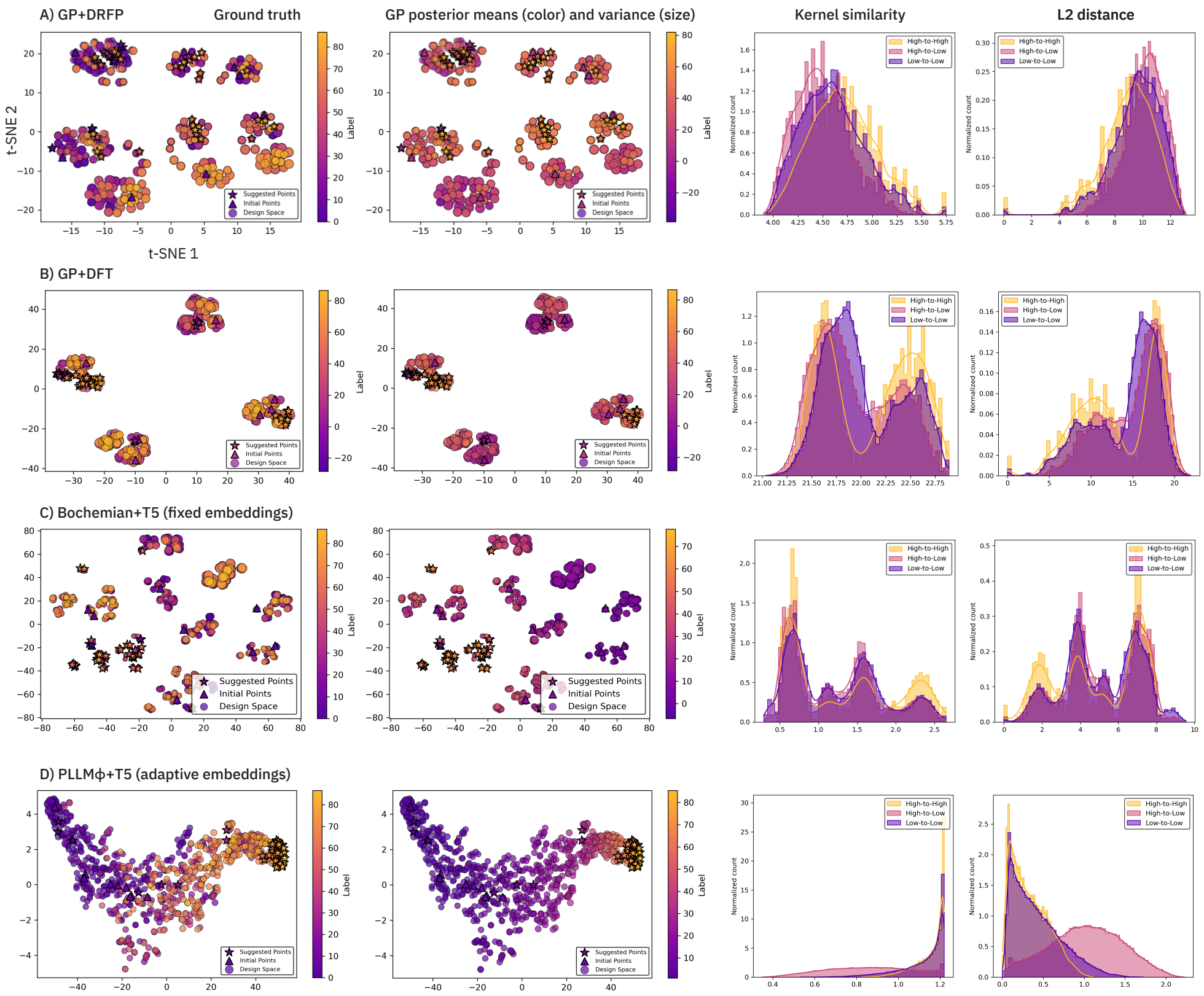

Fig. S14 Visual analysis of latent space structure, GP behavior, and similarity metrics across different representations. We compare four models: (A) GP+DRFP, the best-performing fixed-feature baseline; (B) GP+DFT, physics-based density functional theory (DFT) features [30], commonly used in traditional BO; (C) BoChemian+T5, using frozen LLM embeddings from natural language templates; and (D) PLLMϕ+T5, our best adaptive embedding model. The left two columns visualize the latent space with ground truth (left) and GP posterior mean/variance (middle) colored by yield. We mark the suggested and initial points. The right two columns show pairwise kernel similarities and L2 distances for high–high, high–low, and low–low yielding regions. DRFP exhibits mild structural organization even in its fixed feature space, contributing to strong performance. T5 without finetuning lacks this structure, while PLLMϕ+T5 learns a highly structured latent space, enabling smoother fits and more effective acquisition decisions.

smoother surrogate fits and more structured latent spaces, as shown in Figure S14. This structure ultimately enables better optimization.

We also observe that the best-performing fixed-feature baseline (DRFP) already exhibits relatively structured embedding space, reflected in clear pairwise L2 and kernel similarity histograms. In contrast, the fixed T5 model produces less organized latent structure. With our adaptive method (PLLMϕ+T5), however, the latent space becomes substantially more structured, resulting in smoother GP fits and better-aligned similarity distributions. This outcome confirms the key role of aligning learned representations with the GP kernel's inductive bias.

S5.7 Predictive accuracy and uncertainty calibration

The previous section demonstrated that representation geometry and the smoothness ratio $\ell/\overline{d}$ strongly predict BO performance. Here we complement this geometric analysis by directly evaluating the surrogate model's predictive quality and uncertainty calibration.

The effectiveness of BO depends on both accurate predictions and well-calibrated uncertainty estimates. Accurate predictions help identify promising regions, while calibrated uncertainty guides exploration by quantifying confidence in those predictions. While the main text focuses on optimization performance (Top-5% coverage, sample efficiency), here we report the coefficient of determination (R^2) measuring predictive fit, and the negative log predictive density (NLPD) assessing probabilistic calibration.

**Model**	$R^2 \uparrow$	NLPD $\downarrow$
PLLMΦ+T5 (ours)	0.6 ± 0.1	6.9 ± 1.6
PLLMΦ+T5Chem (ours)	0.6 ± 0.1	8.1 ± 1.5
GP+DRFP	0.4 ± 0.3	4.9 ± 5.0
Bochem.+T5	0.3 ± 0.2	38.3 ± 63.3
Bochem.+T5Chem	0.5 ± 0.1	49.2 ± 62.3
LAPEFT+T5	0.4 ± 0.1	103.2 ± 203.4
LAPEFT+T5Chem	0.5 ± 0.1	357.9 ± 1016.6

Table S5 Regression and uncertainty quantification metrics on Buchwald-Hartwig reactions. We evaluate predictive capabilities by the coefficient of determination (R^2, higher is better), and uncertainty calibration by the negative log predictive density (NLPD, lower is better). We report mean and standard deviation values, calculated over 20 independent runs with 60 training points each, using the remaining data for evaluation. The results show that GOLLuM variants (PLLMϕ) achieve high predictive accuracy while maintaining strong uncertainty calibration.

GOLLuM variants achieve both high predictive accuracy ($R^2 = 0.6$) and well-calibrated uncertainty (NLPD = 6.9-8.1), substantially outperforming baselines on both metrics. BoChemian, which uses fixed LLM embeddings, shows poor calibration (NLPD = 38-49) despite moderate accuracy, while LAPEFT exhibits severe miscalibration (NLPD $>$ 100) due to its post-hoc Laplace approximation applied after supervised finetuning. The GP+DRFP baseline achieves reasonable calibration on these descriptor-rich reactions (NLPD = 4.9) but lower accuracy ($R^2 = 0.4$) with high variance across tasks. Crucially, the GP's calibration is contingent on the availability of a strong domain descriptor. Across the full benchmark suite (Table S9), on descriptor-poor tasks where the GP falls back to numerical parameters (e.g., HPLC, Vapdiff, C2 yield) its held-out NLPD reaches 10^3–10^5, whereas PLLMϕ+T5's worst case across all tasks remains $\approx$34. GOLLuM therefore attains the lowest mean NLPD across the suite, with no catastrophic calibration failure and without requiring any task-specific descriptor.

These results highlight the importance of joint training through the GP marginal likelihood. By optimizing the LLM and GP together, GOLLuM learns representations that support both accurate predictions and calibrated uncertainty, an essential property for reliable sequential decision-making.

S5.8 Which GOLLuM variant to use?

GOLLuM reports three variants that differ in which parameters are trained through the GP marginal likelihood: a projection on frozen embeddings (PLLM), LoRA finetuning of the LLM (LLMϕ), or both (PLLMϕ). Here we compare them and ask whether the gains come from adapting the LLM representation itself or from adding trainable capacity on top of fixed embeddings. Additionally, we use a parameter-matched capacity control to isolate the LoRA influence, and close with a practical recommendation.

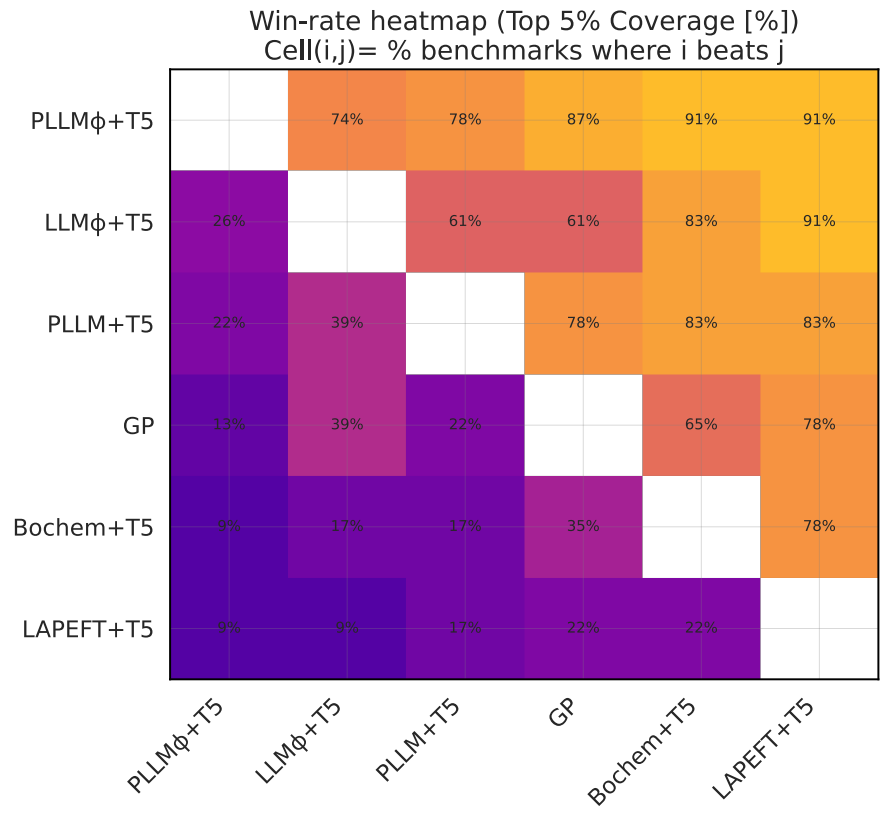

Fig. S15 Win-rate heatmap for Top 5% Coverage Pairwise win-rate heatmap comparing methods on Top 5% Coverage across 23 benchmarks. Each cell (i,j) represents the fraction of benchmarks where row method i achieves higher coverage than column method j. GOLLuM variants (PLLMϕ+T5, LLMϕ+T5, PLLM+T5) consistently outperform baselines, with PLLMϕ+T5 achieving 87-91% win rates against GP and other LLM-BO baselines.

Every deep-kernel variant beats static embeddings.

We compare the static-embedding GP (Bochem+T5) against three deep-kernel variants that differ only in *which* parameters are trained: a linear projection on frozen embeddings (PLLM+T5), LoRA finetuning of the LLM with no projection (LLMϕ+T5), and both together (PLLMϕ+T5).

Crucially, LLMϕ+T5 (which has *no projection layer* and isolates the marginal-likelihood gradients into the LLM) improves over static embeddings by +8.7 pp ($p = 3.7 \times 10^{-5}$), comparable to and numerically larger than the projection-only PLLM+T5 (+6.9 pp). The three variants however, lie within $\sim$2.8 pp of one another: LLMϕ vs PLLM (+1.7 pp, $p = 0.18$) and PLLMϕ vs LLMϕ (+1.1 pp, $p = 0.17$) are not significant, while PLLMϕ exceeds PLLM by a modest but significant +2.8 pp ($p = 0.004$). We therefore frame the contribution as the LLM-based deep kernel rather than any single component.

The gain is not explained by trainable capacity.

To test whether the advantage of PLLMϕ simply reflects more trainable parameters, we add a parameter-matched random MLP on the frozen embeddings ($768 \to 260 \to 64$; 216,644 trainable parameters, matching PLLMϕ's LoRA+projection budget of 216,640), identical to PLLM in all else (DeepGP, Expected Improvement, same learning rate/optimizer/seeds). The capacity-matched MLP is statistically indistinguishable from the linear projection (MLP vs PLLM: $\Delta = -0.2$ pp, $p = 0.38$) and remains

Variant	Trained parameters	Coverage	vs static	Wilcoxon p
Bochem+T5	none (static embeddings + GP)	26.5	—	—
PLLM+T5	projection only	33.5	+6.9	1.7×10^{-4}
LLMϕ+T5	LoRA only (no projection)	35.2	+8.7	3.7×10^{-5}
PLLMϕ+T5	LoRA + projection	36.3	+9.8	1.2×10^{-6}

Table S6 Deep-kernel variants versus static embeddings (Top-5% coverage [%], 50 iterations, 20 seeds; two-sided paired Wilcoxon signed-rank test across 23 benchmarks). Every variant that trains an LLM-based representation through the GP marginal likelihood significantly exceeds the fixed-embedding GP.

significantly below the finetuned model (MLP vs PLLMϕ: $\Delta = -3.1$ pp, $p = 1.6 \times 10^{-8}$). Added capacity on a fixed representation thus does not recover the benefit. Adapting the representation itself under a low-rank, pretrained-anchored constraint does.

Practical recommendation.

The control rules out "more trainable parameters" as the explanation, though we do not claim to isolate the exact source of the with-versus-without-ϕ difference. In practice we recommend:

- **PLLMϕ** as the default when adapting to a new optimization task: it provides the most degrees of freedom to reshape the representation through the GP marginal likelihood and is the strongest variant in aggregate;
- **PLLM** as a strong, substantially cheaper alternative when LoRA finetuning (or access to model weights) is prohibitive, since it recovers most of the benefit (both far exceed static embeddings);
- **LLMϕ** when one prefers to avoid additional projection hyperparameters.

S5.9 BO results per representation type

With the BO traces in Figure S17 we show aggregated results per LLM or chemistry-related representation types. We observe that performance varies in different BH reactions, with no representation consistently outperforming others across all tasks – including chemistry-specialized ones. All LLM types, however, show similar distributions of suggested point evaluations. In comparison to other LLM architectures, encoder-based models tend to achieve higher R^2 values during optimization. However, the improved function approximation does not necessarily translate to better BO performance, as modeling the function and identifying its optimal points are two fundamentally distinct, though complementary, objectives.

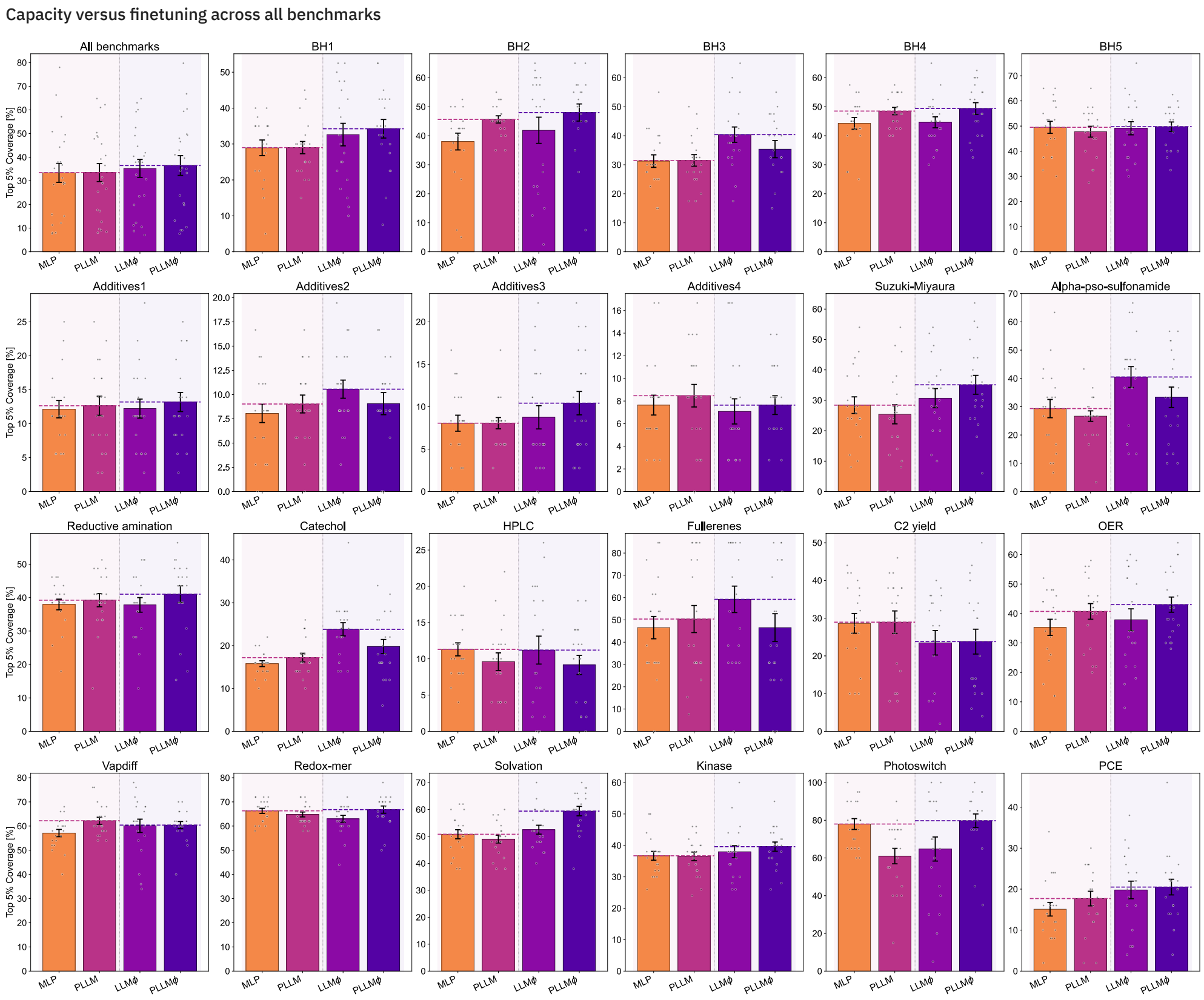

Fig. S16 Capacity versus finetuning. Top-5% coverage [%] at 50 iterations (mean ± SEM, 20 seeds) for the projection-embedding family {MLP+T5 (parameter-matched random MLP), PLLM+T5 (linear projection)} and the ϕ-finetuned family {LLMϕ+T5 (LoRA, no projection), PLLMϕ+T5 (LoRA + projection)}, per benchmark plus an equal-weight average. Overlaid dots show the individual data points underlying each bar (per-benchmark panels: the 20 seed runs, $n = 20$; the equal-weight average panel: the 23 per-benchmark means, $n = 23$), with error bars giving the standard error of the mean over those points. The pink dashed line marks the best of the projection pair (MLP, PLLM); the purple dashed line marks the best of the finetuned pair (LLMϕ, PLLMϕ); shading distinguishes the two families. The capacity-matched MLP tracks the linear PLLM ($\Delta = -0.2$ pp, $p = 0.38$) and stays below the ϕ family; the ϕ-envelope exceeds the projection-envelope on 19/23 tasks (mean +3.0 pp, $p = 3.2 \times 10^{-4}$).

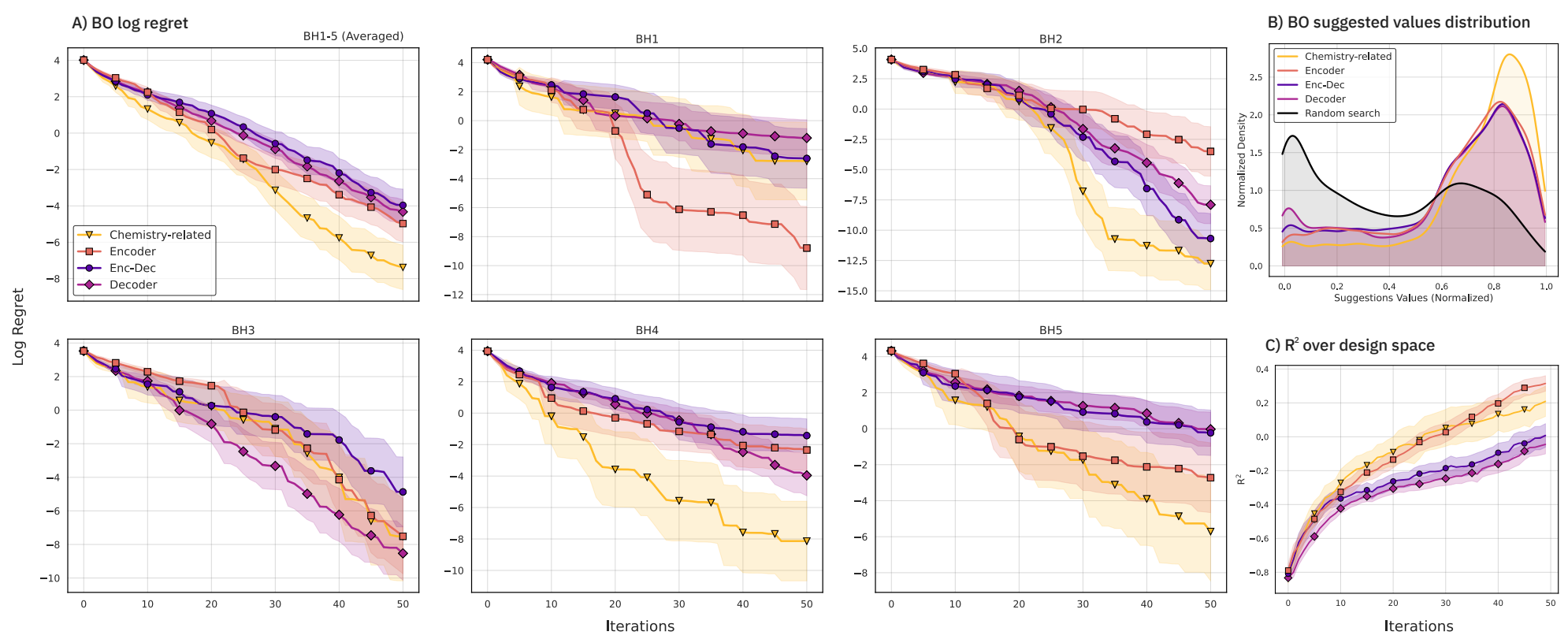

Fig. S17 BO metrics per LLM type. A) We show optimization paths for all BH reactions (BH1-5 Averaged) across different LLM types (Encoder only, Encoder-Decoder, Decoder only) and chemistry-related representations (DRFP, T5Chem-SMILES) together with optimization results on individual reactions (BH1-BH5). B) Distribution of evaluated suggestions generated throughout the entire optimization process (50 iterations) for 20 seed runs and all BH reactions. C) R^2 scores per LLM type over the evolving design space, averaged across all BH reactions.

S6 Reproducibility

S6.1 BO Initialization

Bayesian optimization is in practice initialized with a small number of pre-existing datapoints, either from prior experimentation or simulation. In reaction optimization, for example, these initial points often correspond to unsuccessful or low-yielding reactions. In scientific discovery settings, this setup is not only realistic but expected – optimization typically begins from sparse and suboptimal conditions, with the goal of efficiently identifying high-performing regions.

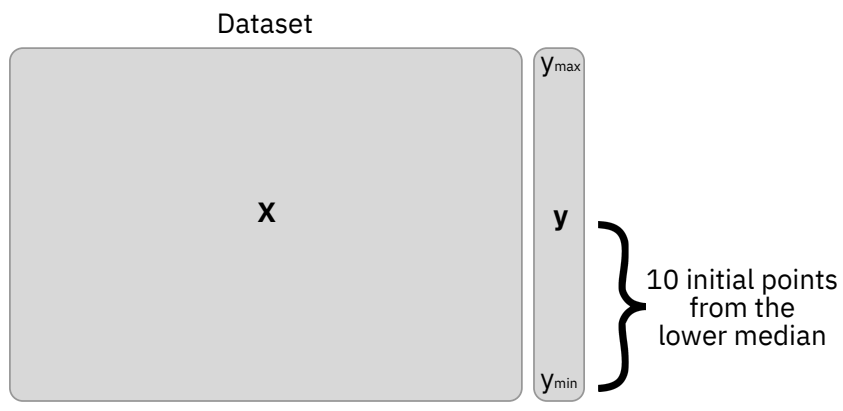

Fig. S18 Illustrative example of initial data selection. For all benchmark datasets, we constrain the selection of initial points for BO to the lower median points based on the objective values. This initialization strategy better reflects real-world scenarios where optimization starts from suboptimal conditions.

This starting point contrasts with scenarios where good conditions are already known, in which case optimization reduces to local exploitation rather than global search. Reaching high-yielding conditions from poor initial data is substantially more challenging and better reflects real-world discovery pipelines. Compounding this difficulty is the negative bias in the scientific literature: failed or low-yield experiments are rarely reported, making published datasets inherently skewed toward successful outcomes [70].

To simulate this setting, we initialize the BO algorithm (Algorithm 1) with 10 points sampled from the lower median range of the objective value distribution. Figure S18 illustrates this strategy. For each benchmark dataset, the candidate pool is first sorted by objective value, and initial points are randomly selected from those with values below the median. This initialization requires the model to reason under uncertainty and efficiently navigate toward high-performing regions with minimal prior knowledge.

S6.2 BO setup

Following the initialization, we run the BO loop for 50 iterations with batch size 1. We repeat each experiment configuration for 20 times with different seed values (1-20) to obtain robust performance metrics. Our choice for the GP kernel is Matérn-5/2, based on its demonstrated effectiveness on both continuous and discrete design spaces [13]. For balancing the exploration and exploitation we employ the expected improvement acquisition function.

S6.3 Implementation details

Surrogate Models

We implement both fixed-feature and finetuned surrogate models as subclasses of `SingleTaskGP` from `botorch` [71]. For fixed-feature GPs, the inputs are LLM embeddings, chemistry-related representations (fingerprints, DRFP) or default parameters (one-hot encoded categorical variables, numerical values), while we learn the GP kernel

hyperparameters $\theta = \{\ell, \sigma^2, \sigma_n^2, c\}$ by maximizing the marginal likelihood using the L-BFGS-B optimizer provided by BoTorch's `fit_gpytorch_mll` routine. We employ the Matérn-5/2 kernel [72] with initialization: $\ell = 1.0$, $\sigma^2 = 1.0$, and $\sigma_n^2 = 1.0 \times 10^{-4}$. The optimizer runs with multiple restarts, as part of BoTorch's default behavior.

For deep kernel GPs, we extend the surrogate to jointly optimize both GP and LLM parameters. We build a custom `DeepGP` class that incorporates a finetuning model (PEFT adapter and/or projection head) jointly trained via AdamW [73]. We optimize GP and LLM parameters using separate learning rates (2×10^{-1} for GP, 2×10^{-3} for LLM) with a shared weight decay of 1×10^{-3}. We apply gradient clipping with a max norm of 1.0 and decay the learning rates using a StepLR scheduler with a decay factor of 0.95.

PEFT Configuration

We insert LoRA adapters into the top 25% of linear layers, using the following configuration: rank $r = 4$, $\alpha = 16$, no bias updates and dropout of 0.2.

Projection Layer

We define the projection layer as:

$$\mathbf{z} = \mathrm{ELU}(\mathrm{Dropout}(W\mathbf{x} + b)), \tag{6}$$

where $W \in \mathbb{R}^{d \times 64}$ and input d is model-dependent. We initialize the parameters of this model via Xavier uniform initialization.

Featurization

We extract LLM embeddings from Hugging Face models using their default tokenizers and truncating the input to maximum of 512 tokens. Pooling strategies depend on the model architecture: CLS token for encoder-based models, last-token for decoder-only models, and mean-pooling for encoder-decoder models.

Training setup

Fixed-feature models can be run on CPU or a single GPU (e.g., RTX 3090 with 24GB VRAM). Finetuned experiments involving large LLMs (e.g., PLLMϕ+Qwen2-7B) are run on NVIDIA H100 GPU and 96GB RAM. Lightweight models (e.g., PLLMϕ+T5, PLLMϕ+ModernBERT) are trainable on local hardware, with under 300k trainable parameters in total (e.g., 230k out of 149M for ModernBERT, 165k out of 109M for T5; less than 0.2% of model weights updated).

Tracking and seeding

All experiments are seeded using `seed_everything` from `pytorch_lighting` [74] with values 1-20. Acquisition optimization is deterministic over the candidate pool. We do not augment the data or use any stochastic featurization. We use `wandb` [75] for tracking experiments, managing seeds, logging losses, metrics, learning rates, images, and running configuration sweeps.

Latent-space visualization

The two-dimensional latent-space panels (main text Figs. 2 and 4c, and SI Fig. S14) are t-SNE [76] projections of the LLM embeddings (scikit-learn implementation), computed with perplexity 50, learning rate 200, 2000 iterations, early exaggeration 6, `n_iter_without_progress` 300, Euclidean distance metric, and random seed 42; initialization uses the scikit-learn default. This reduction is used purely for visualization; all quantitative analyses operate on the full-dimensional embeddings.

LLMs as direct optimizers

To assess off-the-shelf LLMs as prompt-based optimizers (main text Figure 5c), we prompted a range of recent proprietary and open-weight models to propose Buchwald-Hartwig reaction conditions given the parameter space, the optimization goal, and the history of previously evaluated experiments. The models evaluated were GPT-5 (`gpt-5-2025-08-07`) and GPT-5-mini (`gpt-5-mini-2025-08-07`) from OpenAI; Claude Sonnet 4 (`claude-sonnet-4-20250514`) and Claude 3.5 Haiku (`claude-3-5-haiku`) from Anthropic; Gemini 2.5 Pro and Gemini 2.5 Flash from Google; and the open-weight Qwen2-7B-Instruct and Qwen3-235B, each accessed through its respective hosted API with the provider's default sampling settings. Each model was queried as a chat agent: a system prompt enumerated the categorical and continuous parameter space and required a JSON-formatted suggestion, and after every Invalid or Duplicate proposal the agent received corrective feedback and was re-queried, up to a budget of six model calls per requested suggestion. For each of the five products we ran 10 independent seeds (limited by API cost), each cold-started from 10 initial below-median reactions, and queried the model repeatedly until it returned a quota of 50 unique, in-space suggestions. Every raw response was categorized as *Valid*, *Duplicate* (already proposed within the same run), *Invalid* (outside the parameter space, e.g. hallucinated reactants), or *LLM failure* (unparsable output, timeout, or premature termination). The response-type statistics (main text Figure 5c, middle) aggregate all queries issued to reach the quota across the 50 runs (5 products $\times$ 10 seeds) per model (>2,500 queries per model), and the optimization comparison (main text Figure 5c, right) reports Top-5% coverage over the resulting in-space suggestions.

S7 Supporting tables

Representation	Top 5% Coverage [%]
DRFP	**37.750 ± 13.983**
T5Chem-SMILES	32.750 ± 13.728
OpenAI	25.650 ± 12.287
ModernBERT	25.475 ± 10.061
Qwen2-7B	25.150 ± 13.934
T5	24.450 ± 13.005
GTE-Qwen2-7B	23.500 ± 15.288

Representation	Top 5% Coverage [%]
T5Chem	22.950 ± 11.571
UAE	22.800 ± 13.144
MXBAI	21.800 ± 14.733
Mistral-Adapted-7B	20.350 ± 13.046
Instructor	19.300 ± 10.935
LLama3-8B	18.056 ± 12.745
LLama3-Adapted-8B	14.625 ± 10.803
Random Search	6.176 ± 3.751

Table S7: BO with fixed LLM features.

Representation	Top 5% Coverage [%]
PLLMϕ+T5	**42.602 ± 13.111**
LLMϕ+T5	42.577 ± 13.817
PLLM+T5	41.075 ± 10.844
PLLMϕ+Qwen2-7B	40.250 ± 11.189
PLLM+Qwen2-7B	39.375 ± 9.850
LLMϕ+Qwen2-7B	38.725 ± 12.780
PLLMϕ+ModernBERT	36.250 ± 14.100
LLMϕ+ModernBERT	35.775 ± 13.618
PLLM+ModernBERT	34.600 ± 10.805
PLLM+OpenAI	33.525 ± 11.791
OpenAI	25.650 ± 12.287
ModernBERT	25.475 ± 10.061
Qwen2-7B	25.150 ± 13.934
T5	24.450 ± 13.005

Table S8: BO results with LLM-based deep kernels.

Benchmark	Method	$R^2 \uparrow$	$NLPD \downarrow$
	PLLMϕ+T5 (ours)	-0.00 ± 0.10	21.19 ± 2.77
	PLLMϕ+T5Chem (ours)	0.07 ± 0.09	27.02 ± 3.93
	GP+DRFP	0.09 ± 0.06	11.34 ± 0.11
ADDITIVES-1	Bochem.+T5	0.14 ± 0.05	11.66 ± 0.92
	Bochem.+T5Chem	0.17 ± 0.09	11.87 ± 1.25

Benchmark	Method	$R^2 \uparrow$	$NLPD \downarrow$
	LAPEFT+T5	-0.15 ± 0.14	723.23 ± 1625.77
	LAPEFT+T5Chem	-0.04 ± 0.13	329.75 ± 566.60
ADDITIVES-2	PLLMϕ+T5 (ours)	-0.22 ± 0.13	25.72 ± 8.98
	PLLMϕ+T5Chem (ours)	-0.11 ± 0.09	36.73 ± 8.29
	GP+DRFP	0.01 ± 0.02	10.00 ± 0.24
	Bochem.+T5	0.02 ± 0.03	10.58 ± 1.56
	Bochem.+T5Chem	0.05 ± 0.03	10.21 ± 0.67
	LAPEFT+T5	-0.15 ± 0.11	392.67 ± 516.44
	LAPEFT+T5Chem	-0.12 ± 0.10	292.60 ± 346.04
ADDITIVES-3	PLLMϕ+T5 (ours)	-0.30 ± 0.31	33.75 ± 15.29
	PLLMϕ+T5Chem (ours)	-0.19 ± 0.21	52.34 ± 28.62
	GP+DRFP	-0.01 ± 0.02	10.36 ± 0.60
	Bochem.+T5	-0.01 ± 0.02	10.50 ± 0.80
	Bochem.+T5Chem	-0.00 ± 0.02	10.48 ± 0.92
	LAPEFT+T5	-0.21 ± 0.16	641.89 ± 1356.60
	LAPEFT+T5Chem	-0.12 ± 0.12	386.27 ± 431.61
ADDITIVES-4	PLLMϕ+T5 (ours)	-0.34 ± 0.23	22.36 ± 4.87
	PLLMϕ+T5Chem (ours)	-0.21 ± 0.14	28.60 ± 9.03
	GP+DRFP	0.01 ± 0.03	10.27 ± 0.13
	Bochem.+T5	0.01 ± 0.05	11.12 ± 2.58
	Bochem.+T5Chem	0.00 ± 0.05	10.83 ± 1.41
	LAPEFT+T5	-0.22 ± 0.15	186.94 ± 177.56
	LAPEFT+T5Chem	-0.14 ± 0.15	389.52 ± 715.90
BH-1	PLLMϕ+T5 (ours)	0.68 ± 0.05	5.57 ± 0.63
	PLLMϕ+T5Chem (ours)	0.69 ± 0.07	7.11 ± 0.90
	GP+DRFP	0.31 ± 0.34	6.14 ± 7.20
	Bochem.+T5	0.14 ± 0.23	19.96 ± 30.81
	Bochem.+T5Chem	0.52 ± 0.14	32.23 ± 25.51
	LAPEFT+T5	0.51 ± 0.11	65.15 ± 83.74
	LAPEFT+T5Chem	0.60 ± 0.04	216.41 ± 276.61
BH-2	PLLMϕ+T5 (ours)	0.65 ± 0.08	5.96 ± 1.00
	PLLMϕ+T5Chem (ours)	0.65 ± 0.09	7.23 ± 1.17
	GP+DRFP	0.50 ± 0.27	4.06 ± 0.77
	Bochem.+T5	0.22 ± 0.22	60.34 ± 102.47
	Bochem.+T5Chem	0.50 ± 0.08	64.15 ± 58.29
	LAPEFT+T5	0.32 ± 0.18	235.69 ± 415.15
	LAPEFT+T5Chem	0.53 ± 0.06	249.48 ± 192.73

Benchmark	Method	$R^2 \uparrow$	$NLPD \downarrow$
BH-3	PLLMϕ+T5 (ours)	0.47 ± 0.13	6.69 ± 1.28
	PLLMϕ+T5Chem (ours)	0.50 ± 0.10	8.34 ± 1.58
	GP+DRFP	0.38 ± 0.30	5.72 ± 8.59
	Bochem.+T5	0.16 ± 0.14	22.18 ± 47.62
	Bochem.+T5Chem	0.41 ± 0.13	50.64 ± 99.46
	LAPEFT+T5	0.27 ± 0.10	71.06 ± 53.91
	LAPEFT+T5Chem	0.40 ± 0.07	350.63 ± 528.16
BH-4	PLLMϕ+T5 (ours)	0.57 ± 0.08	7.86 ± 1.15
	PLLMϕ+T5Chem (ours)	0.59 ± 0.12	8.72 ± 1.47
	GP+DRFP	0.48 ± 0.29	4.13 ± 0.57
	Bochem.+T5	0.46 ± 0.15	39.56 ± 43.63
	Bochem.+T5Chem	0.46 ± 0.13	41.01 ± 54.16
	LAPEFT+T5	0.32 ± 0.09	80.44 ± 89.41
	LAPEFT+T5Chem	0.40 ± 0.08	581.34 ± 2146.82
BH-5	PLLMϕ+T5 (ours)	0.50 ± 0.10	8.48 ± 1.42
	PLLMϕ+T5Chem (ours)	0.53 ± 0.08	9.08 ± 1.48
	GP+DRFP	0.42 ± 0.28	4.31 ± 0.59
	Bochem.+T5	0.33 ± 0.17	49.31 ± 62.92
	Bochem.+T5Chem	0.43 ± 0.10	57.96 ± 51.71
	LAPEFT+T5	0.37 ± 0.11	63.98 ± 44.88
	LAPEFT+T5Chem	0.42 ± 0.10	391.56 ± 543.81
C2 yield	PLLMϕ+T5 (ours)	0.44 ± 0.09	4.83 ± 2.30
	PLLMϕ+T5Chem (ours)	0.42 ± 0.12	4.56 ± 1.73
	GP+Num.Params	0.16 ± 0.09	154792.41 ± 578478.07
	Bochem.+T5	0.41 ± 0.11	0.99 ± 0.94
	Bochem.+T5Chem	0.42 ± 0.14	5.57 ± 18.91
	LAPEFT+T5	0.29 ± 0.09	180.61 ± 220.08
	LAPEFT+T5Chem	0.24 ± 0.09	1196.45 ± 1636.29
HPLC	PLLMϕ+T5 (ours)	-0.13 ± 0.07	20.13 ± 5.14
	PLLMϕ+T5Chem (ours)	-0.21 ± 0.07	20.18 ± 5.66
	GP+Num.Params	-0.04 ± 0.05	1444.58 ± 6420.47
	Bochem.+T5	-0.03 ± 0.03	18.57 ± 14.46
	Bochem.+T5Chem	-0.02 ± 0.03	49.10 ± 66.46
	LAPEFT+T5	-0.22 ± 0.14	229.99 ± 255.04
	LAPEFT+T5Chem	-0.18 ± 0.09	2427.96 ± 8532.05
KINASE	PLLMϕ+T5 (ours)	0.35 ± 0.06	14.55 ± 2.16
	PLLMϕ+T5Chem (ours)	0.41 ± 0.05	12.82 ± 2.09

Benchmark	Method	$R^2 \uparrow$	$NLPD \downarrow$
	GP+FP	-0.02 ± 0.03	1.41 ± 0.11
	Bochem.+T5	0.02 ± 0.11	3.44 ± 6.44
	Bochem.+T5Chem	-0.02 ± 0.02	1.67 ± 0.11
	LAPEFT+T5	0.33 ± 0.07	12.42 ± 3.44
	LAPEFT+T5Chem	0.45 ± 0.04	21.59 ± 5.58
OER	PLLMϕ+T5 (ours)	0.43 ± 0.15	1.95 ± 1.61
	PLLMϕ+T5Chem (ours)	0.42 ± 0.15	0.88 ± 1.31
	GP+Num.Params	0.59 ± 0.04	189.97 ± 218.23
	Bochem.+T5	0.44 ± 0.09	17.26 ± 23.03
	Bochem.+T5Chem	0.44 ± 0.11	154.70 ± 206.00
	LAPEFT+T5	0.47 ± 0.08	27.27 ± 34.99
	LAPEFT+T5Chem	0.47 ± 0.07	174.81 ± 286.01
PCE	PLLMϕ+T5 (ours)	0.25 ± 0.12	16.12 ± 4.78
	PLLMϕ+T5Chem (ours)	0.27 ± 0.11	15.43 ± 4.42
	GP+FP	-0.02 ± 0.02	2.52 ± 0.08
	Bochem.+T5	0.10 ± 0.15	4.51 ± 6.22
	Bochem.+T5Chem	-0.02 ± 0.03	2.57 ± 0.15
	LAPEFT+T5	0.16 ± 0.15	29.69 ± 46.25
	LAPEFT+T5Chem	0.26 ± 0.13	41.33 ± 21.10
PHOTOSWITCH	PLLMϕ+T5 (ours)	0.59 ± 0.12	11.53 ± 2.13
	PLLMϕ+T5Chem (ours)	0.65 ± 0.10	9.86 ± 1.62
	GP+FP	0.57 ± 0.26	5.13 ± 0.58
	Bochem.+T5	0.58 ± 0.07	41238535.40 ± 32024292.47
	Bochem.+T5Chem	0.46 ± 0.28	151181279.47 ± 202238451.62
	LAPEFT+T5	0.52 ± 0.08	7.78 ± 1.20
	LAPEFT+T5Chem	0.64 ± 0.07	18.50 ± 11.90
REDOX-MER	PLLMϕ+T5 (ours)	0.86 ± 0.02	0.48 ± 0.47
	PLLMϕ+T5Chem (ours)	0.89 ± 0.02	-0.02 ± 0.35
	GP+FP	0.73 ± 0.25	-0.51 ± 0.44
	Bochem.+T5	0.85 ± 0.02	19.72 ± 16.26
	Bochem.+T5Chem	0.85 ± 0.20	47.01 ± 40.57
	LAPEFT+T5	0.73 ± 0.06	6.45 ± 2.53
	LAPEFT+T5Chem	0.86 ± 0.03	16.55 ± 6.20
SOLVATION	PLLMϕ+T5 (ours)	0.74 ± 0.03	2.84 ± 0.98
	PLLMϕ+T5Chem (ours)	0.73 ± 0.04	2.86 ± 0.97
	GP+FP	0.77 ± 0.03	-0.36 ± 0.47
	Bochem.+T5	0.79 ± 0.03	77.92 ± 58.50

Benchmark	Method	$R^2 \uparrow$	$NLPD \downarrow$
	Bochem.+T5Chem	0.69 ± 0.30	174.24 ± 192.44
	LAPEFT+T5	0.71 ± 0.05	14.28 ± 20.01
	LAPEFT+T5Chem	0.79 ± 0.02	73.66 ± 38.83
SUZUKI-MIYAURA	PLLMϕ+T5 (ours)	0.10 ± 0.13	8.16 ± 1.45
	PLLMϕ+T5Chem (ours)	0.12 ± 0.13	9.65 ± 2.37
	GP+DRFP	0.29 ± 0.15	1.20 ± 2.57
	Bochem.+T5	0.07 ± 0.08	8.76 ± 28.10
	Bochem.+T5Chem	0.12 ± 0.10	1.11 ± 3.25
	LAPEFT+T5	-0.10 ± 0.18	107.12 ± 105.69
	LAPEFT+T5Chem	0.18 ± 0.09	231.35 ± 181.75
VAPDIFF	PLLMϕ+T5 (ours)	-0.01 ± 0.06	12.40 ± 2.73
	PLLMϕ+T5Chem (ours)	-0.05 ± 0.09	14.25 ± 3.41
	GP+Num.Params	0.12 ± 0.06	608876.27 ± 906151.79
	Bochem.+T5	0.07 ± 0.06	1.69 ± 0.28
	Bochem.+T5Chem	0.08 ± 0.07	1.72 ± 0.46
	LAPEFT+T5	-0.17 ± 0.11	288.12 ± 284.60
	LAPEFT+T5Chem	-0.04 ± 0.08	164.06 ± 142.57

Table S9: Predictive and uncertainty estimates for all benchmark datasets and methods. Each model is trained on 60 points and evaluated on the remaining data, emulating a 10+50 BO iteration setup. This fixed train/validation split ensures fair comparison by avoiding divergence in design sets during BO due to different selection of candidate points during optimization, even when starting with the same initial points. We run 20 repeats and report mean and standard deviation values.

Arch.	Pool	Model	Quant. 95 [cnt]
Enc	CLS	MXBAI	2.70 ± 2.15
		ModernBERT	2.40 ± 2.09
		UAE	2.40 ± 2.11
	Avg	MXBAI	2.30 ± 2.00
		ModernBERT	2.30 ± 1.72
		UAE	2.25 ± 1.86
	Last	MXBAI	1.80 ± 1.82
		ModernBERT	0.25 ± 0.55
		UAE	2.50 ± 2.35
Dec	Avg	LLama3-8B	0.75 ± 1.48
		Qwen2-7B	1.05 ± 2.28
	Last	LLama3-8B	1.55 ± 1.64
		Qwen2-7B	1.65 ± 2.54
Enc-Dec	Avg	T5	1.10 ± 1.41
		T5Chem	1.90 ± 2.05
	Last	T5	1.65 ± 2.48
		T5Chem	1.55 ± 2.91

Table S10 Tokenization influence to sampling from the 5th percentile.

Architecture	Target layers	Target ratio	Deep Kernel + Model	Quantile 95 [cnt]
Chem-related	Top	0.1	PLLMϕ+T5Chem-SMILES	6.60 ± 1.90
		0.25		6.30 ± 3.47
		0.5		8.20 ± 2.94
	Bottom	0.1		7.50 ± 2.51
		0.25		7.40 ± 2.32
		0.5		6.60 ± 3.13
Encoder	Top	0.1	PLLMϕ+ModernBERT	5.50 ± 2.17
		0.25		5.60 ± 2.37
		0.5		5.70 ± 2.83
	Bottom	0.1		4.20 ± 2.44
		0.25		4.50 ± 3.21
		0.5		4.30 ± 2.11
Enc-Dec	Top	0.1	PLLMϕ+T5	4.10 ± 1.91
		0.25		5.40 ± 2.99
		0.5		3.90 ± 3.25
	Bottom	0.1		4.90 ± 2.08
		0.25		5.40 ± 2.55
		0.5		4.90 ± 2.18
Decoder	Top	0.1	PLLMϕ+Qwen2-7B	4.10 ± 2.28
		0.25		4.90 ± 2.88

Table S11 GP-LLM finetuning per LLM types and LoRA layers

References

[1] Jablonka, K.M., Schwaller, P., Ortega-Guerrero, A., Smit, B.: Leveraging large language models for predictive chemistry. Nature Machine Intelligence **6**(2), 161–169 (2024)

[2] Xie, T., Wan, Y., Huang, W., Yin, Z., Liu, Y., Wang, S., Linghu, Q., Kit, C., Grazian, C., Zhang, W., et al.: Darwin series: Domain specific large language models for natural science. arXiv preprint arXiv:2308.13565 (2023)

[3] Ramos, M.C., Michtavy, S.S., Porosoff, M.D., White, A.D.: Bayesian Optimization of Catalysts With In-context Learning (2023)

[4] Nguyen, T., Zhang, Q., Yang, B., Lee, C., Bornschein, J., Perel, S., Chen, Y., Song, X.: Predicting from strings: Language model embeddings for bayesian optimization (2024)

[5] Mahammadli, K., Ertekin, S.: Sequential large language model-based hyper-parameter optimization. arXiv preprint arXiv:2410.20302 (2024)

[6] MacKnight, R., Regio, J.E., Ethier, J.G., Baldwin, L.A., Gomes, G.: Pre-trained knowledge elevates large language models beyond traditional chemical reaction optimizers. arXiv preprint arXiv:2509.00103 (2025)

[7] Ranković, B., Schwaller, P.: Bochemian: Large language model embeddings for bayesian optimization of chemical reactions. In: NeurIPS 2023 Workshop on Adaptive Experimental Design and Active Learning in the Real World (2023)

[8] Kristiadi, A., Strieth-Kalthoff, F., Skreta, M., Poupart, P., Aspuru-Guzik, A., Pleiss, G.: A sober look at LLMs for material discovery: Are they actually good for Bayesian optimization over molecules? In: ICML (2024)

[9] Daxberger, E., Kristiadi, A., Immer, A., Eschenhagen, R., Bauer, M., Hennig, P.: Laplace redux–effortless Bayesian deep learning. In: NeurIPS (2021)

[10] Yang, A.X., Robeyns, M., Wang, X., Aitchison, L.: Bayesian low-rank adaptation for large language models. arXiv preprint arXiv:2308.13111 (2023)

[11] Agarwal, D., Arivazhagan, M.G., Das, R., Swamy, S., Khosla, S., Gangadharaiah, R.: Searching for optimal solutions with llms via bayesian optimization. In: The Thirteenth International Conference on Learning Representations

[12] Cissé, A., Evangelopoulos, X., Gusev, V.V., Cooper, A.I.: Language-based bayesian optimization research assistant (bora). arXiv preprint arXiv:2501.16224 (2025)

[13] Ranković, B., Griffiths, R.-R., Moss, H.B., Schwaller, P.: Bayesian optimisation for additive screening and yield improvements–beyond one-hot encoding. Digital Discovery **3**(4), 654–666 (2024)

[14] Singh, S., Hernández-Lobato, J.M.: Deep kernel learning for reaction outcome prediction and optimization. Communications Chemistry **7**(1), 136 (2024)

[15] Chen, W., Tripp, A., Hernández-Lobato, J.M.: Meta-learning adaptive deep kernel gaussian processes for molecular property prediction. arXiv preprint arXiv:2205.02708 (2022)

[16] Griffiths, R.-R., Hernández-Lobato, J.M.: Constrained bayesian optimization for automatic chemical design using variational autoencoders. Chemical science **11**(2), 577–586 (2020)

[17] Grosnit, A., Tutunov, R., Maraval, A.M., Griffiths, R.-R., Cowen-Rivers, A.I., Yang, L., Zhu, L., Lyu, W., Chen, Z., Wang, J., et al.: High-dimensional bayesian optimisation with variational autoencoders and deep metric learning. arXiv preprint arXiv:2106.03609 (2021)

[18] Ober, S.W., Rasmussen, C.E., Wilk, M.: The promises and pitfalls of deep kernel learning. In: Uncertainty in Artificial Intelligence, pp. 1206–1216 (2021). PMLR

[19] Stanton, S., Maddox, W., Gruver, N., Maffettone, P., Delaney, E., Greenside, P., Wilson, A.G.: Accelerating bayesian optimization for biological sequence design with denoising autoencoders. In: International Conference on Machine Learning, pp. 20459–20478 (2022). PMLR

[20] Frazier, P.I.: A tutorial on bayesian optimization. arXiv preprint arXiv:1807.02811 (2018)

[21] Papenmeier, L., Poloczek, M., Nardi, L.: Understanding high-dimensional bayesian optimization. arXiv preprint arXiv:2502.09198 (2025)

[22] Xu, Z., Wang, H., Phillips, J.M., Zhe, S.: Standard gaussian process is all you need for high-dimensional bayesian optimization. In: The Thirteenth International Conference on Learning Representations (2025). https://openreview.net/forum?id=kX8h23UG6v

[23] Chuang, K.V., Keiser, M.J.: Comment on "predicting reaction performance in C–N cross-coupling using machine learning". Science **362**(6416), 8603 (2018)

[24] Rogers, D., Hahn, M.: Extended-connectivity fingerprints. Journal of Chemical Information and Modeling **50**(5), 742–754 (2010)

[25] Schneider, N., Lowe, D.M., Sayle, R.A., Landrum, G.A.: Development of a novel fingerprint for chemical reactions and its application to large-scale reaction classification and similarity. Journal of Chemical Information and Modeling **55**(1), 39–53 (2015)

[26] Capecchi, A., Probst, D., Reymond, J.-L.: One molecular fingerprint to rule them

all: drugs, biomolecules, and the metabolome. Journal of cheminformatics **12**(1), 1–15 (2020)

[27] Probst, D., Schwaller, P., Reymond, J.-L.: Reaction classification and yield prediction using the differential reaction fingerprint drfp. Digital Discovery **1**(2), 91–97 (2022)

[28] Schwaller, P., Probst, D., Vaucher, A.C., Nair, V.H., Kreutter, D., Laino, T., Reymond, J.-L.: Mapping the space of chemical reactions using attention-based neural networks. Nature Machine Intelligence **3**(2), 144–152 (2021)

[29] Ahneman, D.T., Estrada, J.G., Lin, S., Dreher, S.D., Doyle, A.G.: Predicting reaction performance in C–N cross-coupling using machine learning. Science **360**(6385), 186–190 (2018)

[30] Shields, B.J., Stevens, J., Li, J., Parasram, M., Damani, F., Alvarado, J.I.M., Janey, J.M., Adams, R.P., Doyle, A.G.: Bayesian reaction optimization as a tool for chemical synthesis. Nature **590**(7844), 89–96 (2021)

[31] Jones, D.R., Schonlau, M., Welch, W.J.: Efficient global optimization of expensive black-box functions. Journal of Global optimization **13**, 455–492 (1998)

[32] Jones, D.R.: A taxonomy of global optimization methods based on response surfaces. Journal of global optimization **21**, 345–383 (2001)

[33] Auer, P.: Using confidence bounds for exploitation-exploration trade-offs. Journal of Machine Learning Research **3**(Nov), 397–422 (2002)

[34] Srinivas, N., Krause, A., Kakade, S.M., Seeger, M.: Gaussian process optimization in the bandit setting: No regret and experimental design. arXiv preprint arXiv:0912.3995 (2009)

[35] Thompson, W.R.: On the likelihood that one unknown probability exceeds another in view of the evidence of two samples. Biometrika **25**(3/4), 285–294 (1933)

[36] Wilson, A.G., Hu, Z., Salakhutdinov, R., Xing, E.P.: Deep kernel learning. In: Artificial Intelligence and Statistics, pp. 370–378 (2016). PMLR

[37] Kudo, T., Richardson, J.: Sentencepiece: A simple and language independent subword tokenizer and detokenizer for neural text processing. arXiv preprint arXiv:1808.06226 (2018)

[38] Radford, A., Wu, J., Child, R., Luan, D., Amodei, D., Sutskever, I., *et al.*: Language models are unsupervised multitask learners. OpenAI blog **1**(8), 9 (2019)

[39] Devlin, J., Chang, M.-W., Lee, K., Toutanova, K.: BERT: Pre-training of deep bidirectional transformers for language understanding. arXiv preprint arXiv:1810.04805 (2018)

[40] Bai, J., Bai, S., Chu, Y., Cui, Z., Dang, K., Deng, X., Fan, Y., Ge, W., Han, Y., Huang, F., et al.: Qwen technical report. arXiv preprint arXiv:2309.16609 (2023)

[41] Raffel, C., Shazeer, N., Roberts, A., Lee, K., Narang, S., Matena, M., Zhou, Y., Li, W., Liu, P.J.: Exploring the limits of transfer learning with a unified text-to-text transformer. Journal of Machine Learning Research **21**(140), 1–67 (2020)

[42] Hu, E.J., Shen, Y., Wallis, P., Allen-Zhu, Z., Li, Y., Wang, S., Wang, L., Chen, W., *et al.*: Lora: Low-rank adaptation of large language models. ICLR **1**(2), 3 (2022)

[43] Cereto-Massagué, A., Ojeda, M.J., Valls, C., Mulero, M., Garcia-Vallvé, S., Pujadas, G.: Molecular fingerprint similarity search in virtual screening. Methods **71**, 58–63 (2015)

[44] Anderson, E., Veith, G.D., Weininger, D.: SMILES, a Line Notation and Computerized Interpreter for Chemical Structures. US Environmental Protection Agency, Environmental Research Laboratory, Duluth, MN (1987)

[45] Weininger, D.: SMILES, a chemical language and information system. 1. Introduction to methodology and encoding rules. Journal of Chemical Information and Computer Sciences **28**(1), 31–36 (1988)

[46] Kearnes, S., McCloskey, K., Berndl, M., Pande, V., Riley, P.: Molecular graph convolutions: moving beyond fingerprints. Journal of computer-aided molecular design **30**, 595–608 (2016)

[47] Bannwarth, C., Ehlert, S., Grimme, S.: GFN2-xTB—An accurate and broadly parametrized self-consistent tight-binding quantum chemical method with multipole electrostatics and density-dependent dispersion contributions. Journal of Chemical Theory and Computation **15**(3), 1652–1671 (2019)

[48] Griffiths, R.-R., Klarner, L., Moss, H., Ravuri, A., Truong, S.T., Du, Y., Stanton, S.D., Tom, G., Ranković, B., Jamasb, A.R., Deshwal, A., Schwartz, J., Tripp, A., Kell, G., Frieder, S., Bourached, A., Chan, A.J., Moss, J., Guo, C., Dürholt, J.P., Chaurasia, S., Park, J.W., Strieth-Kalthoff, F., Lee, A., Cheng, B., Aspuru-Guzik, A., Schwaller, P., Tang, J.: GAUCHE: A library for gaussian processes in chemistry. In: Thirty-seventh Conference on Neural Information Processing Systems (2023). https://openreview.net/forum?id=vzrA6uqOis

[49] Warner, B., Chaffin, A., Clavié, B., Weller, O., Hallström, O., Taghadouini, S., Gallagher, A., Biswas, R., Ladhak, F., Aarsen, T., Cooper, N., Adams, G., Howard, J., Poli, I.: Smarter, Better, Faster, Longer: A Modern Bidirectional Encoder for Fast, Memory Efficient, and Long Context Finetuning and Inference (2024). https://arxiv.org/abs/2412.13663

[50] Li, X., Li, J.: Angle-optimized text embeddings. arXiv preprint arXiv:2309.12871 (2023)

[51] Lee, S., Shakir, A., Koenig, D., Lipp, J.: Open Source Strikes Bread - New Fluffy Embeddings Model. https://www.mixedbread.ai/blog/mxbai-embed-large-v1

[52] Christofidellis, D., Giannone, G., Born, J., Winther, O., Laino, T., Manica, M.: Unifying molecular and textual representations via multi-task language modelling. In: Proceedings of the 40th International Conference on Machine Learning. Proceedings of Machine Learning Research, vol. 202, pp. 6140–6157. PMLR, Honolulu, HI, USA (2023). https://proceedings.mlr.press/v202/christofidellis23a.html

[53] Su, H., Kasai, J., Wang, Y., Hu, Y., Ostendorf, M., Yih, W.-t., Smith, N.A., Zettlemoyer, L., Yu, T., et al.: One embedder, any task: Instruction-finetuned text embeddings. arXiv preprint arXiv:2212.09741 (2022)

[54] Radford, A., Narasimhan, K., Salimans, T., Sutskever, I., et al.: Improving language understanding by generative pre-training (2018)

[55] OpenAI: New embedding models and API updates (2024). https://openai.com/index/new-embedding-models-and-api-updates/

[56] Li, Z., Zhang, X., Zhang, Y., Long, D., Xie, P., Zhang, M.: Towards general text embeddings with multi-stage contrastive learning. arXiv preprint arXiv:2308.03281 (2023)

[57] Grattafiori, A., Dubey, A., Jauhri, A., Pandey, A., Kadian, A., Al-Dahle, A., Letman, A., Mathur, A., Schelten, A., Vaughan, A., et al.: The llama 3 herd of models. arXiv preprint arXiv:2407.21783 (2024)

[58] BehnamGhader, P., Adlakha, V., Mosbach, M., Bahdanau, D., Chapados, N., Reddy, S.: Llm2vec: Large language models are secretly powerful text encoders. arXiv preprint arXiv:2404.05961 (2024)

[59] Prieto Kullmer, C.N., Kautzky, J.A., Krska, S.W., Nowak, T., Dreher, S.D., MacMillan, D.W.: Accelerating reaction generality and mechanistic insight through additive mapping. Science **376**(6592), 532–539 (2022)

[60] Perera, D., Tucker, J.W., Brahmbhatt, S., Helal, C.J., Chong, A., Farrell, W., Richardson, P., Sach, N.W.: A platform for automated nanomole-scale reaction screening and micromole-scale synthesis in flow. Science **359**(6374), 429–434 (2018)

[61] Schlama, R., Sin, J.W., Burwood, R.P., Püntener, K., Bigler, R., Schwaller, P.: Swarm intelligence for chemical reaction optimisation. arXiv preprint arXiv:2509.11798 (2025)

[62] Gesmundo, N., Dykstra, K., Douthwaite, J.L., Kao, Y.-T., Zhao, R., Mahjour, B., Ferguson, R., Dreher, S., Sauvagnat, B., Saurí, J., *et al.*: Miniaturization of popular reactions from the medicinal chemists' toolbox for ultrahigh-throughput experimentation. Nature Synthesis **2**(11), 1082–1091 (2023)

[63] Boyne, T., Campos, J.S., Langdon, B.D., Qing, J., Xie, Y., Zhang, S., Tsay, C., Misener, R., Davies, D.W., Jelfs, K.E., et al.: The catechol benchmark: Time-series solvent selection data for few-shot machine learning. arXiv preprint arXiv:2506.07619 (2025)

[64] Häse, F., Aldeghi, M., Hickman, R.J., Roch, L.M., Christensen, M., Liles, E., Hein, J.E., Aspuru-Guzik, A.: Olympus: a benchmarking framework for noisy optimization and experiment planning. Machine Learning: Science and Technology **2**(3), 035021 (2021) https://doi.org/10.1088/2632-2153/abedc8

[65] Walker, B.E., Bannock, J.H., Nightingale, A.M., deMello, J.C.: Tuning reaction products by constrained optimisation. Reaction Chemistry & Engineering **2**(5), 785–798 (2017)

[66] Agarwal, G., Doan, H.A., Robertson, L.A., Zhang, L., Assary, R.S.: Discovery of energy storage molecular materials using quantum chemistry-guided multiobjective bayesian optimization. Chemistry of Materials **33**(20), 8133–8144 (2021) https://doi.org/10.1021/acs.chemmater.1c02040

[67] Graff, D.E., Shakhnovich, E.I., Coley, C.W.: Accelerating high-throughput virtual screening through molecular pool-based active learning. Chemical Science **12**(22), 7866–7881 (2021) https://doi.org/10.1039/d0sc06805e

[68] Griffiths, R.-R., Greenfield, J.L., Thawani, A.R., Jamasb, A.R., Moss, H.B., Bourached, A., Jones, P., McCorkindale, W., Aldrick, A.A., Fuchter, M.J., Lee, A.A.: Data-driven discovery of molecular photoswitches with multioutput gaussian processes. Chemical Science **13**(45), 13541–13551 (2022) https://doi.org/10.1039/d2sc04306h

[69] Lopez, S.A., Pyzer-Knapp, E.O., Simm, G.N., Lutzow, T., Li, K., Seress, L.R., Hachmann, J., Aspuru-Guzik, A.: The harvard organic photovoltaic dataset. Scientific Data **3**(1) (2016) https://doi.org/10.1038/sdata.2016.86

[70] Ioannidis, J.P.: Why most published research findings are false. PLoS medicine **2**(8), 124 (2005)

[71] Balandat, M., Karrer, B., Jiang, D.R., Daulton, S., Letham, B., Wilson, A.G., Bakshy, E.: BoTorch: A Framework for Efficient Monte-Carlo Bayesian Optimization. In: Advances in Neural Information Processing Systems 33 (2020). http://arxiv.org/abs/1910.06403

[72] Gardner, J.R., Pleiss, G., Bindel, D., Weinberger, K.Q., Wilson, A.G.: Gpytorch: Blackbox matrix-matrix gaussian process inference with gpu acceleration. In: Advances in Neural Information Processing Systems (2018)

[73] Loshchilov, I., Hutter, F.: Decoupled weight decay regularization. arXiv preprint arXiv:1711.05101 (2017)

[74] Falcon, W., The PyTorch Lightning team: PyTorch Lightning. https://doi.org/10.5281/zenodo.3828935 . https://github.com/Lightning-AI/lightning

[75] Biewald, L.: Experiment Tracking with Weights and Biases. Software available from wandb.com (2020). https://www.wandb.com/

[76] Maaten, L., Hinton, G.: Visualizing data using t-sne. Journal of Machine Learning Research **9**(11), 2579–2605 (2008)
