# Supplementary material for: Large language models as uncertainty-calibrated optimizers for experimental discovery
Source: Nat Mach Intell. 2026 Aug 28;8(9):1466–77. doi: 10.1038/s42256-026-01283-z (PMC13585549; doi:10.1038/s42256-026-01283-z)
Supplement: Supplementary file 2 [file 42256_2026_1283_MOESM2_ESM.pdf]

nature portfolio

Corresponding author(s): Bojana Ranković, Philippe Schwaller

Last updated by author(s): Jun 10, 2026

Reporting Summary

Nature Portfolio wishes to improve the reproducibility of the work that we publish. This form provides structure for consistency and transparency in reporting. For further information on Nature Portfolio policies, see our Editorial Policies and the Editorial Policy Checklist.

Statistics

For all statistical analyses, confirm that the following items are present in the figure legend, table legend, main text, or Methods section.

n/a	Confirmed	
☐	☒	The exact sample size (*n*) for each experimental group/condition, given as a discrete number and unit of measurement
☐	☒	A statement on whether measurements were taken from distinct samples or whether the same sample was measured repeatedly
☐	☒	The statistical test(s) used AND whether they are one- or two-sided<br>*Only common tests should be described solely by name; describe more complex techniques in the Methods section.*
☒	☐	A description of all covariates tested
☒	☐	A description of any assumptions or corrections, such as tests of normality and adjustment for multiple comparisons
☐	☒	A full description of the statistical parameters including central tendency (e.g. means) or other basic estimates (e.g. regression coefficient) AND variation (e.g. standard deviation) or associated estimates of uncertainty (e.g. confidence intervals)
☐	☒	For null hypothesis testing, the test statistic (e.g. *F*, *t*, *r*) with confidence intervals, effect sizes, degrees of freedom and *P* value noted<br>*Give P values as exact values whenever suitable.*
☒	☐	For Bayesian analysis, information on the choice of priors and Markov chain Monte Carlo settings
☐	☒	For hierarchical and complex designs, identification of the appropriate level for tests and full reporting of outcomes
☐	☒	Estimates of effect sizes (e.g. Cohen's *d*, Pearson's *r*), indicating how they were calculated

Our web collection on statistics for biologists contains articles on many of the points above.

Software and code

Policy information about availability of computer code

Data collection	All datasets were obtained from the supplementary materials of their respective publications.
Data analysis	All the analyses were performed with open source software: Python 3.10, PyTorch 2.5.1, GPyTorch 1.14, BoTorch 0.13.0, Transformers 4.48.3, PEFT 0.14.0, RDKit 2023.9.4, DRFP 0.3.6, scikit-learn 1.2.2, NumPy 1.26.3, pandas 1.5.3, Matplotlib 3.8.0, seaborn 0.13.2, Weights & Biases 0.19.8.

For manuscripts utilizing custom algorithms or software that are central to the research but not yet described in published literature, software must be made available to editors and reviewers. We strongly encourage code deposition in a community repository (e.g. GitHub). See the Nature Portfolio guidelines for submitting code & software for further information.

Data

Policy information about availability of data

All manuscripts must include a data availability statement. This statement should provide the following information, where applicable:

- Accession codes, unique identifiers, or web links for publicly available datasets
- A description of any restrictions on data availability
- For clinical datasets or third party data, please ensure that the statement adheres to our policy

All benchmarks and datasets used, along with the code have been released at https://github.com/schwallergroup/gollum/. The open-weight language models are

publicly available via Hugging Face. The proprietary models evaluated only as direct-optimizer baselines (OpenAI GPT-5 and GPT-5-mini, Anthropic Claude Sonnet 4 and Claude 3.5 Haiku, Google Gemini 2.5 Pro and Gemini 2.5 Flash) are third-party services accessible through their providers' commercial APIs.

Research involving human participants, their data, or biological material

Policy information about studies with human participants or human data. See also policy information about sex, gender (identity/presentation), and sexual orientation and race, ethnicity and racism.

Reporting on sex and gender	Not applicable. This study involved computational experiments using optimization methods and language models and did not involve human participants, their data, or biological material.
Reporting on race, ethnicity, or other socially relevant groupings	Not applicable. This study involved computational experiments using optimization methods and language models and did not involve human participants, their data, or biological material.
Population characteristics	Not applicable. This study involved computational experiments using optimization methods and language models and did not involve human participants, their data, or biological material.
Recruitment	Not applicable. This study involved computational experiments using optimization methods and language models and did not involve human participants, their data, or biological material.
Ethics oversight	Not applicable. This study involved computational experiments using optimization methods and language models and did not involve human participants, their data, or biological material.

Note that full information on the approval of the study protocol must also be provided in the manuscript.

Field-specific reporting

Please select the one below that is the best fit for your research. If you are not sure, read the appropriate sections before making your selection.

☒ Life sciences ☐ Behavioural & social sciences ☐ Ecological, evolutionary & environmental sciences

For a reference copy of the document with all sections, see nature.com/documents/nr-reporting-summary-flat.pdf

Life sciences study design

All studies must disclose on these points even when the disclosure is negative.

Sample size	The study performed computational optimization across 23 diverse benchmarks spanning organic synthesis, materials science, process chemistry, and molecular design.<br><br>For the Buchwald-Hartwig benchmark (Figures 3, 4 & 5) and the 23 cross-domain benchmarks (Figure 2), performance was evaluated by averaging results over 20 independent seeds for each method.<br><br>Each optimization run was "cold-started" with 10 initial experimental points (randomly selected from below-median performing conditions) and ran for 50 experimental iterations.<br><br>For the direct LLM prompting benchmark (Figure 5), 10 independent optimization seeds were executed per product (50 runs total per model), with each run collecting 50 unique, in-space suggestions.
Data exclusions	No data points or experimental runs were excluded from the primary analyses.
Replication	All experiments were computational. Reproducibility was ensured by running all optimization benchmarks (e.g., in Figures 2-5) using multiple independent seeds (typically 20) with computational reproducibility guarantees through seed_everything from pytorch_lightning.<br><br>The results reported are the mean and standard error aggregated across these independent runs, which confirms the replication and quantifies the statistical variance of the methods' performance.
Randomization	This study involved computational experiments. Randomization was used to select the 10 initial "cold-start" points for each optimization run. These points were chosen randomly from the set of known experiments with below-median performance, ensuring an unbiased and challenging starting point for all evaluated methods.
Blinding	Blinding was not applicable to this study. This was a computational evaluation where all methods (e.g., GOLLuM, GP+DRFP, Bochemian) were algorithmically benchmarked against objective, pre-defined quantitative metrics ("Top 5% Coverage", R^2, NLPD). The evaluation was deterministic and not subject to human experimenter bias.

Reporting for specific materials, systems and methods

We require information from authors about some types of materials, experimental systems and methods used in many studies. Here, indicate whether each material, system or method listed is relevant to your study. If you are not sure if a list item applies to your research, read the appropriate section before selecting a response.

Materials & experimental systems

n/a	Involved in the study
☒	☐ Antibodies
☒	☐ Eukaryotic cell lines
☒	☐ Palaeontology and archaeology
☒	☐ Animals and other organisms
☒	☐ Clinical data
☒	☐ Dual use research of concern
☒	☐ Plants

Methods

n/a	Involved in the study
☒	☐ ChIP-seq
☒	☐ Flow cytometry
☒	☐ MRI-based neuroimaging

Plants

Seed stocks	Not applicable. This study did not involve any plant materials, seed stocks, or plant genotypes.
Novel plant genotypes	Not applicable. This study did not involve any plant materials, seed stocks, or plant genotypes.
Authentication	Not applicable. This study did not involve any plant materials, seed stocks, or plant genotypes.
