# Supplementary material for: Large language models as uncertainty-calibrated optimizers for experimental discovery
Source: Nat Mach Intell. 2026 Aug 28;8(9):1466–77. doi: 10.1038/s42256-026-01283-z (PMC13585549; doi:10.1038/s42256-026-01283-z)
Supplement: Supplementary file 3 [file 42256_2026_1283_MOESM3_ESM.pdf]

nature portfolio

Peer Review File

Large language models as uncertainty-calibrated optimizers for experimental discovery

Corresponding Author: Professor Philippe Schwaller

This manuscript has been previously reviewed at another journal. This document only contains information relating to versions considered at Nature Machine Intelligence.

This file contains all reviewer reports in order by version, followed by all author rebuttals in order by version.

Version 0:

Reviewer comments:

Reviewer #1

(Remarks to the Author)
There were several specific claims made that were unsubstantiated in the original manuscript that have now been removed; it is much stronger for it.

A primary concern I raised in the initial submission was that Table S3 showed that LLM finetuning with the adapter gave incremental performance gains for T5 and Qwen2-7B. The learned projection seemed to be primarily responsible for performance, not the marginal likelihood flowing back through to the LLM. The revised manuscript has tempered some of its core claims to acknowledge the strong performance of PLLM in many settings and treat the \phi-finetuning as an optional add-on. However, I still wonder about the claims of superiority and how it is presented:
- In Figure 3, panel b is titled “Uncertainty-guided finetuning unlocks better optimization” and implies that the likelihood gradients are an essential part of this. As discussed in the rebuttal letter, however, PLLM alone is a strong baseline. In the bar chart in Figure 3b, PLLM+T5 achieves nearly-identical (perhaps statistically identical) performance for both T5 and Qwen2-7B.
- In that figure, and in Figure S9, is there a statistically significant difference between PLLM+T5 and PLLM\phi+T5 performance even for the “Hard” tasks? Was this analyzed in addition to the win rate table?
- Relatedly, the authors’ reporting summary does not appear to have been filled out properly. Several of the “confirmed” statistical tests do not appear applicable to this paper, and many comparisons are discussed in the paper without performing statistical tests. I would encourage the authors to carefully go through this and respond to the individual elements.
- The exclusion of PLLM+T5 from Figure 4d further downplays that having a simple adapter may be sufficient to achieve most/all of the benefits of GOLLuM.
- The slight differences in performance could potentially be attributed to the increased model capacity. Adding more trainable parameters gives the model more capacity, so of course it has more room for improvement. I would be interested to see a parameter-matched random MLP control (add a MLP on top of frozen embeddings before GP kernel to check if the finetuning is really doing something more meaningful than just adding capacity).

I appreciate the authors’ inclusion of removing chemical terms in S5.5 (and the choice of chocolate making). What is shown is a learned organization of performance resulting from the data. What my original comment had asked about is whether/how this affects optimization performance to test the claim that the LLMs are getting information entirely from the new experimental data. This claim has been removed from the revised paper, so the point is moot. I do not believe this demonstrates the absence of data leakage.

I understand that the authors are not able to benchmark an infinite number of methods. However, I would suggest being careful about claiming that including representative examples fully covers entire families of methods. For example, stating that ICL-style approaches like BoLIFT aren’t needed because LAPEFT is included. There may be subtle but important distinctions that affect performance. I do think that ICL is a very compelling and important alternative, even if the comparison is out of scope for this work.

The revised text makes a comment about LLMs for optimization being overconfident and lacking uncertainty calibration. I

would argue that this is not something solved by this manuscript, as the accuracy of predictions as a surrogate model do not appear to be better than naïve GP baselines (shown in the SI). While accuracy does not always correlate with optimization performance, the empirical results seem to contradict the claim implied in the text and Figure 5b that suggestions are “calibrated”. Similarly, Table S9 shows that on several benchmarks (e.g., Additives 1-4, HPLC, Vapdiff), PLLM\phi+T5 has negative R^2 values but still has competitive optimization performance; this would suggest that the surrogate is poorly calibrated despite the optimizer succeeding. Moreover, GP+DRFP achieves better NLPD on most of these same tasks. This pattern is hard to reconcile with the paper's titular framing of "uncertainty-calibrated" optimization and warrants some discussion. It may be worth ensuring that the title matches the content of the work more closely.

There is at least one broken cross-reference still in the SI (Figure S14) that I noticed.

(Remarks on code availability)

Reviewer #2

(Remarks to the Author)

(Remarks on code availability)
The code is clear and usable with documentation.

Reviewer #3

(Remarks to the Author)
The manuscript presents a solid approach by integrating Large Language Models with Bayesian Optimization via Gaussian process marginal likelihood fine-tuning. The proposed GOLLuM framework offers a practical solution to mitigate LLM hallucinations and improve uncertainty calibration in experimental design. The empirical results across multiple benchmarks are convincing. Overall, the paper is well-written and methodologically sound.

Here are a few minor revision suggestions:
1. The paper mentions state-of-the-art performance across 23 tasks, but the main text does not explicitly list them all. Please include a brief summary table or a short paragraph in the main text clearly categorizing all 23 benchmarks to improve readability.
2. In Figures 2 and 4c, the high-dimensional latent space is visualized in 2D. However, the specific dimensionality reduction technique (e.g., t-SNE, UMAP, PCA) and its hyperparameters are omitted. Please specify this in the methodology or figure captions for reproducibility.
3. There are formatting inconsistencies and incomplete citations in the bibliography. For instance, capitalization varies for "SMILES" [56], and several references contain missing information indicated by literal "???" (e.g., Refs [10], [15], [54]). Please thoroughly proofread the references.

(Remarks on code availability)

Open Access This Peer Review File is licensed under a Creative Commons Attribution 4.0 International License, which permits use, sharing, adaptation, distribution and reproduction in any medium or format, as long as you give appropriate credit to the original author(s) and the source, provide a link to the Creative Commons license, and indicate if changes were made.
In cases where reviewers are anonymous, credit should be given to 'Anonymous Referee' and the source.
The images or other third party material in this Peer Review File are included in the article's Creative Commons license, unless indicated otherwise in a credit line to the material. If material is not included in the article's Creative Commons license and your intended use is not permitted by statutory regulation or exceeds the permitted use, you will need to obtain permission directly from the copyright holder.
To view a copy of this license, visit https://creativecommons.org/licenses/by/4.0/

We thank the editor and reviewers for their valuable inputs. We have carefully addressed every comment and respond point by point below; reviewer comments are in black and our responses in blue.

Reviewer 1

There were several specific claims made that were unsubstantiated in the original manuscript that have now been removed; it is much stronger for it.

A primary concern I raised in the initial submission was that Table S3 showed that LLM finetuning with the adapter gave incremental performance gains for T5 and Qwen2-7B. The learned projection seemed to be primarily responsible for performance, not the marginal likelihood flowing back through to the LLM. The revised manuscript has tempered some of its core claims to acknowledge the strong performance of PLLM in many settings and treat the \phi-finetuning as an optional add-on. However, I still wonder about the claims of superiority and how it is presented:

Answer:

We thank the reviewer. We back every comparison below with paired statistical tests and add the parameter-matched control that was requested. We would also like to address the central premise directly, because we believe the data resolve it.

On "the projection is primarily responsible, not the marginal likelihood flowing back to the LLM." The LLMφ variant is a direct counterexample: it finetunes the LLM via LoRA and has no projection layer at all, so it isolates the marginal-likelihood gradients into the LLM. Across the 23 benchmarks (Top 5% Coverage, 50 iters, 20 seeds):

variant	what is trained	mean coverage	vs static (Bochem+T5)	Wilcoxon p
Bochem+T5	none (static embeddings + GP)	26.5	---	---
PLLM+T5	projection only	33.5	+6.9 pp	1.7e-4
**LLMφ+T5**	**LoRA only (no projection)**	**35.2**	**+8.7 pp**	**3.7e-5**
PLLMφ+T5	LoRA + projection	36.3	+9.8 pp	1.2e-6

LLMφ with no projection improves over the static-embedding GP by +8.7 pp (p = 3.7e-5), comparable to the projection-only PLLM (+6.9 pp). The marginal likelihood to the LLM therefore unlocks substantial gains *on its own*; the projection is not "primarily responsible."

The robust conclusion is deep-kernel vs. static, not projection vs. LoRA: every variant that jointly trains an LLM-based representation through the GP marginal likelihood, whether it's projection (PLLM), LoRA (LLMφ), or both (PLLMφ), significantly beats the fixed-embedding GP (all $p \leq 3e-4$, paired Wilcoxon, n=23). The three lie within ~2.8 pp of one another: LLMφ vs PLLM (+1.7 pp, $p = 0.18$) and PLLMφ vs LLMφ (+1.1 pp, $p = 0.17$) are not significant, while PLLMφ exceeds PLLM by a modest but significant +2.8 pp ($p = 0.004$). This is why we present all three variants as our contribution. We also note that at the margin the evidence runs opposite to the concern raised: adding LoRA on top of the projection (PLLM→ PLLMφ) yields a significant gain (+2.8 pp, $p = 0.004$), whereas adding the projection on top of LoRA (LLMφ → PLLMφ) does not (+1.1 pp, $p = 0.17$), so if either component is "secondary," it is the projection, not the marginal-likelihood finetuning.

In Figure 3, panel b is titled "Uncertainty-guided finetuning unlocks better optimization" and implies that the likelihood gradients are an essential part of this. As discussed in the rebuttal letter, however, PLLM alone is a strong baseline. In the bar chart in Figure 3b, PLLM+T5 achieves nearly-identical (perhaps statistically identical) performance for both T5 and Qwen2-7B.

Answer:

We agree PLLM is a strong baseline, and we have reframed the panel to match the deep-kernel-vs-static conclusion above. We retitled Figure 3b from "Uncertainty-guided finetuning unlocks better optimization" to "LLM-based deep-kernel learning enables better optimization" as the gains come from training the LLM representation through the GP marginal likelihood (whether the trainable parameters sit in a projection, the LLM via LoRA, or both), all of which substantially exceed static embeddings. We have already stated explicitly that PLLM is a strong baseline. Additionally, the PLLMφ and PLLM perform comparably across LLM models with different architectures, consistent with the deep kernel, not a particular backbone, being the driver.

In that figure, and in Figure S9, is there a statistically significant difference between PLLM+T5 and PLLMφ+T5 performance even for the "Hard" tasks? Was this analyzed in addition to the win rate table?

Answer:

Yes, beyond the win-rate table we ran paired tests (Wilcoxon signed-rank). At the final budget (Top 5% Coverage, 50 iters, 20 seeds), across all 23 benchmarks PLLMφ+T5 significantly outperforms PLLM+T5: +2.8 pp, win 18/23, paired Wilcoxon $p = 0.0043$ (two-sided; 0.0022 one-sided).

On the Hard subset specifically, we tested it under both difficulty definitions used in the SI and the difference is not statistically significant (linearity-Hard, n=7: p=0.34; dispersion-Hard, n=8: p=0.055). On reflection, the difficulty stratification itself is not robust: the dispersion metric was computed in a 2D PCA projection of the embeddings, and in the full embedding space the GP kernel actually operates on, the dispersion ratio concentrates near 1 for nearly all tasks (high-dimensional distance concentration), so it does not meaningfully separate tasks. We have therefore removed the difficulty stratification and claims that "φ-finetuning helps more on harder/lower-linearity tasks" (main text and SI), since they are not statistically supported. We now report the significant aggregate PLLM→PLLMφ gain and the full per-benchmark breakdown, and frame φ-finetuning as a refinement that is significant on average.

Relatedly, the authors' reporting summary does not appear to have been filled out properly. Several of the "confirmed" statistical tests do not appear applicable to this paper, and many comparisons are discussed in the paper without performing statistical tests. I would encourage the authors to carefully go through this and respond to the individual elements.

Answer:

We have revised the reporting summary to (i) remove tests that do not apply, and (ii) for each comparison discussed, report the paired test used (Wilcoxon signed-rank as primary), n (seeds/benchmarks), and exact p-values, consistent with previous comments

The exclusion of PLLM+T5 from Figure 4d further downplays that having a simple adapter may be sufficient to achieve most/all of the benefits of GOLLuM.

Answer:

PLLM+T5 was not removed from Figure 4d, it was never part of it, by design. Figure 3 is where we compare the GOLLuM variants (PLLM, LLMφ, PLLMφ) head-to-head, and we then carry the best variant (PLLMφ+T5) through the remainder of the analysis. Figure 4 is dedicated to the *mechanism* of emergent contrastive organization in the latent space and its link to optimization. The panel 4d deliberately contrasts the adaptive model (PLLMφ+T5) against its static counterpart (fixed T5 embeddings) to illustrate that mechanism, not to re-run a variant comparison. The variant-level question the reviewer raises is therefore addressed in Figure 3, which we are strengthening with the paired significance tests above and where PLLM is explicitly presented as a strong baseline.

The slight differences in performance could potentially be attributed to the increased model capacity. Adding more trainable parameters gives the model more capacity, so of course it has more room for improvement. I would be interested to see a parameter-matched random

MLP control (add a MLP on top of frozen embeddings before GP kernel to check if the finetuning is really doing something more meaningful than just adding capacity).

Answer:

Thank you for raising this point. We added this control: an MLP on the frozen T5 embeddings whose trainable parameters (216,644: 768→260→64) match PLLMφ+T5's LoRA+projection budget (216,640 = 165,888 LoRA + 50,752 projection). It is identical to PLLM in all else (DeepGP, ExpectedImprovement, same LR/optimizer/seeds; 23 benchmarks × 20 seeds), differing only by the added projection capacity. Paired by benchmark+seed (Top 5% Coverage):

- MLP vs PLLM: $\Delta = -0.2$ pp, Wilcoxon $p = 0.38$ (not significant): added capacity on frozen embeddings does not improve over the linear projection.
- MLP vs PLLMφ: $\Delta = -3.1$ pp, Wilcoxon $p = 1.6\text{e-}8$: the capacity-matched control stays significantly below the finetuned model.

So PLLMφ's advantage is not attributable to additional trainable capacity: a parameter-matched MLP on a fixed representation is statistically indistinguishable from the linear baseline, whereas finetuning the LLM (changing the representation itself, under a low-rank/pretrained-anchored constraint) is what improves optimization. We added this as a control panel and a paragraph in the SI.

We added a new SI figure comparing the projection-embedding family {MLP+T5, PLLM+T5} against the φ-finetuned family {LLMφ+T5, PLLMφ+T5} per benchmark (plus an averaged panel), with each family's best-of-two envelope marked. The φ-envelope exceeds the frozen-envelope on 19/23 benchmarks (mean $\Delta\varphi = +3.0$ pp, Wilcoxon $p = 3.2\text{e-}4$).

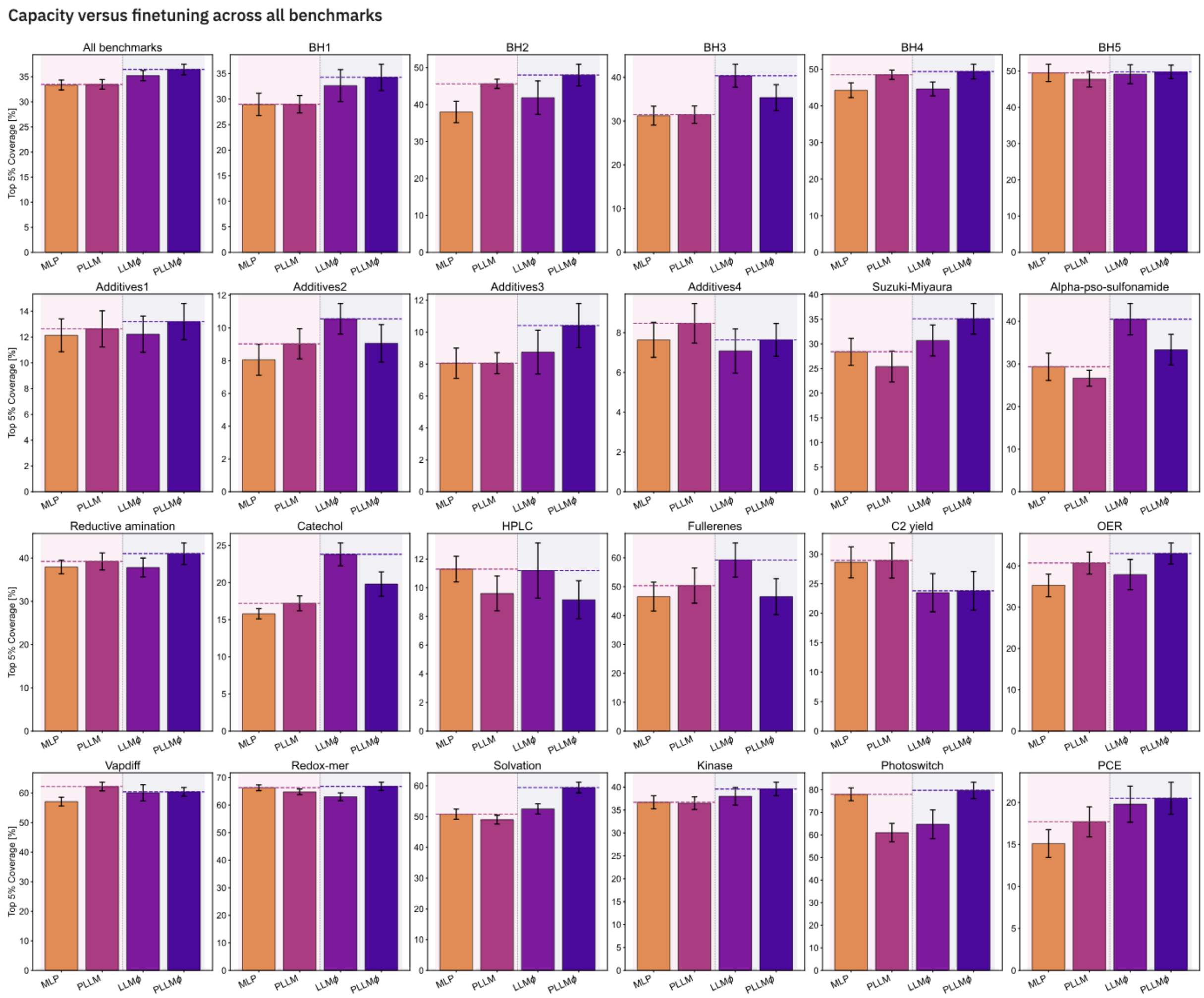

Fig. S16 Capacity versus finetuning. Top-5% coverage [%] at 50 iterations (mean $\pm$ SEM, 20 seeds) for the projection-embedding family {MLP+T5 (parameter-matched random MLP), PLLM+T5 (linear projection)} and the ϕ-finetuned family {LLMϕ+T5 (LoRA, no projection), PLLMϕ+T5 (LoRA + projection)}, per benchmark plus an equal-weight average. The pink dashed line marks the best of the projection pair (MLP, PLLM); the purple dashed line marks the best of the finetuned pair (LLMϕ, PLLMϕ); shading distinguishes the two families. The capacity-matched MLP tracks the linear PLLM ($\Delta = -0.2$ pp, $p = 0.38$) and stays below the ϕ family; the ϕ-envelope exceeds the projection-envelope on 19/23 tasks (mean +3.0 pp, $p = 3.2 \times 10^{-4}$).

On mechanism and recommendation. We are deliberately careful not to overclaim a single mechanism: the control rules out "just more trainable parameters," but we do not claim to isolate the exact source of the with-vs-without-φ difference. Our practical recommendation is therefore:

- use PLLMφ as the default when adapting to a new optimization task as it provides the most degrees of freedom to reshape the representation through the GP marginal likelihood and is the strongest variant in aggregate;

- use PLLM as a strong, substantially cheaper alternative when the cost of LoRA finetuning is prohibitive for a given user, since it recovers most of the benefit (both far exceed static embeddings);
- use LLMφ when you don't want to think about additional projection hyperparameters at all.

I appreciate the authors' inclusion of removing chemical terms in S5.5 (and the choice of chocolate making). What is shown is a learned organization of performance resulting from the data. What my original comment had asked about is whether/how this affects optimization performance to test the claim that the LLMs are getting information entirely from the new experimental data. This claim has been removed from the revised paper, so the point is moot. I do not believe this demonstrates the absence of data leakage.

Answer:

We thank the reviewer. Our original sentence, and our reading of the original comment, concerned the mechanism: the reorganization of the latent space is driven by the experimental outcomes, not by explicit chemical instruction. This outcome-driven organization is what improves optimization, as we show directly against the base (fixed) LLM embeddings in Figure 3. Those embeddings carry the prior but lack the outcome-aligned geometry and are the weakest variant. Consistently, relabeling the task removes the chemical naming yet leaves optimization performance statistically unchanged (34.25% vs 31.25% top-5% discoveries, $\Delta = -3$pp, Mann–Whitney $p = 0.46$). We may be interpreting "data leakage" differently, but these improvements come from learning a better optimization structure and cannot be attributed to the base LLM prior. If that was the source, BoChemian (fixed embeddings + GP) would already perform comparably.

I understand that the authors are not able to benchmark an infinite number of methods. However, I would suggest being careful about claiming that including representative examples fully covers entire families of methods. For example, stating that ICL-style approaches like BoLIFT aren't needed because LAPEFT is included. There may be subtle but important distinctions that affect performance. I do think that ICL is a very compelling and important alternative, even if the comparison is out of scope for this work.

Answer:

We agree that in-context learning (ICL) is a very compelling direction, and we note that we do engage with in-context optimization through our LLM-as-optimizer baseline, which conditions on the design space and the full experimental history in context to propose the next experiment. The more characteristic ICL-BO methods such as BoLIFT, however, come with the additional considerations we noted previously: BoLIFT uses a frozen LLM as an in-context regression surrogate, providing previous experiments as few-shot examples in

the prompt and reading off predictive uncertainty from token probabilities / multiple completions, which requires an LLM query (often to a large proprietary model) for every candidate scored, with prompts that grow as history accumulates and entail example-selection/ordering/truncation choices, which can make the methods succeed or fail. This makes acquisition substantially more expensive and more prompt-sensitive when the design space is large. And while we do benchmark LAPEFT as a fully comparable class, we agree it does not subsume the ICL-BO family.

The revised text makes a comment about LLMs for optimization being overconfident and lacking uncertainty calibration. I would argue that this is not something solved by this manuscript, as the accuracy of predictions as a surrogate model do not appear to be better than naïve GP baselines (shown in the SI). While accuracy does not always correlate with optimization performance, the empirical results seem to contradict the claim implied in the text and Figure 5b that suggestions are “calibrated”. Similarly, Table S9 shows that on several benchmarks (e.g., Additives 1-4, HPLC, Vapdiff), PLLM\phi+T5 has negative R^2 values but still has competitive optimization performance; this would suggest that the surrogate is poorly calibrated despite the optimizer succeeding. Moreover, GP+DRFP achieves better NLPD on most of these same tasks. This pattern is hard to reconcile with the paper's titular framing of "uncertainty-calibrated" optimization and warrants some discussion. It may be worth ensuring that the title matches the content of the work more closely.

Answer:

First, we agree that R^2 is not the appropriate metric for a BO surrogate, and we dedicate a section to this in the SI (S5.6 Structure vs fit and BO performance): optimization success tracks representation smoothness ($r = 0.92$), not pointwise fit ($r = 0.78$).

Second, on calibration (NLPD), the picture across Table S9 is mixed rather than one-sided. On the descriptor-rich tasks the GP is well-calibrated, but its calibration is contingent on a good descriptor: on descriptor-poor tasks it falls back to numerical parameters and its NLPD reaches 10^3–10^5 (HPLC, VapDiff, C2); PLLMφ+T5's worst case across all tasks is ≈34. Consequently, GOLLuM attains the lowest mean NLPD compared to all baselines, with substantive robustness and no catastrophic calibration failure, even without any task-specific descriptors.

Third, the residual gap on the additives tasks is a side-effect of a single global adaptation rate: training parameters were tuned once on BH1 and applied systematically across all 23 benchmarks, which slightly over-adapts the lowest-signal tasks. Re-running those benchmarks at a lower LLM learning rate improves NLPD toward GP levels with no loss of optimization performance (Top-5% coverage unchanged).

Benchmark	PLLMφ+T5 LLM lr 2×10^{-3} R^2 / NLPD	PLLMφ+T5 LLM lr 2×10^{-4} R^2 / NLPD	GP R^2 / NLPD
Additives-1	−0.00 / 18.03	+0.04 / 12.49	0.09 / 11.34
Additives-2	−0.22 / 20.60	−0.11 / 12.05	0.01 / 10.00
Additives-3	−0.30 / 25.95	−0.19 / 13.14	−0.01 / 10.36
Additives-4	−0.34 / 18.15	−0.17 / 11.53	0.01 / 10.27
HPLC	+0.03 / 14.58	−0.09 / 8.84	-0.04 / 1444.58
Vapdiff	+0.01 / 8.11	+0.06 / 2.18	0.12 / 608876.27

There is at least one broken cross-reference still in the SI (Figure S14) that I noticed.

Answer:

We have fixed the broken reference to Figure S14 and re-checked all SI cross-references.

Reviewer 3

The paper mentions state-of-the-art performance across 23 tasks, but the main text does not explicitly list them all. Please include a brief summary table or a short paragraph in the main text clearly categorizing all 23 benchmarks to improve readability.

Answer:

We have added both. (1) A short paragraph in the main text now categorizes all 23 benchmarks by chemistry domain, and (2) a complete Benchmark overview table in the SI that lists every task with its dataset size, optimization objective, and source.

Table S2 Benchmark overview. The 23 optimization tasks grouped by chemistry domain, with dataset size (n) and optimization objective.

Domain	Benchmark	n	Objective
Organic chemistry	Buchwald–Hartwig (BH1–BH5) [29]	≈791	maximize yield (per aryl-halide product; BH1–BH5)
	Additive screens (Additives1–4) [59]	720	maximize product response (Ni-catalysed)
	Suzuki–Miyaura [60]	5760	maximize cross-coupling yield
	Pd-sulfonamide coupling [61]	585	maximize AP yield
	Reductive amination [62]	768	maximize conversion
Analytic & process chemistry	Catechol rearrangement [63]	1227	maximize scalarized yield/selectivity
	HPLC [64]	1386	maximize chromatographic peak response
	Fullerenes (C_{60} adducts) [65]	246	maximize product mole fraction
Materials & catalysis	C2 yield [3]	1180	maximize C2 yield (methane oxidative coupling)
	OER [64]	2121	minimize catalyst overpotential
	Vapdiff [64]	918	maximize crystal score
Molecular property optimization	Redox-mer [66]	1407	minimize redox potential
	Solvation [66]	1407	minimize solvation energy
	Kinase [67]	10449	minimize docking score (kinase inhibitors)
	Photoswitch [68]	392	maximize π–π^* transition wavelength
	PCE [69]	10000	maximize power conversion efficiency

In Figures 2 and 4c, the high-dimensional latent space is visualized in 2D. However, the specific dimensionality reduction technique (e.g., t-SNE, UMAP, PCA) and its hyperparameters are omitted. Please specify this in the methodology or figure captions for reproducibility.

Answer:

We now specify this in both the figure captions and a dedicated Latent-space visualization paragraph in the SI Implementation details. The 2D latent-space panels (Figs. 2 and 4c, and SI Fig. gp-fit-dist-tsne) are t-SNE projections (scikit-learn) with perplexity 50, learning rate 200, 2000 iterations, Euclidean distance metric, and random seed 42 (scikit-learn default initialization). We also clarify that the reduction is used purely for visualization; all quantitative analyses operate on the full-dimensional embeddings.

There are formatting inconsistencies and incomplete citations in the bibliography. For instance, capitalization varies for "SMILES" [56], and several references contain missing information indicated by literal "???" (e.g., Refs [10], [15], [54]). Please thoroughly proofread the references.

Answer:

We have proofread the bibliography, updated "SMILES" capitalization, and filled the missing fields in the flagged references, with a full consistency pass.
